# Anthranilic Acid Inhibitors of Undecaprenyl Pyrophosphate Synthase (UppS), an Essential Enzyme for Bacterial Cell Wall Biosynthesis

**DOI:** 10.3389/fmicb.2018.03322

**Published:** 2019-01-14

**Authors:** Marko Jukič, Kaja Rožman, Matej Sova, Hélène Barreteau, Stanislav Gobec

**Affiliations:** ^1^Faculty of Pharmacy, University of Ljubljana, Ljubljana, Slovenia; ^2^Bacterial Cell Envelopes and Antibiotics Group, Institute for Integrative Biology of the Cell (I2BC), CEA, CNRS, Université Paris-Sud, Université Paris-Saclay, Gif-sur-Yvette, France

**Keywords:** UppS, inhibitors, cell-wall, pharmacophore model, antibacterial agents, undecaprenyl pyrophosphate synthase

## Abstract

We report the successful implementation of virtual screening in the discovery of new inhibitors of undecaprenyl pyrophosphate synthase (UppS) from *Escherichia coli.* UppS is an essential enzyme in the biosynthesis of bacterial cell wall. It catalyzes the condensation of farnesyl pyrophosphate (FPP) with eight consecutive isopentenyl pyrophosphate units (IPP), in which new *cis*-double bonds are formed, to generate undecaprenyl pyrophosphate. The latter serves as a lipid carrier for peptidoglycan synthesis, thus representing an important target in the antibacterial drug design. A pharmacophore model was designed on a known bisphosphonate **BPH-629** and used to prepare an enriched compound library that was further docked into UppS conformational ensemble generated by molecular dynamics experiment. The docking resulted in three anthranilic acid derivatives with promising inhibitory activity against UppS. Compound **2** displayed high inhibitory potency (IC_50_ = 25 μM) and good antibacterial activity against *E. coli* BW25113 Δ*tolC* strain (MIC = 0.5 μg/mL).

## Introduction

The alarming increase in number of resistant bacterial strains is forcing academia and pharmaceutical companies into a hasten development of new antibacterial drugs. Therefore, new design approaches leading to discovery of new compounds, mechanisms of action or even new bacterial targets are desirable (Van Geelen et al., 2018). One of the most recent and fairly underexplored targets is UppS (EC: 2.5.1.31) (Jukic et al., 2016).

Undecaprenyl pyrophosphate synthase is an essential cytoplasmic enzyme in the biosynthesis of peptidoglycan that catalyzes the formation of isoprenoid UPP (C_55_-PP) from FPP and IPP in the presence of Mg^2+^. UPP is a constituent of lipid II, the last peptidoglycan precursor, which is responsible for the flip-flop of the Glc*N*Ac-Mur*N*Ac-pentapeptide moiety across the cytoplasmic membrane (Liang et al., 2002; Teng and Liang, 2012b). The enzyme is specific for the bacteria and is not present in the human cell, thus representing an important target in the development of novel antibacterial agents (Apfel et al., 1999). Despite the many published crystal structures of apo enzyme (Ko et al., 2001) or enzyme co-crystalized with substrates (Chang et al., 2004) and inhibitors (Guo et al., 2007), there is still no registered drug targeting UppS (Jukic et al., 2016).

There are currently 40 crystal structures in the PDB. Historically, first two published structures came from from Micrococcus luteus (PDB ID: 1F75) (Fujihashi et al., 2001) and Escherichia coli (PDB ID: 1JP3) (Ko et al., 2001) and were published back in 2001. Nowadays, the majority of reported crystal complexes are from the *E. coli*, but all of the reported structures belong to the same sequence similarity cluster with > 40% similarity and include Gram positive and Gram negative bacteria. Analysis of available crystal structures shows that UppS is a homodimer composed of two identical subunits, each composed of approximately 250 amino acids in length, totaling to 29 kDa. The essential information about the subunits is the extensive movement of the enzyme core and most importantly the loop at the top of the active site. More specifically, while the substrate is bound to the enzyme, the active site remains closed, however, it normally opens during the product binding before it is released (Fujihashi et al., 2001; Ko et al., 2001).

The active site of UppS is particularly large due to a rather sizable final product (55 carbon atoms), which needs to be accommodated at the catalytic gorge. Thus, it is not unexpected that the active site is shaped as a long tunnel along the length of enzyme core. The complexity of this active site is a challenge for the pharmaceutical chemists, because it can accommodate a greater number of small-molecule inhibitors and also possesses several disctinct binding sites. This information has to be taken into account during an *in silico* design of new UppS inhibitors (Teng and Liang, 2012a; Kim et al., 2014).

**FIGURE 1:**
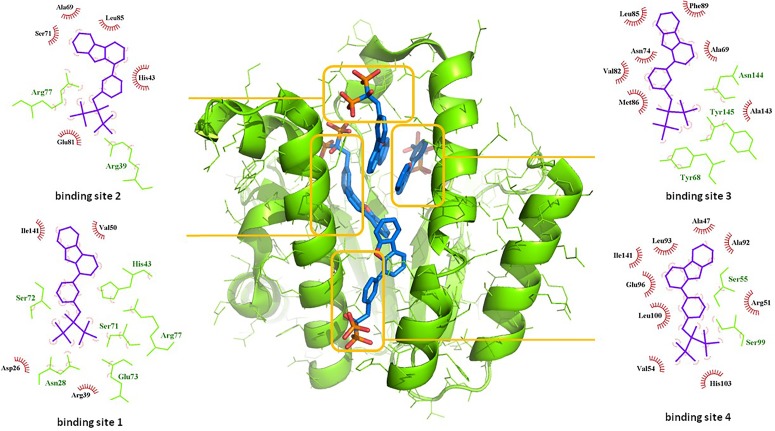
Binding sites of co-crystalised bisphosphonate inhibitor (depicted in blue stick model; BPH-629) in *E. coli* UppS (presented as a green-colored ribbon model; PDB ID: 2E98). Four observed binding sites (emphasized) are presented with small-molecule inhibitor in blue, amino acid residues forming polar contacts in green with residues that form lipophilic interactions in red.

Among the most potent UppS inhibitors are bisphosphonates, traditionally indicated for bone-related diseases, namely suppressing bone resorption and bone loss. Ever since the pamidronate FDA approval in 1991, bisphosphonates have been widely prescribed, yet the precise mechanistic properties are still unclear (Allen, 2018). Surprisingly, the most recent studies suggest that bisphosphonates are promising opioid alternatives for the treatment of chronic pain, more specifically the complex regional pain syndrome type I (CRPS-I); however, this mechanism of action also needs to be clarified (Kaye et al., 2018). Ultimately, it has been shown that some bisphosphonates bind to and inhibit UppS. Their structure mimics the pyrophosphate moiety of the substrates IPP and FPP, thus indicating the possible mechanism of action, which was solidified with published co-crystal structures (Figure 1). With 40 available crystal structures, 19 report small-molecule inhibitors and amongst them, 6 are with bisphosphonate inhibitors (Guo et al., 2007). Among all of 40 reported structures, crystal complex of bisphosphonate inhibitor BPH-629 in *E. coli* UppS (PDB ID: 2E98) displays the highest resolution of 1.9 Å and was used in our work. One of the four known binding sites of bisphosphonates coincides with the FPP binding site, shown as binding site 1 on Figure 1.

Over the years, there has been a few *in silico* studies performed on UppS in an attempt to design new UppS inhibitors (Kuo et al., 2008; Peukert et al., 2008; Kim et al., 2014; Sinko et al., 2014). They have been successful only to some extent due to the complexity of the enzyme dynamics and high flexibility of the enzyme. However, bisphosphonates remain the most visible inhibitors to this day (Jukic et al., 2016).

**FIGURE 2:**
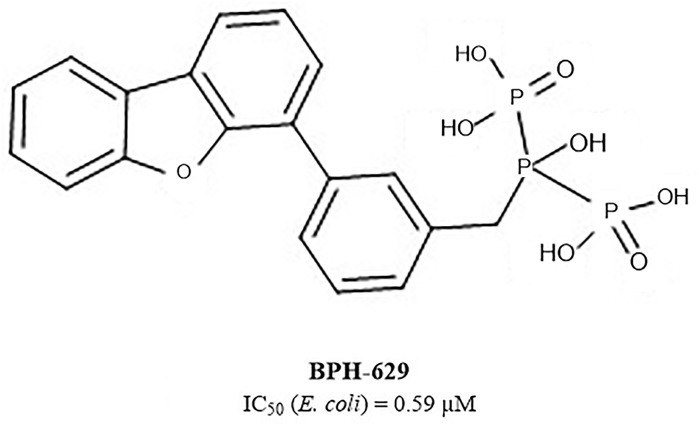
Structure of bisphosphonate inhibitor BPH-629.

Another extensive study of UppS active site flexibility using a molecular dynamics simulation showed the importance of the so called expanded pocket for the computer-aided drug design (Sinko et al., 2011). This expanded pocket state occurs during the ligand binding and reaches up to a total volume of 1032 Å^3^, as could be seen in a co-crystal structure of bisphosphonate **BPH-629** (Figure 2) with *E. coli* UppS (PDB: 2E98). Upon ligand removal the active site pocket shrinks down to a volume of 432 Å^3^, which is slightly larger than a final volume of an *apo*-UppS form (332 Å^3^, PDB: 3QAS). These types of inhibitors compared to non-bisphosphonates need a greater active site expanding due to the nature of their multiple binding. For example, the known tetramic acids and dihydropyridin-2-one-3-carboxamide inhibitors (Peukert et al., 2008), which bind to FPP binding site (Binding site 1; Figure 1), only require an active site of approximately 300 Å^3^ in volume. This implies that of the known UppS inhibitors, only bisphosphonates bind to an open enzyme form, while others bind to the closed form, which is similar to the non-ligand bound *apo* state (Sinko et al., 2011). The expanded pocket of the open enzyme form was thus proven to be the most suitable for molecular docking.

In this paper, we present a combination of pharmacophore design and molecular dynamics as a possible approach for discovery of new UppS inhibitors. For this purpose a known crystal structure of the enzyme with the bisphosphonate **BPH-629** (PDB ID: 2E98) was taken as a starting point for the design of new UppS inhibitors.

## Materials and Methods

### Cloning, Overexpression, and Purification of the *E. coli* UppS

An overnight preculture of *E. coli* C43(DE3) carrying the pET2130::*uppS_Ec_* plasmid was used to inoculate 1 liter of 2YT medium supplemented with ampicillin. The culture was incubated with shaking at 37°C until the optical density at 600 nm reached 0.8. Isopropyl-β-D-thiogalactopyranoside (IPTG) was added to a final concentration of 1 mM and incubation was continued for 3 h at 37°C. The cells were then harvested at 4°C and the pellet was washed with buffer A (20 mM Hepes (pH 7.5), 150 mM NaCl). The cells were resuspended in the same buffer (10 mL) and disrupted by sonication in the cold using a Bioblock Vibracell 72412 sonicator. The resulted suspension was centrifuged at 4°C for 30 min at 100,000 × *g* with a Beckman TL100 apparatus and the pellet was discarded. The supernatant was kept at −20°C until purification.

The N-terminal His_6_-tagged UppS*_Ec_* protein was purified on Ni^2+^-nitrilotriacetate (Ni^2+^-NTA) agarose according to Qiagen^®^ recommendations. All procedures were performed at 4°C. To perform the binding experiment, the supernatant was mixed with Ni^2+^-NTA-agarose beads for 1 h that had previously been washed with buffer B (buffer A containing 10 mM imidazole). The washing and elution steps were performed with a discontinuous gradient of imidazole (10 to 250 mM) in buffer A. Eluted proteins were analyzed by sodium dodecyl sulfate-polyacrylamide gel electrophoresis (SDS-PAGE) and the relevant fractions were pooled and dialyzed into 100 V of buffer A. The protein concentration was determined by nano-volume spectrophotometry (molecular mass of Nter-His_6_ UppS = 29,542 Da; ε_M_ = 38,960 M^−1^.cm^−1^). For the storage of the protein at −20°C, glycerol was added to the buffer to a final concentration of 10%.

### UppS Inhibition Assay

The UppS enzymatic activity was determined by using a kinetics-based assay utilizing a radiolabeled substrate. The assay revolves on the measuring of UPP formation in the reaction mixture in a final volume of 40 μL. Stock solutions of all compounds (2 mM) were prepared in DMSO and the final concentration of DMSO in the assay was 5% (v/v). The enzyme was diluted in buffer A to appropriate concentration so that the consumption of the substrate in the assay is no higher than 30%. The reaction mixture consisted of 20 μL of 100 mM HEPES, pH 7.5, 50 mM KCl, 0.5 mM MgCl_2_, 1.5 μM FPP, 12 μM [^14^C]-IPP ([^14^C]-IPP; 289 Bq), 2 μL DMSO with or without the inhibitor and 18 μL of optimal enzyme solution. The reaction was initiated by adding the enzyme to the reaction mixture and was observed for 30 min at 25°C before being stopped by freezing with liquid nitrogen. Reaction mixture was lyophilized and resuspended in 10 μL of purified water. The radiolabeled substrate, [^14^C]-IPP, and the product, [^14^C]UPP, were separated on a Silica gel 60 TLC plate using 1-propranol / ammonium hydroxide / water in ratio of 6/3/1 (v/v/v) as a mobile phase (Rf_([_^14^_C]–IPP)_ = 0.21, Rf_[_^14^_C]UPP_ = 0.56), and quantified with a radioactivity scanner (Rita Star, Raytest Isotopenmessgeräte GmbH, Straubenhardt, Germany). Residual activities (RAs) were calculated with respect to a control reaction without the tested compounds and with 5% DMSO. All the experiments were run in duplicate with standard deviations within ± 10%. The IC_50_ values represented the concentrations for which the RA was 50% and were determined by measuring the RAs at seven different compound concentrations.

### Microbiological Evaluation

The three compounds 1, 2, and 3 were tested for their antibacterial activity against the WT and efflux pump-deficient (Δ*acrA*, Δ*acrB* and Δ*tolC*) *E. coli* BW25113 strain (Table 1). The strains were cultivated in liquid medium at 37°C and inoculated in a 3-mL top agar at a final concentration of 10^8^ CFU/mL on agar plates. Then, spots of 4 μL of each compound (range concentration serially diluted from 32 μg/mL to 0.5 μg/mL) were performed on each strain, in the presence or not of 0.025 μg/mL of polymyxine B to assess the impact of outer-membrane permeability on antimicrobial activity. Finally, the antibacterial activity was observed after incubating the plates ON at 37°C. All the experiments were performed according to CLSI guidelines.

**Table 1:** Antibacterial activity of the most potent three UppS inhibitors against wild-type and efflux pump-deficient *E. coli* BW25113 strains with (+PMB) or without (−PMB) polymyxin B-formed permeable membrane.

	MIC (μg/mL)								
	BW25113		BW25113 Δ*acrA*		BW25113 Δ*acrB*		BW25113 Δ*tolC*	
	−PMB	+PMB	−PMB	+PMB	−PMB	+PMB	−PMB	+PMB
1	>32	>32	>32	2	>32	2	>32	>32
2	>32	>32	>32	>32	>32	>32	0.5	0.5
3	>32	>32	>32	2	>32	2	>32	>32

## Results and Discussion

### Pharmacophore Modeling

Docking of large libraries of compounds is not only complex but also time-consuming. Therefore, it is highly important to use a quality compound database for any *in silico* drug design. We used ZINC database of compounds, specifically 10.7 million Drugs Now subset of the ZINC library where compounds with immediate commercial availability are collected (Irwin and Shoichet, 2005). Prior to our docking experiment, hierarchical filtering of the compound database was performed. Database was first processed with the FILTER software (OpenEye Scientific Software, Inc., Santa Fe, NM, United States^1^) to eliminate small fragments or molecules with a greater MW than 1000 g/mol, known or predicted aggregators and the compounds with predicted poor solubility (Shoichet, 2006). Compound retention parameters used were 300 ≤ MW ≤ 1000, 0 ≤ rotational bonds ≤ 15, 4 ≤ rigid bonds ≤ 55, −4 ≤ clogP ≤ 6.85; detailed filter configuration can be found in supporting information. Finally, compound database was filtered for PAINS using RDKit^2^ Python API software (Baell and Holloway, 2010). In this final step, every structure in the library was compared to the selection of PAINS structures defined in SMARTS format and removed from the database if found similar (Saubern et al., 2011; PAINS definitions in SMARTS format can be found in supporting info.). The initial compound library was thus reduced to a library of approximately 6.5 million compounds and 3D conformer database prepared with omega2 fast protocol within LigandScout as detailed in the supporting information.

**FIGURE 3:**
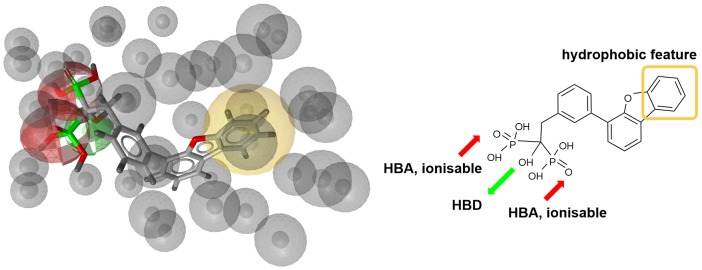
Left: 3D representation of a pharmacophore model based on inhibitor BPH-629 (green spheres represent H-bond donating feature (HBD), red spheres represent H-bond acceptor feature (HBA) while yellow-colored sphere represents a lipohilic feature. Gray colored spheres are exclusion cones based on crystal structure PDB ID: 2E98); right is a 2D projection of BPH-629 inhibitor with explicitly defined pharmacophoric features.

Next step was pharmacophore modeling in a consecutive library filtering effort in order to produce an enriched library for docking experiments. Pharmacophore model (Figure 3) was designed using LigandScout program (Wolber and Langer, 2005) based on the structural data of known bisphosphonate inhibitor **BPH-629** (Figure 2) binding mode in UppS binding site 1 (Figure 1; PDB ID: 2E98). Specific features of the inhibitor were used to pinpoint the previously described key interactions with the enzyme (Jukic et al., 2016). Ten similar pharmacophore models were generated and validated in a VS experiment using a library of reported bisphosphonates (Guo et al., 2007) and decoy compounds generated on the basis of each active bisphosphonate with the help of DUD-E database (Mysinger et al., 2012). The best model according to ROC AUC was used for filtering of prepared compound library (Figure 3). The model was defined by specifically negative ionisable features and/or H-bond acceptors at the **BPH-629** bisphosponic acid moiety, aliphatic hydroxyl group was marked as hydrophilic H-bond donor feature and distant aromatic ring as a hydrophobic feature in order to keep the pharmacophore model feature count low and produce a useful model for future filtering (Wolber and Langer, 2005). The exclusion zones calculated by the software on the basis of crystallized **BPH-629** binding mode (PDB ID: 2E98) have been included in the final pharmacophore model. If additional lipophilic features were used in the pharmacophore model or tolerance spheres defined closely along the **BPH-629** features, constructed models proved to be over-defined and could not be used as a filter in future steps. In the final step, initial compound library was filtered where individual conformer molecular features were enumerated and a 3D superposition on the pharmacophore model was attempted where pharmacophoric elements had to be satisfied within the defined spherical bounds and one possible missing feature (LigandScout software Pharmacophore-Fit scoring function). The exclusion zones further limited the space available for the individual conformer to superpose and satisfy the pharmacophore model. Thus a final library of 13530 compounds was prepared and docked in the protein conformation ensemble obtained from MD experiment and clustering of protein conformations along the MD trajectory (see Supplementary Data for more details).

### Molecular Dynamics

**FIGURE 4:**
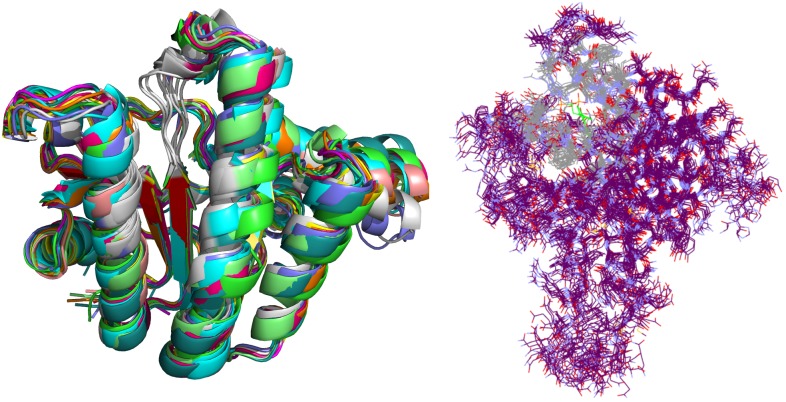
Selected snapshots of *E. coli* UppS obtained with MD and ClusCo clustering. Individual chains are presented in ribbon model colored distinctly for every snapshot used in the ensemble docking experiment. In this manner, movement of the protein along the MD trajectory is emphasized and observed (left). Defined binding site for GOLD ensemble docking experiment in gray-colored line model representation; in green colored line representation there is the center residue of the defined binding site for docking experiment while the rest of the protein is depicted as purple colored line model (right).

Crystal complex (PDB ID: 2E98) was prepared with Yasara software (Krieger and Vriend, 2015). Missing hydrogens were added, overlapping atoms adjusted, missing residues modeled, hydrogen bonds optimized and residue ionization assigned at pH = 7.4, consistently with previous reports (Krieger et al., 2006; Krieger et al., 2012; Jukic et al., 2016). Cubic system (10 Å around all atoms) was solvated using TIP3P water model and 0.9% of NaCl added to the solvation system. Finally, NPT (periodic boundary conditions) ensemble production run at 310 K was initiated. Simulation using AMBER14 force field produced 20 ns trajectory with snapshot saved every 10 ps (Hornak et al., 2006). Energy parameters of the system were stable through production run as was root-mean-square deviation (RMSD) values for protein backbone. MD snapshots in 100 ps increments were collected (200 protein conformation models), clustered using ClusCo software and visually analyzed with Pymol 3 software (DeLano, 2002). ClusCo software parameters used were hierarchical clustering in a pairwise average-linkage manner with backbone rmsd score. 10 clusters were identified by ClusCo and centroid structures were selected as protein conformations that represent the movement of the *E. coli* UppS (Figure 4, left) (Jamroz and Kolinski, 2013).

### Structure-Based Virtual Screening

**FIGURE 5:**
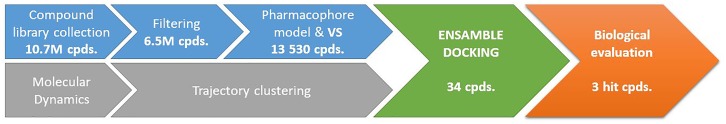
Completed workflow used for identification of UppS inhibitors. Number of processed compounds (cpds.) is indicated under individual steps.

Ensemble docking experiment (Figure 4, right) was performed using GOLD (CCDC Enterprise; 5.5 version). Ten protein structures obtained from clustered (ClusCo) MD trajectory were aligned to the first structure used in the trajectory, imported in Hermes GOLD where hydrogens were corrected/added. Positioning of Asn, Gln, and His tautomers were left intact as calculated through MD experiment. Waters and Ligands were removed and the proteins were kept rigid during docking experiment. Binding site was defined as a 7 Å region around the area occupied by BPH-629 co-crystalized ligand in the binding site 1 relative to the spacing of first structure used in the trajectory (Figure 1). Detect cavity setting was used and all H-bond donors/acceptors were forced to be treated as solvent accessible. All planar R-NR_1_R_2_ were able to flip as well as protonated carboxylic acids. Torsion angle distributions and rotatable bond postprocessing were set at default. Docking was performed with Chemscore scoring function with early termination enabled and default GOLD parameter file used. Genetic algorithm settings were set at ensemble. Parallel gold calculation was performed with concatenation of results and retention of best binding poses. No constraints were used in docking experiment. The results were analyzed using DataWarrior software and sorted according to the GOLD ChemScore Fitness. From the entire workflow as composed in Figure 5, the 34 top-scoring compounds were purchased from several vendors (see Supplementary Data and Supplementary Table S1) and evaluated biochemically and microbiologically.

### Biological Evaluation

**FIGURE 6:**
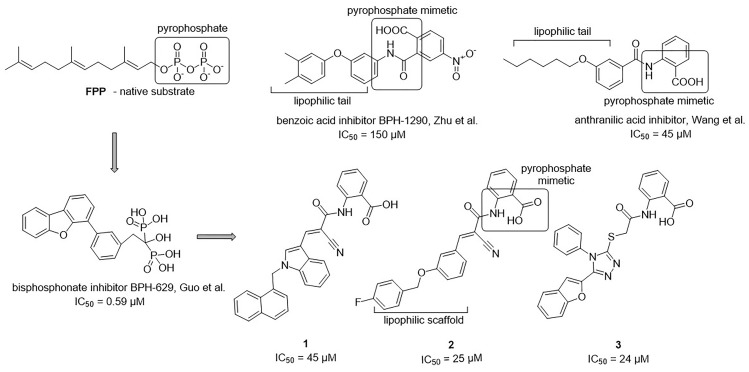
Structural comparison of UppS native substrate farnesyl pyrophosphate (FPP), benzoic acid inhibitor (Zhu et al., 2013), anthranilic acid inhibitor (Wang et al., 2016), bisphosphonate inhibitor (Guo et al., 2007) and new inhibitors of *E. coli* UppS discovered by structure-based virtual screening.

The 34 purchased compounds were tested for their inhibitory potencies against *E. coli* UppS using a radioactivity-based assay. In these test conditions, the [^14^C]-UPP formation is observed thanks to a radiolabeled substrate ([^14^C]-IPP) and quantified with a radioactivity scanner. The results are presented as RAs of UppS in the presence of 100 μM of each compound (Supplementary Table S1). For the compounds with RAs below 50%, the IC_50_ values were determined. Sodium risedronate was used as a positive control to enable the comparison of the purchased compounds to a known inhibitor and to confirm the results of the UppS inhibition assay. Three compounds showed promising inhibitory potencies against *E. coli* UppS in micromolar range (**1–3**, Figure 6). All three inhibitors are anthranilic acid derivatives with a larger hydrophobic moiety attached to the amide group *via* different linkers, 2-cyanoacryloyl for compounds **1** and **2** and 2-thioacetyl for compound **3**. Of those, compound **1** was the highest ranking virtual screening hit with ChemScore GOLD Fitness ChemScore of 41.82 and an IC_50_ value of 45 μM. On the other hand, the compound **3** showed the highest *in vitro* potency with IC_50_ value of 24 μM (*in silico* ChemScore of 32.9201) and is approximately 28-fold more potent than risedronate (IC_50_ = 660 μM) (Guo et al., 2007). The inhibitory potency of compound **2** (IC_50_ = 25 μM) is considered similar as in compound **3**.

### Binding Site Analysis

**FIGURE 7:**
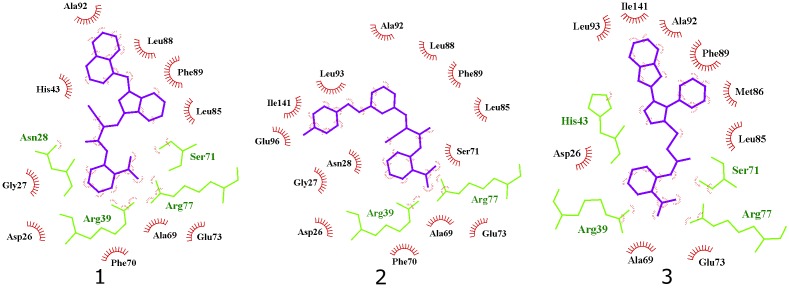
2D projection of calculated binding modes of reported inhibitors **1–3** in their respective UppS protein binding sites 1. Small-molecules are colored blue, amino acid residues forming polar contacts are green. Residues that form lipophilic contacts with small-molecule are presented in black and red and are partially encircled.

Interestingly, the ensamble docking experiment identified anthranilic acid moiety as favorable and compounds were commonly bound to similar protein conformations and binding site volumes, specifically, superimposed protein conformations on the starting crystal complex (PDB ID: 2E98) with backbone RMSD of 1.23 Å, binding site volume 814.625 Å^3^ (compounds **1**, **2**) and 1.39 Å, binding site volume 968.975 Å^3^ for compound **3**. Calculation is in accordance with previous observations and alike bisphosphonates, identified inhibitors bind to the open enzyme form. Similar observations were reported earlier for benzoic acid inhibitors (Figure 6; Zhu et al., 2013) where benzoic acid moiety served as a pyrophosphate mimetic and was connected to a polyaromatic scaffold as mimic of the native substrate (FPP) lipophilic tail (PDB ID: 3SGV; Figure 6). Thus, we postulate the new reported inhibitors (compounds **1**–**3**) could bind to FPP binding site and act as competitive inhibitors. Furthermore, anthranilic acid moiety as a pyrophosphate mimetic has been reported previously, and it has also been conjugated to the lipophilic tail in order to mimic FPP. Wang and coworkers also commented that the electron withdrawing groups on the anthranilic acid moiety improved the potency, while phosphonic acid analogs demonstrated reduced activity (Wang et al., 2016). This reported data can be directly applied for future optimisation of reported inhibitors. Namely, compounds **1**–**3** possess an unsubstituted antranilic acid as a known pyrophosphate mimetic moiety with distinct lipophilic scaffolds to previously published inhibitors. Furthermore, the aforementioned inhibitors by Wang et al. were not evaluated on *E. coli* UppS but Gram positive *Staphylococcus aureus* bacterial strain. Accordingly, to the best of our knowledge, this is the first time this type of compounds are shown to inhibit *E. coli* UppS. The predicted binding mode of inhibitors **1–3** is shown in Figure 7 (Laskowski and Swindells, 2011).

**FIGURE 8:**
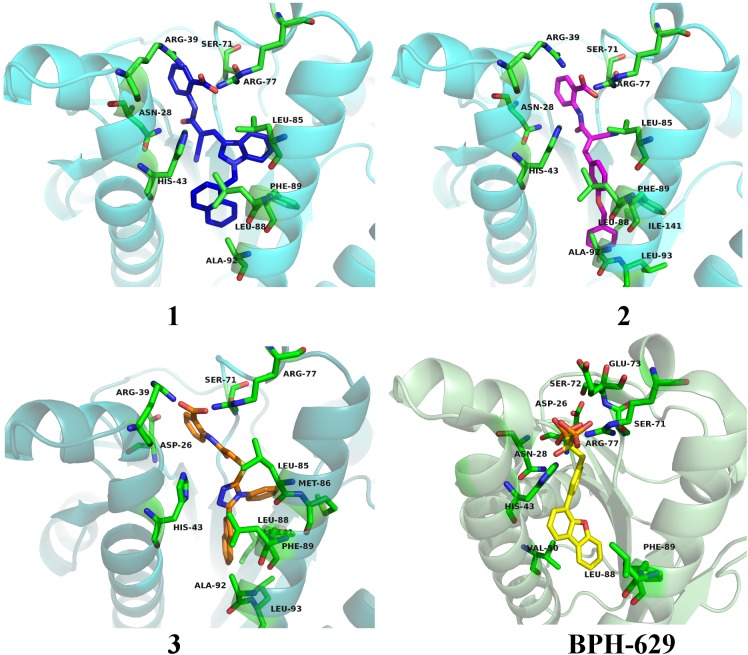
Binding modes of compounds **1–3** (presented in blue, magenta and orange colored stick models, respectively) in UppS active site (PDB ID: 2E98). Co-crystallised ligand **BPH-629** is shown as yellow stick model. Protein is depicted as blue or green colored ribbon model with amino acid residues around ligands presented gren colored stick models.

All three compounds share a similar binding motif where anthranilic acid moiety interacts with phosphate binding pocket (Figure 7). Anthranilic acid carboxylate forms ionic and H-bonds with Arg39 and Arg77 residues that are further stabilized with H-bond toward Ser71. Amide bond connecting anthranilic fragment in all three molecules is positioned in a polar pocket where favorable H-bond interactions with Ala69, Phe70, Ser71 or Met25, Asp26 backbone amides are available. Compounds **1** and **2** therefore form H-bonds with Asn28 or Ser71 through amide or neighbor nitrile functional groups while compound **3** forms a H-bond with His43 via its central triazole moiety. Most potent compound **3** further descends in a voluminous UppS active site gorge where its flexible tioether linker enables effective π-π stacking interaction between Phe89 and phenyltriazole central moiety. Compound **3** additionally makes hydrophobic contacts with Ala47, Val50, Leu85, Met86, Leu88, Phe89, Ala92, Leu93, and Ile141 residues. Compounds **1** and **2** share a similar binding motif, reaching deeper into active site gorge via an acrylonitrile linker moiety. Compounds **1** and **2** therefore make hydrophobic contacts toward Leu85, Leu88, Phe89, and Ala92, while compound **2** additionally interacts with Leu93 and Ile141. Comparatively, co-crystalized bisphosphonate BPH-629 analogously positions its acidic moieties at the top of the gorge making an ionic interaction with Arg77 and H-bonds toward Gly29, Ser72, His43, and Asn28 (Figure 8). It than immediately descends to the lipophilic gorge via a 1,3-subsituted benzene fragment where it forms lipophilic contacts with Met25, His43, Ala47, Val50, Ala69, and Ile141. Branched nature of compounds **1** and **3** can thus effectively account for favorable positioning in a lipophilic active site gorge with additional lipophilic contacts (Leu85, Leu88, Met86, and Phe89). Compound **2** reaches down the active site gorge due to sheer compound length where lipophilic interactions with Ala92 and Leu93 are possible (Figure 8). All three compounds can also be described as spanning to other binding sites (2, 3, Figure 1) and have space for further optimisation.

### Antimicrobial Evaluation

Upon *in vitro* examination, no antibacterial activity was observed for all three inhibitors (**1–3**) when evaluated with wild-type *S. aureus* and *E. coli* bacterial strains so further examination was conducted (Table 1). *E. coli* AcrAB-TolC is a tripartite multidrug efflux pump system that expels compounds form the cell and this represents one of the possible mechanisms of bacterial defence against xenobiotics (Kim et al., 2015). Further microbiological evaluation revealed that lack of antibacterial activity of UPPS inhibitors against *E. coli* can be attributed to their active transport from the bacterial cytoplasm by efflux pumps. Compounds **1** and **3** were inactive against all strains without a permeable membrane (MIC > 32 μg/mL), but showed improved MIC values in efflux deficient *E. coli* BW25113 Δ*acrA* and Δ*acrB* strains in the presence of PMB (MIC = 2 μg/mL in both). On the other hand, compound **2** inhibited bacterial growth in both *E. coli* BW25113 Δ*tolC* strains with or without permeable membrane (MIC = 0.5 μg/mL). The antibacterial activity in the efflux pump-deficient *E. coli* BW25113 Δ*tolC* strain is independent from the presence of polymyxine B. Therefore, it can be postulated on the basis of the evaluation of compound **2**, that transport across *E. coli* cell membrane is possible. This compound therefore represents an interesting starting point for further development, for example computational searches of similar compounds and analog synthesis.

## Conclusion

We have demonstrated a successful implementation of virtual screening techniques in the discovery of *E. coli* UppS inhibitors. With the use of molecular modeling software, we designed a bisphosphonate-based pharmacophore model and used molecular dynamics together with ensemble docking to obtain three novel micromolar UppS inhibitors. These reported anthranilic acid derivatives mimic the structure of polar pyrophosphate and lipophilic moieties of UppS substrates FPP and IPP. Among the 34 top-scoring compounds, the most potent compound **2** displayed inhibitory potency with an IC_50_ value of 25 μM and good antibacterial activity against *E. coli* BW25113 Δ*tolC* with or without a permeable membrane (MIC = 0.5 μg/mL). Our anthranilic acid derivatives **1**–**3** have distinct chemical structures compared to previously known *E. coli* UppS inhibitors, therefore representing a novel starting point for antibacterial drug design targeting UppS.

## Author Contributions

MJ, KR, and SG conceived and designed the experiments. MJ, KR, MS, and HB performed the experiments. MJ, KR, MS, and SG analyzed the data. All co-authors wrote the manuscript.

## Conflict of Interest Statement

The authors declare that the research was conducted in the absence of any commercial or financial relationships that could be construed as a potential conflict of interest.

## Footnotes

1 www.eyesopen.com

2 http://www.rdkit.org/; access September 16, 2018

## Abbreviations

AUC: area under the curve
cpds: compounds
FPP: farnesyl pyrophosphate
Glc*N*Ac-Mur*N*Ac-pentapeptide: *N*-acetylglucosamine-*N*-acetylmuramyl-pentapeptide
IPP: isopentenyl pyrophosphate
MD: molecular dynamics
PMB: polymyxin B
ROC: receiver operating characteristic curve
SAR: structure-activity relationship
UPP: undecaprenyl pyrophosphate
UppS: undecaprenyl pyrophosphate synthase

## References

[B1] Allen M. R. (2018). Recent advances in understanding bisphosphonate effects on bone mechanical properties. *Curr. Osteoporos. Rep.* 16 198–204. 10.1007/s11914-018-0430-3 29497927 PMC6263169

[B2] Apfel C. M. Takacs S. Fountoulakis M. Stieger M. Keck W. (1999). Use of genomics to identify bacterial undecaprenyl pyrophosphate synthetase: cloning, expression, and characterization of the essential uppS gene. *J. Bacteriol.* 181 483–492. 9882662 10.1128/jb.181.2.483-492.1999 PMC93402

[B3] Baell J. B. Holloway G. A. (2010). New substructure filters for removal of pan assay interference compounds (PAINS) from screening libraries and for their exclusion in bioassays. *J. Med. Chem.* 53 2719–2740. 10.1021/jm901137j 20131845

[B4] Chang S. Y. Ko T. P. Chen A. P. C. Wang A. H. J. Liang P. H. (2004). Substrate binding mode and reaction mechanism of undecaprenyl pyrophosphate synthase deduced from crystallographic studies. *Protein Sci.* 13 971–978. 10.1110/ps.03519904 15044730 PMC2280048

[B5] DeLano W. L. (2002). The PyMOL Molecular Graphics System. Available at: http://www.pymol.org

[B6] Fujihashi M. Zhang Y. W. Higuchi Y. Li X. Y. Koyama T. Miki K. (2001). Crystal structure of cis-prenyl chain elongating enzyme, undecaprenyl diphosphate synthase. *Proc. Natl. Acad. Sci. U.S.A.* 98 4337–4342. 10.1073/pnas.071514398 11287651 PMC31836

[B7] Guo R. T. Cao R. Liang P. H. Ko T. P. Chang T. H. Hudock M. P. (2007). Bisphosphonates target multiple sites in both cis- and trans-prenyltransferases. *Proc. Natl. Acad. Sci. U.S.A.* 104 10022–10027. 10.1073/pnas.0702254104 17535895 PMC1877987

[B8] Hornak V. Abel R. Okur A. Strockbine B. Roitberg A. Simmerling C. (2006). Comparison of multiple amber force fields and development of improved protein backbone parameters. *Proteins* 65 712–725. 10.1002/prot.21123 16981200 PMC4805110

[B9] Irwin J. J. Shoichet B. K. (2005). ZINC - A free database of commercially available compounds for virtual screening. *J. Chem. Inf. Model.* 45 177–182. 10.1021/ci049714+ 15667143 PMC1360656

[B10] Jamroz M. Kolinski A. (2013). ClusCo: clustering and comparison of protein models. *BMC Bioinformatics* 14:62. 10.1186/1471-2105-14-62 23433004 PMC3645956

[B11] Jukic M. Rozman K. Gobec S. (2016). Recent advances in the development of undecaprenyl pyrophosphate synthase inhibitors as potential antibacterials. *Curr. Med. Chem.* 23 464–482. 10.2174/0929867323666151231094854 26718796

[B12] Kaye A. D. Cornett E. M. Hart B. Patil S. Pham A. Spalitta M. (2018). Novel pharmacological nonopioid therapies in chronic pain. *Curr. Pain Headache Rep.* 22:31. 10.1007/s11916-018-0674-8 29616344

[B13] Kim J. S. Jeong H. Song S. Kim H. Y. Lee K. Hyun J. (2015). Structure of the tripartite multidrug efflux pump AcrAB-TolC suggests an alternative assembly mode. *Mol. Cells* 38 180–186. 10.14348/molcells.2015.2277 26013259 PMC4332038

[B14] Kim M. O. Feng X. X. Feixas F. Zhu W. Lindert S. Bogue S. (2014). A molecular dynamics investigation of mycobacterium tuberculosis prenyl synthases: conformational flexibility and implications for computer-aided drug discovery. *Chem. Biol. Drug Design* 85 756–769. 10.1111/cbdd.12463 25352216 PMC4412765

[B15] Ko T. P. Chen Y. K. Robinson H. Tsai P. S. Gao Y. G. Chen A. P. C. (2001). Mechanism of product chain length determination and the role of a flexible loop in *Escherichia coli* undecaprenyl-pyrophosphate synthase catalysis. *J. Biol. Chem.* 276 47474–47482. 10.1074/jbc.M106747200 11581264

[B16] Krieger E. Dunbrack R. L. Hooft R. W. Krieger B. (2012). Assignment of protonation states in proteins and ligands: combining pKa prediction with hydrogen bonding network optimization. *Methods Mol. Biol.* 819 405–421. 10.1007/978-1-61779-465-0_25 22183550

[B17] Krieger E. Nielsen J. E. Spronk C. A. Vriend G. (2006). Fast empirical pKa prediction by Ewald summation. *J. Mol. Graph. Model.* 25 481–486. 10.1016/j.jmgm.2006.02.009 16644253

[B18] Krieger E. Vriend G. (2015). New ways to boost molecular dynamics simulations. *J. Comput. Chem.* 36 996–1007. 10.1002/jcc.23899 25824339 PMC6680170

[B19] Kuo C. J. Guo R. T. Lu I. L. Liu H. G. Wu S. Y. Ko T. P. (2008). Structure-based inhibitors exhibit differential activities against *Helicobacter pylori* and *Escherichia coli* undecaprenyl pyrophosphate synthases. *Biomed. Res. Int.* 2008:841312. 10.1155/2008/841312 18382620 PMC2276626

[B20] Laskowski R. A. Swindells M. B. (2011). LigPlot+: multiple ligand-protein interaction diagrams for drug discovery. *J. Chem. Inf. Model.* 51 2778–2786. 10.1021/ci200227u 21919503

[B21] Liang P. H. Ko T. P. Wang A. H. J. (2002). Structure, mechanism and function of prenyltransferases. *Eur. J. Biochem.* 269 3339–3354. 10.1046/j.1432-1033.2002.03014.x 12135472

[B22] Mysinger M. M. Carchia M. Irwin J. J. Shoichet B. K. (2012). Directory of useful decoys, enhanced (DUD-E): better ligands and decoys for better benchmarking. *J. Med. Chem.* 55 6582–6594. 10.1021/jm300687e 22716043 PMC3405771

[B23] Peukert S. Sun Y. C. Zhang R. Hurley B. Sabio M. Shen X. (2008). Design and structure-activity relationships of potent and selective inhibitors of undecaprenyl pyrophosphate synthase (UPPS): tetramic, tetronic acids and dihydropyridin-2-ones. *Bioorg. Med. Chem. Lett.* 18 1840–1844. 10.1016/j.bmcl.2008.02.009 18295483

[B24] Saubern S. Guha R. Baell J. B. (2011). KNIME workflow to assess PAINS filters in SMARTS format. Comparison of RDKit and Indigo cheminformatics libraries. *Mol. Inform.* 30 847–850. 10.1002/minf.201100076 27468104

[B25] Shoichet B. K. (2006). Interpreting steep dose-response curves in early inhibitor discovery. *J. Med. Chem.* 49 7274–7277. 10.1021/jm061103g 17149857

[B26] Sinko W. de Oliveira C. Williams S. Van Wynsberghe A. Durrant J. D. Cao R. (2011). Applying molecular dynamics simulations to identify rarely sampled ligand-bound conformational states of undecaprenyl pyrophosphate synthase, an antibacterial target. *Chem. Biol. Drug Design* 77 412–420. 10.1111/j.1747-0285.2011.01101.x 21294851 PMC3095679

[B27] Sinko W. Wang Y. Zhu W. Zhang Y. Feixas F. Cox C. L. (2014). Undecaprenyl diphosphate synthase inhibitors: antibacterial drug leads. *J. Med. Chem.* 57 5693–5701. 10.1021/jm5004649 24827744 PMC4096218

[B28] Teng K. H. Liang P. H. (2012a). Structures, mechanisms and inhibitors of undecaprenyl diphosphate synthase: a cis-prenyltransferase for bacterial peptidoglycan biosynthesis. *Bioorg. Chem.* 43 51–57. 10.1016/j.bioorg.2011.09.004 21993493

[B29] Teng K. H. Liang P. H. (2012b). Undecaprenyl diphosphate synthase, a cis-prenyltransferase synthesizing lipid carrier for bacterial cell wall biosynthesis. *Mol. Membr. Biol.* 29 267–273. 10.3109/09687688.2012.674162 22471620

[B30] Van Geelen L. Meier D. D. Rehberg N. Kalscheuer R. (2018). Some current concepts in antibacterial drug discovery. *Appl. Microbiol. Biotechnol.* 102 2949–2963. 10.1007/s00253-018-8843-6 29455386

[B31] Wang Y. Desai J. Zhang Y. Malwal S. R. Shin C. J. Feng X. (2016). Bacterial cell growth inhibitors targeting undecaprenyl diphosphate synthase and undecaprenyl diphosphate phosphatase. *Chemmedchem* 11 2311–2319. 10.1002/cmdc.201600342 27578312 PMC5155509

[B32] Wolber G. Langer T. (2005). LigandScout: 3-d pharmacophores derived from protein-bound Ligands and their use as virtual screening filters. *J. Chem. Inf. Model.* 45 160–169. 10.1021/ci049885e 15667141

[B33] Zhu W. Zhang Y. Sinko W. Hensler M. E. Olson J. Molohon K. J. (2013). Antibacterial drug leads targeting isoprenoid biosynthesis. *Proc. Natl. Acad. Sci. U.S.A.* 110 123–128. 10.1073/pnas.1219899110 23248302 PMC3538244

