# Supplementary material for: Anthranilic Acid Inhibitors of Undecaprenyl Pyrophosphate Synthase (UppS), an Essential Enzyme for Bacterial Cell Wall Biosynthesis
Source: Front Microbiol. 2019 Jan 14;9:3322. doi: 10.3389/fmicb.2018.03322 (PMC6339874; doi:10.3389/fmicb.2018.03322)
Supplement: Supplementary file 1 [file Table_1.pdf]

Anthranilic acid inhibitors of undecaprenyl pyrophosphate synthase (UppS), an essential enzyme in bacterial cell wall synthesis

Supplementary data

*Marko Jukič[1], Kaja Rožman[1], Matej Sova[1], Hélène Barreteau[2], Stanislav Gobec[1]**

[1]Faculty of Pharmacy, University of Ljubljana, Aškerčeva 7, SI-1000 Ljubljana, Slovenia

[2]Bacterial Cell Envelopes and Antibiotics Group, Institute for Integrative Biology of the Cell (I2BC), CEA, CNRS, University of Paris Sud, Université Paris-Saclay, 91198 Gif-sur-Yvette cedex, France

*CORRESPONDENCE: Stanislav Gobec, E-mail: stanislav.gobec@ffa.uni-lj.si, Phone +38614769585.

Computer Hardware

The compound database preparation, MD and all of the computations were carried out on a four eight-core AMD Opteron 6128 Magny-Cours workstation with 32 GB RAM, two 1 TB HDD, running 64-bit Ubuntu Linux 16.04.2. The binding-site preparation and preparation of images was conducted on a two quad-core Intel Xeon 2.2 GHz workstation with 16 GB RAM, 1 TB HDD, 1 TB SSD and an Nvidia GTX 1050 graphic card, running 64-bit Debian Linux and on a workstation with 8-core Intel i7 Haswell 4710, 2.5 GHz processors, with 8 GB RAM and one 1 TB hard drive (WD), while running 64-bit Microsoft Windows 8.1 operating system.

Pharmacophore model design

The pharmacophore model was designed using LigandScout program based on the known UppS inhibitor **1** (**BPH-629**), while taking in consideration the known interactions of the bisphosphonate with the enzyme (PDB ID: 2E98).[1] Specific fragments or functional groups of the inhibitor were used to pinpoint the desired type of interactions with the enzyme and thus formed the final pharmacophore model. More specifically: Negative ionisable feature at position 39.97, 44.00, 56,39 measuring 2.2 Å in diameter and weight 1; H-bond acceptor feature at position 37.35, 42.49, 56.48 measuring 2.35 Å in diameter and weight 1. Additionally, aliphatic hydroxyl group was marked as hydrophilic H-bond donor feature (position: 39.57, 41.81, 57.82; 1.6 Å in diameter; weight 1) and distant aromatic ring as a hydrophobic feature of the pharmacophore (2.4 Å in diameter; position: 43.43, 32.83, 54.88; weight 1). Finally, the exclusion zones predicted by the LigandScout 4.2 program[1] have been included in the final pharmacophore model. Such exclusion zones arise from the protein amino acid residues or water molecules bound in the crystal during the crystallization.

Exclusion zone list:

</point><volume type="exclusion" featureId="1763848723011623622" optional="false" disabled="false" weight="1.0" id="feature4"><position x3="40.3246" y3="37.3536" z3="52.3292" tolerance="1.5" /></volume><volume type="exclusion" featureId="1763848614627643268" optional="false" disabled="false" weight="1.0" id="feature5"><position x3="38.889" y3="33.75" z3="53.768" tolerance="1.5" /></volume><volume type="exclusion" featureId="1763848719978313618" optional="false" disabled="false" weight="1.0" id="feature6"><position x3="37.471" y3="38.709" z3="58.208" tolerance="1.0" /></volume><volume type="exclusion" featureId="1773405988438621594 97" optional="false" disabled="false" weight="1.0" id="feature7"><position x3="44.162" y3="35.428" z3="51.483944" tolerance="1.5" /></volume><volume type="exclusion" featureId="1773406002179761595 01" optional="false" disabled="false" weight="1.0" id="feature8"><position x3="47.162" y3="35.428" z3="54.483944" tolerance="1.5" /></volume><volume type="exclusion" featureId="1763848656649313616" optional="false" disabled="false" weight="1.0" id="feature9"><position x3="42.446" y3="42.77" z3="58.672" tolerance="1.0" /></volume><volume type="exclusion" featureId="1763848819838215000" optional="false" disabled="false" weight="1.0" id="feature10"><position x3="34.759" y3="35.755" z3="53.94" tolerance="1.0" /></volume><volume

type="exclusion" featureId="1763848609755523215" optional="false" disabled="false" weight="1.0" id="feature11"><position x3="40.6928" y3="30.906334" z3="57.625645" tolerance="1.5" /></volume><volume type="exclusion" featureId="1763848821984915003" optional="false" disabled="false" weight="1.0" id="feature12"><position x3="42.218" y3="39.636" z3="61.745" tolerance="1.0" /></volume><volume type="exclusion" featureId="1763848825360335004" optional="false" disabled="false" weight="1.0" id="feature13"><position x3="37.041" y3="40.224" z3="50.753" tolerance="1.0" /></volume><volume type="exclusion" featureId="1773406002875371595O2" optional="false" disabled="false" weight="1.0" id="feature14"><position x3="47.162" y3="36.928" z3="58.983944" tolerance="1.5" /></volume><volume type="exclusion" featureId="1763848748977254480" optional="false" disabled="false" weight="1.0" id="feature15"><position x3="43.4544" y3="33.8126" z3="61.625" tolerance="1.5" /></volume><volume type="exclusion" featureId="1763848825853535005" optional="false" disabled="false" weight="1.0" id="feature16"><position x3="38.629" y3="43.416" z3="51.996" tolerance="1.0" /></volume><volume type="exclusion" featureId="1763848820382735001" optional="false" disabled="false" weight="1.0" id="feature17"><position x3="33.873" y3="39.248" z3="55.195" tolerance="1.0" /></volume><volume type="exclusion" featureId="1773405971381931594 94" optional="false" disabled="false" weight="1.0" id="feature18"><position x3="41.162" y3="33.928" z3="48.483944" tolerance="1.5" /></volume><volume type="exclusion" featureId="1763848720414493620" optional="false" disabled="false" weight="1.0" id="feature19"><position x3="35.377" y3="40.898" z3="59.361" tolerance="1.0" /></volume><volume type="exclusion" featureId="1763848775726864999" optional="false" disabled="false" weight="1.0" id="feature20"><position x3="40.518" y3="44.797" z3="52.6" tolerance="1.0" /></volume><volume type="exclusion" featureId="1773406001469901595 00" optional="false" disabled="false" weight="1.0" id="feature21"><position x3="47.162" y3="32.428" z3="58.983944" tolerance="1.5" /></volume><volume type="exclusion" featureId="17734059509270115949 2" optional="false" disabled="false" weight="1.0" id="feature22"><position x3="38.162" y3="29.428" z3="52.983944" tolerance="1.5" /></volume><volume type="exclusion" featureId="1763848613684013242" optional="false" disabled="false" weight="1.0" id="feature23"><position x3="44.365" y3="31.098" z3="50.167202" tolerance="1.5" /></volume><volume type="exclusion" featureId="1763848572557513209" optional="false" disabled="false" weight="1.0" id="feature24"><position x3="36.673" y3="43.301" z3="60.261" tolerance="1.0" /></volume><volume type="exclusion" featureId="1763848595726443212" optional="false" disabled="false" weight="1.0" id="feature25"><position x3="42.937" y3="45.884" z3="54.159" tolerance="1.0" /></volume><volume type="exclusion" featureId="1763848739173144461" optional="false" disabled="false" weight="1.0" id="feature26"><position x3="37.15" y3="45.041" z3="52.932" tolerance="1.0" /></volume><volume type="exclusion" featureId="1763848596128413214" optional="false" disabled="false" weight="1.0" id="feature27"><position x3="43.218" y3="46.153" z3="56.446" tolerance="1.0" /></volume><volume type="exclusion" featureId="1763848820602245002" optional="false" disabled="false" weight="1.0" id="feature28"><position x3="33.126" y3="41.108" z3="58.4" tolerance="1.0" /></volume><volume type="exclusion" featureId="1763848613578533241" optional="false" disabled="false" weight="1.0" id="feature29"><position x3="45.046" y3="29.32" z3="52.184" tolerance="1.5" /></volume><volume type="exclusion" featureId="1773405986919111594 96" optional="false" disabled="false" weight="1.0" id="feature30"><position x3="44.162" y3="29.428" z3="60.483944" tolerance="1.5" /></volume><volume type="exclusion" featureId="1773406007416791595 03" optional="false" disabled="false" weight="1.0" id="feature31"><position x3="48.662" y3="32.428" z3="51.483944" tolerance="1.5" /></volume><volume type="exclusion" featureId="1773406000081531594 99" optional="false" disabled="false" weight="1.0" id="feature32"><position x3="47.162" y3="29.428" z3="55.983944" tolerance="1.5" /></volume><volume type="exclusion" featureId="1763848738816794459" optional="false" disabled="false" weight="1.0" id="feature33"><position x3="34.662" y3="44.771" z3="54.241" tolerance="1.0" /></volume><volume type="exclusion" featureId="1773406013292411595 04" optional="false" disabled="false" weight="1.0" id="feature34"><position x3="50.162" y3="32.428" z3="55.983944" tolerance="1.5" /></volume><volume type="exclusion" featureId="1763848775093974997" optional="false" disabled="false" weight="1.0" id="feature35"><position x3="38.44" y3="47.164" z3="53.875" tolerance="1.0" /></volume><volume type="exclusion" featureId="1773405968197521594 93" optional="false" disabled="false" weight="1.0" id="feature36"><position x3="41.162" y3="26.428" z3="54.483944" tolerance="1.5" /></volume><volume type="exclusion" featureId="1773405986160781594 95" optional="false" disabled="false" weight="1.0" id="feature37"><position x3="44.162" y3="26.428" z3="57.483944" tolerance="1.5"

Compound Database Preparation and Ligand-based Screening

For the virtual screening, the `ZINC Drugs Now´ subset with 10.7 million drug-like compounds was used.[2] Prior to the docking, hierarchical filtering of the compound database was performed with the aim to remove unwanted compounds. The `ZINC Drugs Now´ subset was first processed with the FILTER 2.5.1.4 application [3] to eliminate the known or predicted

aggregators[4] and the compounds with predicted poor solubility. The following filter configuration file was applied:

ELIMINATE METALS

MIN_MOLWT 300
MAX_MOLWT 1000
MIN_ROT_BONDS 0
MAX_ROT_BONDS 15
MIN_RIGID_BONDS 4
MAX_RIGID_BONDS 55
MIN_XLOG_P -4.0
MAX_XLOG_P 6.85
MIN_SOLUBILITY poorly "Minimum solubility"
AGGREGATORS true "Eliminate known aggregators"
PRED_AGG true "Eliminate predicted aggregators"

The library was further processed with an in-house Python scripts capable of removing the PAINS compounds and filtered for REOS structures.[5] The resulting filtered library consisted of roughly 6.5 million compounds. Next, the 3D conformer database was prepared with Openeye (OpenEye Scientific Software, Inc., Santa Fe, NM, USA; www.eye-sopen.com) omega2 fast protocol within LigandScout. Maximum number of conformations was set at 25, rms threshold of 0.8 was used and energy window of 10 with 4,000 max pool size with ring and nitrogen enumeration. Final LigandScout ldb database was created using idbgen software from Inte:Ligand (Software- Entwicklungs und Consulting GmbH, Maria Enzersdorf, Austria). Prepared conformer library was filtered further using a newly designed pharmacophore model based on the bisphosphonate inhibitor **BPH-629** (detailed description in the previous subsection) with screening protocol within LigandScout[1] (iscreen software; Software- Entwicklungs und Consulting GmbH, Maria Enzersdorf, Austria). Used parameters were: Pharmacophore-Fit scoring function and match all query features screening mode. Retrieval mode was: Stop after first matching conformation. Max number of omitted features was 1 and exclusion spheres were employed in the calculation. Thus the final enriched library used for the structure-based virtual screening consisted of 13 530 compounds and was used for the structure-based virtual screening.

Structure-Based Virtual Screening – compound list [6, 7]

Table S1: List of top-scoring compounds in structure-based virtual screening model.

Compound	Vendors code	Chemical structure	GOLD.ChemScore. Fitness	Molecular weight (g/mol)	% of inhibition	MIC (µg/mL)	
						E. coli	*S. aureus*
**S1** (main text: **1**)	**STK093750**	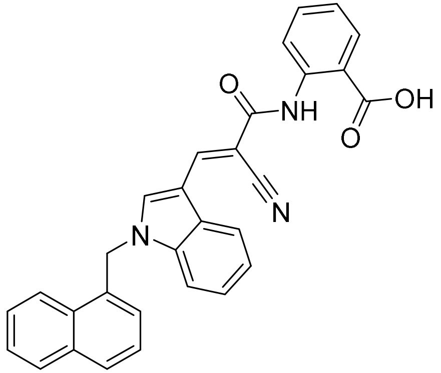	41.8266	471.52	IC_{50} = 45 µM	> 128	> 128
**S2**	**AG-205/05893006**	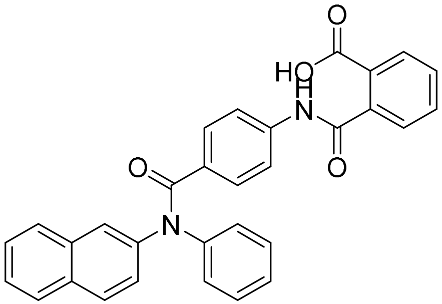	38.3248	486.53	58	> 128	> 128

**S3**	**AQ-360/42570543**	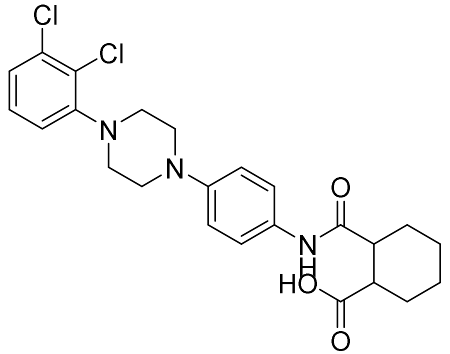	37.6963	476.40	96	> 128	> 128
**S4**	**IBS-L0207254**	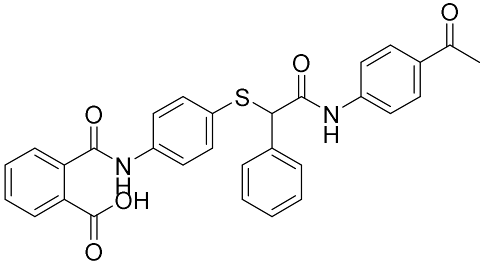	35.7491	524.59	101	> 128	> 128
**S5**	**STL157126**	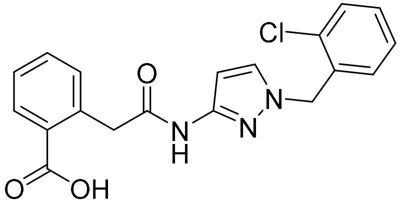	34.9051	369.81	115	> 128	> 128
**S6**	**AQ-360/42570550**	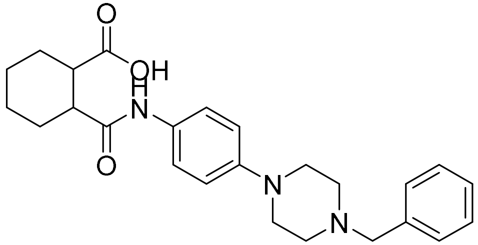	34.8710	421.54	53	> 128	> 128

**S7**	**AG-690/33916006**	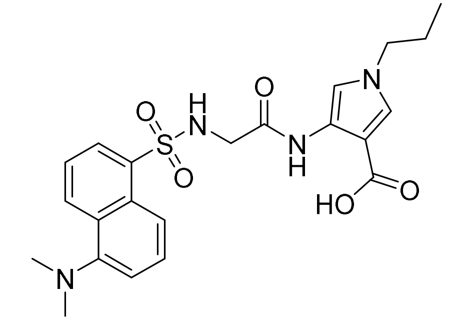	33.5441	458.53	85	> 128	> 128
**S8**	**PB56864753**	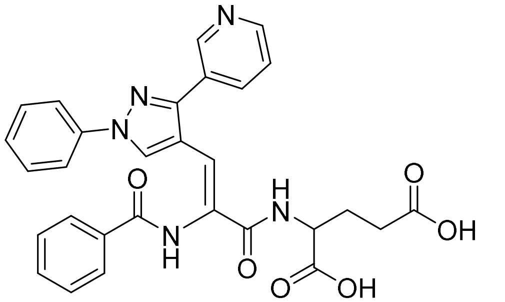	33.3548	539.55	92	> 128	> 128
**S9**	**7126272**	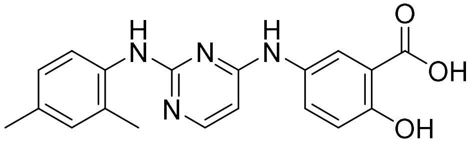	33.1160	350.38	72	> 128	> 128
**S10** (main text: **3**)	**STL365344**	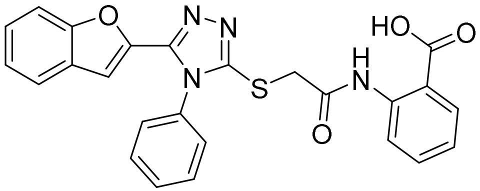	32.9201	470.50	IC_{50} = 24 μM	> 128	> 128

**S11**	**AG-690/33098016**	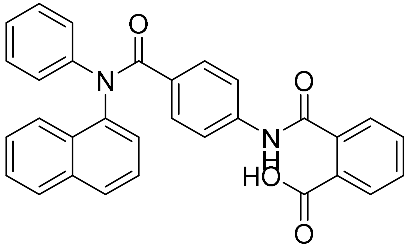	32.8162	486.53	60	> 128	> 128
**S12**	**AG-205/34698038**	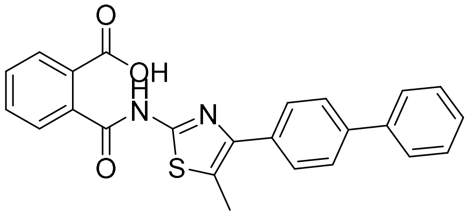	32.5918	414.48	120	> 128	> 128
**S13**	**Z55988003**	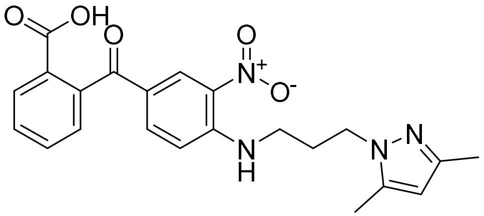	30.3264	422.44	92	> 128	> 128
**S14**	**STK043985**	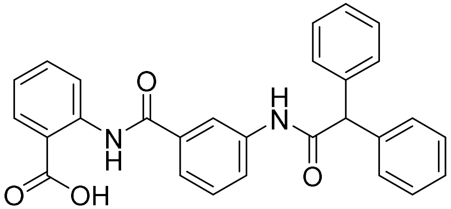	30.1626	450.49	101	> 128	128
**S15**	**Z2242878632**	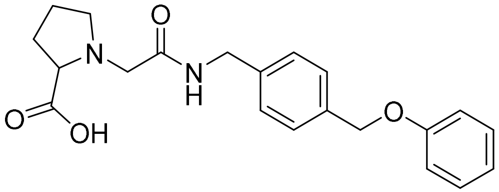	30.0135	368.43	124	> 128	> 128

**S16**	**AQ-360/42570784**	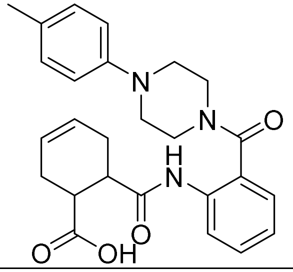	29.1905	447.54	104	> 128	> 128
**S17**	**Z1445398572**	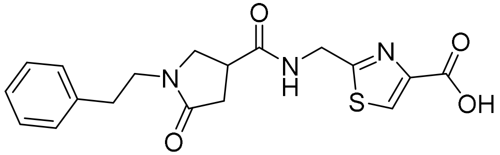	28.5765	373.43	70	> 128	> 128
**S18**	**J047-0617**	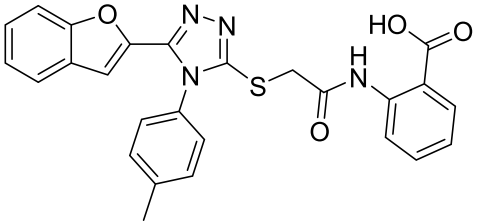	28.1411	484.53	122	> 128	> 128
**S19**	**AQ-360/42570789**	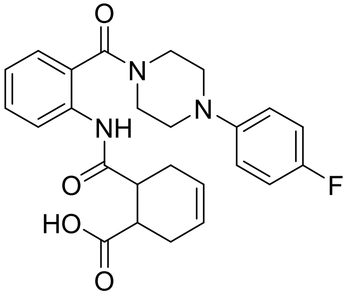	28.0565	451.50	99	> 128	> 128
**S20**	**Z1537404453**	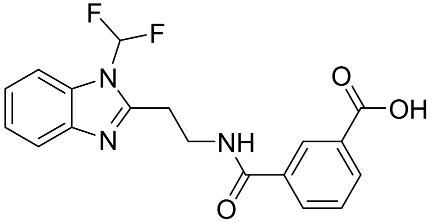	27.1972	359.33	168	> 128	> 128

**S21**	**F687-0934**	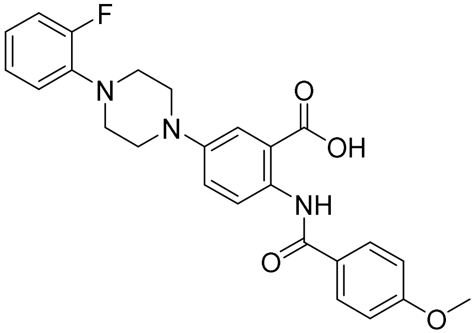	26.2106	449.48	74	> 128	> 128
**S22**	**Z1444164038**	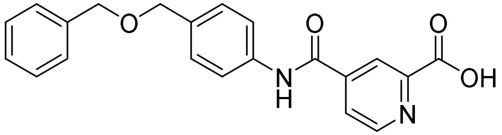	25.2857	362.39	93	> 128	> 128
**S23**	**IBS-L0125921**	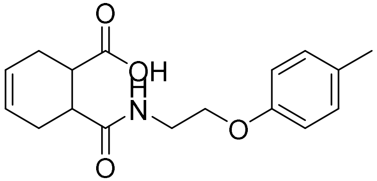	24.0976	303.36	117	> 128	> 128
**S24** (main text: **2**)	**STK113564**	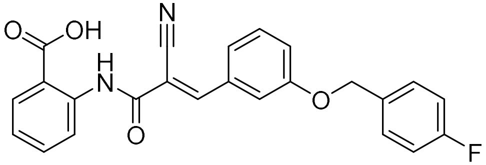	23.9449	416.41	IC_{50} = 25 μM	> 128	> 128
**S25**	**F687-0999**	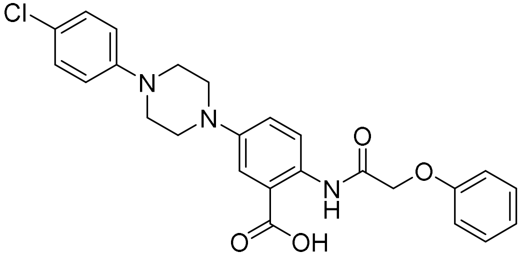	23.7389	465.93	121	> 128	64

**S26**	**STL123826**	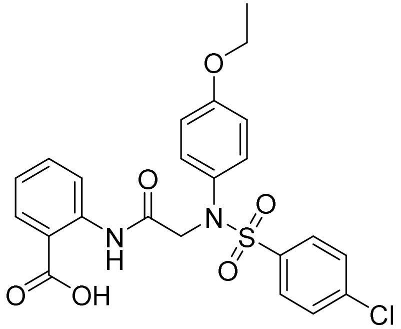	23.3255	488.94	102	> 128	128
**S27**	**STK541118**	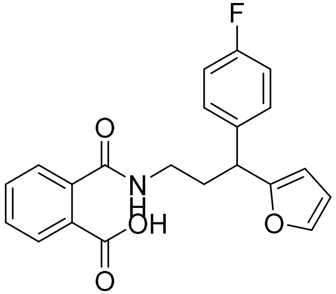	23.0163	367.38	99	> 128	> 128
**S28**	**F684-0517**	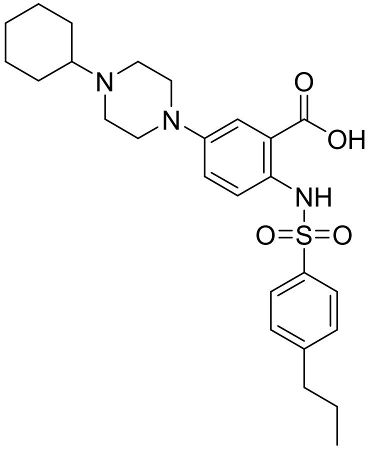	22.8636	485.64	113	> 128	> 128

**S29**	**STL425508**	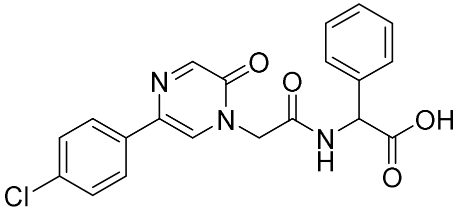	22.7323	397.82	96	> 128	> 128
**S30**	**STL249593**	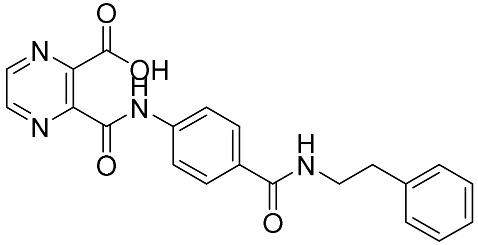	21.4719	390.40	122	> 128	> 128
**S31**	**F687-0385**	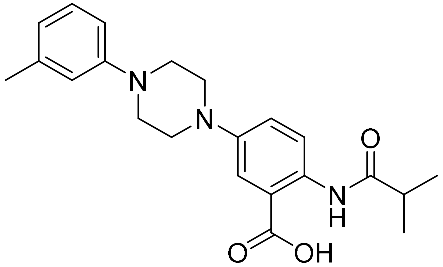	21.4464	381.48	125	> 128	> 128
**S32**	**STK292488**	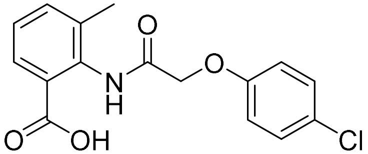	21.3059	319.74	122	> 128	> 128

**S33**	**STK068080**	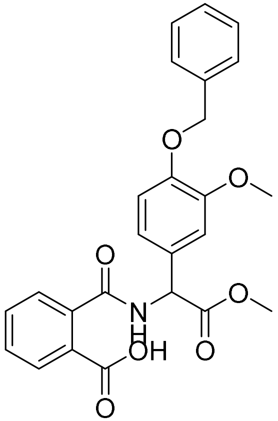	20.7217	449.46	65	> 128	> 128
**S34**	**STK525524**	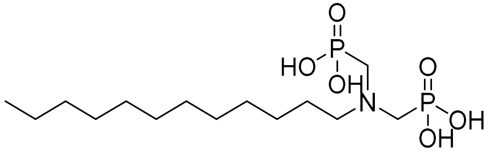	15.2135	373.37	147	> 128	128
**risedronate**	/	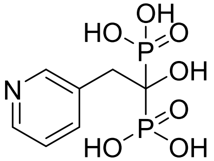	/	283.11	/	/	/

Compound characterization

Compounds were purchased from several vendors (compounds **1** and **2** from Alinda Chemical, Ltd., compounds **3**-**7** from ChemDiv Inc., compounds **8**-**12** from Enamine Ltd., compound **13** from Otava Ltd., compounds **14**-**21** from Specs, compound **22** from UkrOrgSynthesis Ltd., compounds **23**-**34** from Vitas-M and risedronate from Sigma-Aldrich) and were evaluated without further purification. Additionally, DMSO, NaCl, KCl, $MgCl_2$, FPP and HEPES buffer were obtained from commercial sources (Sigma-Aldrich, Merck Millipore) and were used without further purification. The quantitative evaluation of UPP enzymatic production was determined on an optical radioactivity reader Rita Star (Raytest Isotopenmessgeräte GmbH, Straubenhardt, Germany). ^{1}H NMR spectra were recorded in DMSO-d_6 on a Bruker Avance 400 DPX 400 MHz spectrophotometer at 298 K. Chemical shifts are reported in ppm downfield from tetramethylsilane or solvents. The coupling constants (*J*) are in Hz, and the splitting patterns are designated as: s, singlet; br s, broad singlet; ds, doublet of singlet; d, doublet; dd, double doublet; ddd, triple doublet; t, triplet; dt, double triplet; and m, multiplet. ESI-HRMS mass spectra were recorded on Thermo Scientific™ Exactive™ Plus Orbitrap Mass Spectrometer at Faculty of Pharmacy in Ljubljana. The purity of purchased compounds was determined on a Thermo Scientific Dionex UltiMate 3000 HPLC system using an Agilent Extend-C18 3.5 μm column (4.6 × 150 mm), with a flow rate of 1.0 mL/min, injection volume 5 μL, and detection at several wavelength (210, 220, 254 and 280 nm). The following gradient was applied: 40 to 90% CH_3CN/0.1% TFA in 12 min, then 2 min at 90% CH_3CN/0.1% TFA and 1 min from 90 to 40% CH_3CN/0.1% TFA.

2-(2-((5-(benzofuran-2-yl)-4-phenyl-4H-1,2,4-triazol-3-yl)thio)acetamido)benzoic acid (**25**): White solid; ^{1}H NMR (400 MHz, $CDCl_3$, δ) ppm 4.36 (s, 2H, CH_2), 6.50 (s, 1H, Ar-H), 7.02 (s, 1H, Ar-H), 7.10-7.17 (m, 1H, Ar-H), 7.21-7.27 (m, 2H, Ar-H), 7.30-7.36 (m, 2H, Ar-H), 7.40-7.50 (m, 2H, Ar-H), 7.54 (s, 1H, Ar-H), 7.60-7.71 (m, 2H, Ar-H), 8.10-8.15 (m, 1H, Ar-H), 8.67-8.69 (m, 1H, Ar-H), 11.83 (s, 1H, NH), COOH is exchanged; ESI-HRMS ([M+H$^+$], (m/z)) Calculated for $C_{25}H_{18}N_4O_4S$: 471.1115, Found 471.1121; HPLC: t_R = 9.407 min (98 %).

(E)-2-(2-cyano-3-(1-(naphthalen-1-ylmethyl)-1H-indol-3-yl)acrylamido)benzoic acid (**29**): Yellow solid; ^{1}H NMR (400 MHz, $CDCl_3$, δ) ppm 4.79-4.84 (m, 1H, Ar-H), 5.64-5.69 (m, 1H, Ar-H), 5.76-5.81 (m, 1H, Ar-H), 6.12-6.18 (m, 2H, CH_2), 6.67-6.71 (m, 1H, Ar-H), 6.79-

6.88 (m, 2H, Ar-H), 6.90-7.00 (m, 3H, Ar-H), 7.18-7.22 (m, 1H, Ar-H), 7.37-7.39 (m, 1H, Ar-H), 7.43-7.51 (m, 3H, Ar-H and CH), 7.59-7.61 (m, 1H, Ar-H), 8.33-8.36 (m, 1H, Ar-H), 8.50 (s, 1H, Ar-H), 11.10 (s, 1H, NH), COOH is exchanged; ESI-HRMS ([M-H^+], (m/z)) Calculated for $C_{30}H_{21}N_3O_3$: 470.1499, Found 470.1512; HPLC: t_R = 12.937 min (95 %).

(E)-2-(2-cyano-3-(3-((4-fluorobenzyl)oxy)phenyl)acrylamido)benzoic acid (**31**): White solid; ^{1}H NMR (400 MHz, methanol-*d₄*, δ) ppm 5.16 (s, 2H, CH_2), 7.10-7.15 (m, 2H, Ar-H), 7.17-7.24 (m, 2H, Ar-H), 7.45-7.53 (m, 4H, Ar-H), 7.60-7.83 (m, 1H, Ar-H), 7.74-7.75 (m, 1H, Ar-H), 8.12-8.14 (m, 1H, Ar-H), 8.33 (s, 1H, CH), 8.63-8.65 (m, 1H, Ar-H), COOH and amide NH are exchanged; ESI-HRMS ([M-H^+], (m/z)) Calculated for $C_{24}H_{17}FN_2O_4$: 415.1089, Found 415.1102; HPLC: t_R = 11.910 min (97 %).

MD trajectory clustering with ClusCo [8]

10 clusters formation and centroid definition (flagged by a star in the output log) as calculated by ClusCo.

Score: rmsd

Filename: filelist.txt (conformers: 201)

Hierarchical clustering, pairwise average-linkage, K=10

0 : model_140.pdb : 0.478 : 1.1 : model_100.pdb model_101.pdb model_102.pdb model_104.pdb model_105.pdb model_106.pdb model_107.pdb model_109.pdb model_110.pdb model_113.pdb model_114.pdb model_115.pdb model_116.pdb model_117.pdb model_118.pdb model_119.pdb model_122.pdb model_123.pdb model_124.pdb model_126.pdb model_127.pdb model_128.pdb model_129.pdb model_130.pdb model_131.pdb model_132.pdb model_134.pdb model_135.pdb model_136.pdb model_137.pdb model_138.pdb model_139.pdb model_140.pdb model_141.pdb model_143.pdb model_144.pdb model_146.pdb model_147.pdb model_148.pdb model_149.pdb model_150.pdb model_152.pdb model_153.pdb model_157.pdb model_158.pdb model_159.pdb model_160.pdb model_161.pdb model_162.pdb model_163.pdb model_164.pdb model_165.pdb model_166.pdb model_167.pdb model_170.pdb model_172.pdb model_173.pdb model_175.pdb model_176.pdb model_178.pdb model_181.pdb model_182.pdb model_183.pdb model_185.pdb model_188.pdb model_189.pdb model_190.pdb model_194.pdb model_196.pdb model_197.pdb model_198.pdb model_199.pdb model_200.pdb model_201.pdb model_68.pdb model_69.pdb model_70.pdb model_71.pdb model_72.pdb model_74.pdb model_77.pdb model_82.pdb model_83.pdb model_84.pdb model_85.pdb model_86.pdb model_87.pdb model_89.pdb model_90.pdb model_91.pdb model_92.pdb model_95.pdb model_96.pdb model_97.pdb model_98.pdb model_99.pdb

1 : model_41.pdb : 0.204 : 1.2 : model_10.pdb model_11.pdb model_12.pdb model_13.pdb model_14.pdb model_15.pdb model_16.pdb model_17.pdb model_18.pdb model_19.pdb model_20.pdb model_21.pdb model_22.pdb model_23.pdb model_24.pdb model_25.pdb model_28.pdb model_29.pdb model_30.pdb model_31.pdb model_32.pdb model_33.pdb model_34.pdb model_35.pdb model_36.pdb model_37.pdb model_38.pdb model_39.pdb model_40.pdb model_41.pdb model_42.pdb model_43.pdb model_44.pdb model_61.pdb model_64.pdb model_65.pdb model_6.pdb model_78.pdb model_7.pdb model_8.pdb model_9.pdb

2 : model_80.pdb : 0.164 : 1.1 : model_103.pdb model_108.pdb model_111.pdb model_112.pdb model_142.pdb model_151.pdb model_154.pdb model_155.pdb model_156.pdb model_168.pdb model_169.pdb model_46.pdb model_47.pdb model_48.pdb model_49.pdb model_50.pdb model_51.pdb model_53.pdb model_56.pdb model_57.pdb model_58.pdb model_59.pdb model_60.pdb model_62.pdb model_63.pdb

model_73.pdb model_75.pdb model_76.pdb model_79.pdb model_80.pdb model_81.pdb model_93.pdb model_94.pdb

3 : model_133.pdb : 0.0746 : 1.2 : model_121.pdb model_125.pdb model_133.pdb model_145.pdb model_171.pdb model_174.pdb model_177.pdb model_179.pdb model_180.pdb model_184.pdb model_187.pdb model_191.pdb model_193.pdb model_195.pdb model_88.pdb

4 : model_52.pdb : 0.0348 : 1.1 : model_26.pdb model_27.pdb model_45.pdb model_52.pdb model_55.pdb model_66.pdb model_67.pdb

5 : model_5.pdb : 0.0199 : 0.95 : model_1.pdb model_3.pdb model_4.pdb model_5.pdb

6 : model_192.pdb : 0.00995 : 1.1 : model_120.pdb model_192.pdb

7 : model_2.pdb : 0.00498 : 0 : model_2.pdb

8 : model_186.pdb : 0.00498 : 0 : model_186.pdb

9 : model_54.pdb : 0.00498 : 0 : model_54.pdb

Score: rmsd

Filename: filelist.txt (conformers: 201)

Hierarchical clustering, pairwise average-linkage, K=10

model_100.pdb 5

model_101.pdb 5

model_102.pdb 5

model_103.pdb 6

model_104.pdb 5

model_105.pdb 5

model_106.pdb 5

model_107.pdb 5

model_108.pdb 6

model_109.pdb 5

model_10.pdb 3

model_110.pdb 5

model_111.pdb 6

model_112.pdb 6

model_113.pdb 5

model_114.pdb 5

model_115.pdb 5

model_116.pdb 5

model_117.pdb 5

model_118.pdb 5

model_119.pdb 5

model_11.pdb 3

model_120.pdb 8

model_121.pdb 4

model_122.pdb 5

model_123.pdb 5

model_124.pdb 5

model_125.pdb 4

model_126.pdb 5

model_127.pdb 5

model_128.pdb 5

model_129.pdb 5

model_12.pdb 3

model_130.pdb 5

model_131.pdb 5

model_132.pdb 5

model_133.pdb 4 *

model_134.pdb 5

model_135.pdb 5

model_136.pdb 5

model_137.pdb 5

model_138.pdb 5

model_139.pdb 5

model_13.pdb 3

model_140.pdb 5 *

model_141.pdb 5

model_142.pdb 6

model_143.pdb 5

model_144.pdb 5

model_145.pdb 4

model_146.pdb	5
model_147.pdb	5
model_148.pdb	5
model_149.pdb	5
model_14.pdb	3
model_150.pdb	5
model_151.pdb	6
model_152.pdb	5
model_153.pdb	5
model_154.pdb	6
model_155.pdb	6
model_156.pdb	6
model_157.pdb	5
model_158.pdb	5
model_159.pdb	5
model_15.pdb	3
model_160.pdb	5
model_161.pdb	5
model_162.pdb	5
model_163.pdb	5
model_164.pdb	5
model_165.pdb	5
model_166.pdb	5
model_167.pdb	5
model_168.pdb	6
model_169.pdb	6
model_16.pdb	3
model_170.pdb	5
model_171.pdb	4
model_172.pdb	5
model_173.pdb	5
model_174.pdb	4
model_175.pdb	5

model_176.pdb 5

model_177.pdb 4

model_178.pdb 5

model_179.pdb 4

model_17.pdb 3

model_180.pdb 4

model_181.pdb 5

model_182.pdb 5

model_183.pdb 5

model_184.pdb 4

model_185.pdb 5

model_186.pdb 2 *

model_187.pdb 4

model_188.pdb 5

model_189.pdb 5

model_18.pdb 3

model_190.pdb 5

model_191.pdb 4

model_192.pdb 8 *

model_193.pdb 4

model_194.pdb 5

model_195.pdb 4

model_196.pdb 5

model_197.pdb 5

model_198.pdb 5

model_199.pdb 5

model_19.pdb 3

model_1.pdb 9

model_200.pdb 5

model_201.pdb 5

model_20.pdb 3

model_21.pdb 3

model_22.pdb 3

model_23.pdb	3
model_24.pdb	3
model_25.pdb	3
model_26.pdb	7
model_27.pdb	7
model_28.pdb	3
model_29.pdb	3
model_2.pdb	1 *
model_30.pdb	3
model_31.pdb	3
model_32.pdb	3
model_33.pdb	3
model_34.pdb	3
model_35.pdb	3
model_36.pdb	3
model_37.pdb	3
model_38.pdb	3
model_39.pdb	3
model_3.pdb	9
model_40.pdb	3
model_41.pdb	3 *
model_42.pdb	3
model_43.pdb	3
model_44.pdb	3
model_45.pdb	7
model_46.pdb	6
model_47.pdb	6
model_48.pdb	6
model_49.pdb	6
model_4.pdb	9
model_50.pdb	6
model_51.pdb	6
model_52.pdb	7 *

model_53.pdb	6
model_54.pdb	0 *
model_55.pdb	7
model_56.pdb	6
model_57.pdb	6
model_58.pdb	6
model_59.pdb	6
model_5.pdb	9 *
model_60.pdb	6
model_61.pdb	3
model_62.pdb	6
model_63.pdb	6
model_64.pdb	3
model_65.pdb	3
model_66.pdb	7
model_67.pdb	7
model_68.pdb	5
model_69.pdb	5
model_6.pdb	3
model_70.pdb	5
model_71.pdb	5
model_72.pdb	5
model_73.pdb	6
model_74.pdb	5
model_75.pdb	6
model_76.pdb	6
model_77.pdb	5
model_78.pdb	3
model_79.pdb	6
model_7.pdb	3
model_80.pdb	6 *
model_81.pdb	6
model_82.pdb	5

model_83.pdb	5
model_84.pdb	5
model_85.pdb	5
model_86.pdb	5
model_87.pdb	5
model_88.pdb	4
model_89.pdb	5
model_8.pdb	3
model_90.pdb	5
model_91.pdb	5
model_92.pdb	5
model_93.pdb	6
model_94.pdb	6
model_95.pdb	5
model_96.pdb	5
model_97.pdb	5
model_98.pdb	5
model_99.pdb	5
model_9.pdb	3

Binding site examination [9]

All 10 cluster centroids identified with ClusCo using MD trajectory data were superimposed on the starting crystalized protein conformation (PDB ID: 2E98) and the binding site was defined as an area of 7 Å around the co-crystallized ligand BPH-629 in binding site 1. SiteMap software from Schrödinger (Schrödinger Release 2018-3: SiteMap, Schrödinger, LLC, New York, NY, 2018) was used to calculate binding site areas and surfaces. [9, 10] Used parameters were 15 site points per reported site (minimum site size), more restrictive definition of hydrophobicity (eliminating points that are adjacent to hydrophilic regions and assigning reduced hydrophobicity to solvent-exposed regions) and standard grid resolution (0.7 Å). Data for the reference crystal structure is as follows: binding site surface (Å^2): accptr-807.244; donor-840.011; phil-1581.452; phob-303.806; surf-2847.953 and binding site volume of 853.727 Å^3. Data for selected cluster centroids is presented in Table S2.

Table S2: Binding site surface areas and binding site volumes of clustered protein centroid conformations

	Surface Type and corresponding area (Å^2)					Binding site volume (Å^3)
centroid conformation no.	*acceptor*	*donor*	*hydrophillic*	*hydrophobic*	*surface*	
1	919.127	1119.107	1961.304	279.929	3362.488	862.645
2	855.685	910.592	1707.388	459.005	3197.214	789.929
3	766.771	890.057	1686.257	316.344	3204.337	814.625
4	821.101	820.326	1642.737	320.038	3262.737	894.887
5	979.57	840.066	1811.742	399.324	3487.816	1002.932
6	978.3	1010.715	1993.606	386.09	3862.864	932.960
7	1063.121	1132.784	2166.03	412.147	4002.275	1001.903
8	796.925	819.21	1592.704	435.629	3180.268	968.975
9	778.257	883.509	1644.696	398.738	3105.713	890.771
10	877.396	921.634	1801.628	385.566	3303.367	895.573

PAINS filtering (definitions of PAINS compounds in SMARTS format) [11]

1. c:1:c:c(:c:c:c:1-[#6;X4]-c:2:c:c:c(:c:c:2)-[#7&H2,$([#7;!H0]-[#6;X4]),$([#7](-[#6X4])-[#6X4])])-[#7&H2,$([#7;!H0]-[#6;X4]),$([#7](-[#6X4])-[#6X4])] <regId=anil_di_alk_F(14)>
2. c:1(:c(:c(:c(:c(:c:1-[#1])-[#1])-[#7](-[#1])-[#1])-[#1])-[#1])-[#6]=[#7]-[#7]-[#1] <regId=hzone_anil(14)>
3. c1(nn(c([c;!H0,$(c-[#6;!H0])]1)-[#8]-[#1])-c:2:c(:c(:c(:c(:c:2-[#1])-[#1])-[#1])-[#1])-[#1])-[#6;X4] <regId=het_5_pyrazole_OH(14)>
4. c:2(:c:1-[#16]-c:3:c(-[#7;!H0,$([#7]-[CH3]),$([#7]-[#6;!H0;!H1]-[#6;!H0])](-c:1:c(:c(:c:2-[#1])-[#1])-[#1])):[c;!H0,$(c~[#7](-[#1])-[#6;X4]),$(c~[#6]:[#6])](:[c;!H0,$(c~[#6]:[#6])]:[c;!H0,$(c-[#7](-[#1])-[#1]),$(c-[#8]-[#6;X4])]:c:3-[#1]))-[#1] <regId=het_thio_666_A(13)>
5. [#6]-2-[#6]-c:1:c(:c:c:c:c:1)-[#6](-c:3:c:c:c:c:c-2:3)=[#6]-[#6] <regId=styrene_A(13)>
6. [#16]-1-[#6](=[#7]-[#6]:[#6])-[#7;!H0,$([#7]-[#6](-[#1])(-[#1])-[#6](-[#1])(-[#1])-[#8]),$([#7]-[#6]:[#6])]-[#6](=[#8])-[#6]-1=[#6](-[#1])-[$([#6]:[#6]:[#6]-[#17]),$([#6]:[!#6&!#1])] <regId=ene_rhod_C(13)>
7. [#7](-[#1])(-[#1])-[#6]-1=[#6](-[#6]#[#7])-[#6](-[#1])(-[#6]:[#6])-[#6](=[#6](-[#6]=[#6])-[#8]-1)-[#6](-[#1])-[#1] <regId=dhp_amino_CN_A(13)>
8. [#8]=[#16](=[#8])-[#6](-[#6]#[#7])=[#7]-[#7]-[#1] <regId=cyano_imine_C(12)>
9. c:1:c:c:c:c:c:1-[#7](-[#1])-[#6](=[#16])-[#7](-[#1])-[#6](-[#1])(-[#1])-[#6](-[#1])(-[#1])-[#6](-[#1])(-[#1])-[#7](-[#6](-[#1])-[#1])-c:2:c:c:c:c:c:2 <regId=thio_urea_A(12)>
10. c:1:c(:c:c:c:c:1)-[#7](-[#1])-c:2:c(:c(:c(:s:2)-[$([#6]=[#8]),$([#6]#[#7]),$([#6](-[#8]-[#1])=[#6])])-[#7])-[$([#6]#[#7]),$([#6](:[#7]):[#7])] <regId=thiophene_amino_B(12)>
11. [#6;X4]-1-[#6](=[#8])-[#7]-[#7]-[#6]-1=[#8] <regId=keto_keto_beta_B(12)>
12. c:1:c-3:c(:c:c:c:1)-[#6]:2:[#7]:[!#1]:[#6]:[#6]:[#6]:2-[#6]-3=[#8] <regId=keto_phenone_A(11)>
13. [#6]-1(-[#6](=[#6](-[#6]#[#7])-[#6](~[#8])~[#7]~[#6]-1~[#8])-[#6](-[#1])-[#1])=[#6](-[#1])-[#6]:[#6] <regId=cyano_pyridone_C(11)>

14. [#6]-1(=[#6](-!@[#6]=[#7])-[#16]-[#6](-[#7]-1)=[#8])-[$([F,Cl,Br,I]),$([#7+](:[#6]):[#6])] <regId=thiaz_ene_C(11)>
15. c:1:2:c(:c(:c(:c(:c:1-[#1])-[#1])-[#1])-[#1]):[!#6&!#1]:[#6;!H0,$([#6]-[OH]),$([#6]-[#6;H2,H3])](:[#6]:2-[#6](-[#1])=[#7]-[#7](-[#1])-[$([#6]:1:[#7]:[#6]:[#6](-[#1]):[#16]:1),$([#6]:[#6](-[#1]):[#6]-[#1]),$([#6]:[#7]:[#6]:[#7]:[#6]:[#7]),$([#6]:[#7]:[#7]:[#7]:[#7])]) <regId=hzone_thiophene_A(11)>
16. [!#1]:[!#1]-[#6;!H0,$([#6]-[#6]#[#7])]=[#6]-1-[#6]=,:[#6]-[#6](=[$([#8]),$([#7;!R])])-[#6]=,:[#6]-1 <regId=ene_quin_methide(10)>
17. c:1:c:c-2:c(:c:c:1)-[#6]-[#6](-c:3:c(-[#16]-2):c(:c(-[#1]):[c;!H0,$(c-[#8]),$(c-[#16;X2]),$(c-[#6;X4]),$(c-[#7;H2,H3,$([#7!H0]-[#6;X4]),$([#7](-[#6;X4])-[#6;X4])])](:c:3-[#1]))-[#1])-[#7;H2,H3,$([#7;!H0]-[#6;X4]),$([#7](-[#6;X4])-[#6;X4])] <regId=het_thio_676_A(10)>
18. [#6]-1(=[#8])-[#6](=[#6](-[#1])-[$([#6]:1:[#6]:[#6]:[#6]:[#6]:[#6]:1),$([#6]:1:[#6]:[#6]:[#6]:[!#6&!#1]:1)])-[#7]=[#6](-[!#1]:[!#1]:[!#1])-[$([#16]),$([#7]-[!#1]:[!#1])]-1 <regId=ene_five_het_G(10)>
19. [#7+](:[!#1]:[!#1]:[!#1])-[!#1]=[#8] <regId=acyl_het_A(9)>
20. [#6;X4]-[#7](-[#6;X4])-c:1:c(:c(:c(:c(:c:1-[#1])-[#1])-[#6]2=,:[#7][#6]:[#6]:[!#1]2)-[#1])-[#1] <regId=anil_di_alk_G(9)>
21. [#7;!H0,$([#7]-[#6;X4])]-1-[#6]=,:[#6](-[#6](=[#8])-[#6]:[#6]:[#6])-[#6](-[#6])-[#6](=[#6]-1-[#6](-[#1])(-[#1])-[#1])-[$([#6]=[#8]),$([#6]#[#7])] <regId=dhp_keto_A(9)>
22. c:1:c:c:c:c:c:1-[#7](-[#1])-[#6](=[#16])-[#7](-[#1])-[#6](-[#1])(-[#1])-[#6](-[#1])(-[#1])-[#7](-[#6](-[#1])-[#1])-c:2:c:c:c:c:c:2 <regId=thio_urea_B(9)>
23. c:1:3:c(:c(:c(:c(:c:1-[#1])-[#1])-[#7](-[#1])-[#6](-[#1])(-[#1])-c:2:c:c:c:c:c:2)-[#1]):n:c(-[#1]):n:3-[#6] <regId=anil_alk_bim(9)>
24. c:1:c:c-2:c(:c:c:1)-[#7]=[#6]-[#6]-2=[#7;!R] <regId=imine_imine_A(9)>
25. c:1(:c:c:c:c:c:1)-[#7](-[#1])-[#6](=[#16])-[#7]-[#7](-[#1])-[#6](=[#8])-[#6]-,:2:[!#1]:[!#6&!#1]:[#6]:[#6]-,:2 <regId=thio_urea_C(9)>
26. [#7;!R]=[#6]-2-[#6](=[#8])-c:1:c:c:c:c:c:1-[#16]-2 <regId=imine_one_fives_B(9)>
27. [$([#7](-[#1])-[#1]),$([#8]-[#1])]-[#6]-2=[#6](-[#6]#[#7])-[#6](-[#1])(-[#6]:[#6])-c:1:c(:n(-[#6]):n:c:1)-[#8]-2 <regId=dhp_amino_CN_B(9)>
28. [#7](-[#1])(-[#1])-c:1:c(:c(:c(:n:c:1-[#1])-[#8]-c:2:c:c:c:c:c:2)-[#1])-[#1] <regId=anil_OC_no_alk_A(8)>
29. [#6](=[#8])-[#6]-1=[#6]-[#7]-c:2:c(-[#16]-1):c:c:c:c:2 <regId=het_thio_66_one(8)>
30. c:1:c:c-2:c(:c:c:1)-[#6](-c:3:c(-[$([#16;X2]),$([#6;X4])]-2):c:c:[c;!H0,$(c-[#17]),$(c-[#6;X4])](:c:3))=[#6]-[#6] <regId=styrene_B(8)>
31. [#6](-[#1])(-[#1])-[#16;X2]-c:1:n:c(:c(:n:1-!@[#6](-[#1])-[#1])-c:2:c:c:c:c:c:2)-[#1] <regId=het_thio_5_A(8)>
32. [#6](-[#1])(-[#1])-[#7](-[#6](-[#1])-[#1])-[#6]-2=[#6](-[#1])-c:1:c(:c:c:c:c:1)-[#16;X2]-c:3:c-2:c:c:c:c:3 <regId=anil_di_alk_ene_A(8)>
33. [#16]-1-[#6](=!@[#7;!H0,$([#7]-[#7](-[#1])-[#6]:[#6])])-[#7;!H0,$([#7]-[#6]:[#7]:[#6]:[#6]:[#16])]-[#6](=[#8])-[#6]-1=[#6](-[#1])-[#6]:[#6]-[$([#17]),$([#8]-[#6]-[#1])] <regId=ene_rhod_D(8)>
34. [#16]-1-[#6](=[#8])-[#7]-[#6](=[#16])-[#6]-1=[#6](-[#1])-[#6]:[#6] <regId=ene_rhod_E(8)>
35. c:1:c(:c:c:c:c:1)-[#6](-[#1])(-[#1])-[#7](-[#1])-c:2:c(:c(:c(:c(:c:2-[#1])-[#1])-[#8]-[#1])-[#1])-[#1] <regId=anil_OH_alk_A(8)>
36. n1(-[#6;X4])c(c(-[#1])c(c1-[#6]:[#6])-[#1])-[#6](-[#1])-[#1] <regId=pyrrole_C(8)>
37. c:1(:c:c:c:c:c:1)-[#7](-[#1])-[#6](=[#16])-[#7]-[#7](-[#1])-c:2:c:c:c:c:c:2 <regId=thio_urea_D(8)>
38. [#7](-c:1:c:c:c:c:c:1)-c2[n+]c(cs2)-c:3:c:c:c:c:c:3 <regId=thiaz_ene_D(8)>
39. n:1:c:c:c(:c:1-[#6](-[#1])-[#1])-[#6](-[#1])=[#6]-2-[#6](=[#8])-[#7]-[#6](=[!#6&!#1])-[#7]-2 <regId=ene_rhod_F(8)>
40. [#6]-,:1(=,:[#6](-[#6](-[#1])(-[#6])-[#6])-,:[#16]-,:[#6](-,:[#7;!H0,$([#7]-[#6;!H0;!H1])]-,:1)=[#8])-[#16]-[#6;R] <regId=thiaz_ene_E(8)>
41. [!#1]:,-1:[!#1]-,:2:[!#1](:[!#1]:[!#1]:[!#1]:,-1)-,:[#7](-[#1])-,:[#7](-,:[#6]-,:2=[#8])-[#6] <regId=het_65_B(7)>
42. c:1:c:c-2:c(:c:c:1)-[#6](=[#6](-[#6]-2=[#8])-[#6])-[#8]-[#1] <regId=keto_keto_beta_C(7)>
43. c:2:c:c:1:n:n:c(:n:c:1:c:c:2)-[#6](-[#1])(-[#1])-[#6]=[#8] <regId=het_66_A(7)>
44. c:1:c:c:c:c:c:1-[#7](-[#1])-[#6](=[#16])-[#7](-[#1])-[#6](-[#1])(-[#1])-c:2:n:c:c:c:c:2 <regId=thio_urea_E(7)>
45. [#6](-[#1])-[#6](-[#1])(-[#1])-c:1:c(:c(:c(:s:1)-[#7](-[#1])-[#6](=[#8])-[#6]-[#6]-[#6]=[#8])-[$([#6](=[#8])-[#8]),$([#6]#[#7])])-[#6](-[#1])-[#1] <regId=thiophene_amino_C(7)>
46. [#6](-c:1:c(:c(:[c;!H0,$(c-[#6;X4])]:c:c:1-[#1])-[#1])-[#1])(-c:2:c(:c(:[c;!H0,$(c-[#17])](:c(:c:2-[#1])-[#1]))-[#1])-[#1])=[$([#7]-[#8]-[#6](-[#1])(-[#1])-[#6](-[#1])(-[#1])-[#6](-[#1])(-[#1])-[#7](-[#6](-[#1])-[#1])-[#6](-[#1])-[#1]),$([#7]-[#8]-[#6](-[#1])(-[#1])-[#6](-[#1])(-[#1])-[#7](-[#6](-[#1])-[#1])-[#6](-[#1])-[#1]),$([#7]-[#7](-[#1])-[#6](=[#7]-[#1])-[#7](-[#1])-[#1]),$([#6](-[#1])-[#7])] <regId=hzone_phenone(7)>
47. [#8](-[#1])-[#6](=[#8])-c:1:c:c(:c:c:c:1)-[#6]:[!#1]:[#6]-[#6](-[#1])=[#6]-2-[#6](=[!#6&!#1])-[#7]-[#6](=[!#6&!#1])-[!#6&!#1]-2 <regId=ene_rhod_G(7)>
48. [#6]-1(=[#6]-[#6](-c:2:c:c(:c(:n:c-1:2)-[#7](-[#1])-[#1])-[#6]#[#7])=[#6])-[#6]#[#7] <regId=ene_cyano_B(7)>
49. [#7](-[#1])(-[#1])-[#6]-1=[#6](-[#6]#[#7])-[#6](-[#1])(-[#6]:[#6])-[#6](=[#6](-[#6]:[#6])-[#8]-1)-[#6]#[#7] <regId=dhp_amino_CN_C(7)>
50. [#7]-2(-c:1:c:c:c:c:c:1)-[#7]=[#6](-[#6]=[#8])-[#6;X4]-[#6]-2=[#8] <regId=het_5_A(7)>
51. [#7]-1=[#6]-[#6](-[#6](-[#7]-1)=[#16])=[#6] <regId=ene_five_het_H(6)>
52. c1(coc(c1-[#1])-[#6](=[#16])-[#7]-2-[#6](-[#1])(-[#1])-[#6](-[#1])(-[#1])-[!#1]-[#6](-[#1])(-[#1])-[#6]-2(-[#1])-[#1])-[#1] <regId=thio_amide_A(6)>
53. [#6]=[#6](-[#6]#[#7])-[#6](=[#7]-[#1])-[#7]-[#7] <regId=ene_cyano_C(6)>

54. c:1(:c(:c(:[c;!H0,$(c-[#6;!H0;!H1])](:o:1))-[#1])-[#1])-[#6;!H0,$([#6]-[#6;!H0;!H1])]=[#7]-[#7](-[#1])-c:2:n:c:c:s:2 <regId=hzone_furan_A(6)>
55. c:1(:c(:c(:c(:c(:c:1-[#7](-[#1])-[#16](=[#8])(=[#8])-[#6]:2:[#6]:[!#1]:[#6]:[#6]:[#6]:2)-[#1])-[#7](-[#6](-[#1])-[#1])-[#6](-[#1])-[#1])-[#1])-[#1])-[#1] <regId=anil_di_alk_H(6)>
56. n2c1ccccn1c(c2-[$([#6](-[!#1])=[#6](-[#1])-[#6]:[#6]),$([#6]:[#8]:[#6])])-[#7]-[#6]:[#6] <regId=het_65_C(6)>
57. [#6]-1-[#7](-[#1])-[#7](-[#1])-[#6](=[#16])-[#7]-[#7]-1-[#1] <regId=thio_urea_F(6)>
58. c:1(:c:c:c:o:1)-[#6](-[#1])=!@[#6]-3-[#6](=[#8])-c:2:c:c:c:c:c:2-[!#6&!#1]-3 <regId=ene_five_het_I(6)>
59. [#8]=[#6]-1-[#6;X4]-[#6]-[#6](=[#8])-c:2:c:c:c:c:c-1:2 <regId=keto_keto_gamma(5)>
60. c:1:c:c-2:c(:c:c:1)-[#6](-c3cccc4noc-2c34)=[#8] <regId=quinone_B(5)>
61. [#8](-[#1])-c:1:n:c(:c:c:c:1)-[#8]-[#1] <regId=het_6_pyridone_OH(5)>
62. c:1:2:c(:c(:c(:c(:c:1:c(:c(:c(:c:2-[#1])-[#1])-[#6]=[#7]-[#7](-[#1])-[$([#6]:[#6]),$([#6]=[#16])])-[#1])-[#1])-[#1])-[#1])-[#1] <regId=hzone_naphth_A(5)>
63. [#6]-,:1=,:[#6](-,:[#16]-,:[#6](-,:[#6]=,:[#6]-,:1)=[#16])-,:[#7] <regId=thio_ester_A(5)>
64. [#6]-1=[#6]-[#6](-[#8]-[#6]-1-[#8])(-[#8])-[#6] <regId=ene_misc_A(5)>
65. [#8]=[#6]-,:1-,:[#6](=,:[#6]-,:[#6](=,:[#7]-,:[#7]-,:1)-,:[#6]=[#8])-[#6]#[#7] <regId=cyano_pyridone_D(5)>
66. c3cn1c(nc(c1-[#7]-[#6])-c:2:c:c:c:c:n:2)cc3 <regId=het_65_Db(5)>
67. [#7]-2-c:1:c:c:c:c:c:1-[#6](=[#7])-c:3:c-2:c:c:c:c:3 <regId=het_666_A(5)>
68. c:1:c(:c:c:c:c:1)-[#7]-2-[#6](-[#1])-[#6](-[#1])-[#7](-[#6](-[#1])-[#6]-2-[#1])-[#16](=[#8])(=[#8])-c:3:c:c:c:c:4:n:s:n:c:3:4 <regId=diazox_sulfon_B(5)>
69. c:1(:c(:c-,:2:c(:c(:c:1-[#1])-[#1])-,:[#7](-,:[#6](-,:[#7]-,:2-[#1])=[#8])-[#1])-[#1])-[#7](-[#1])-[#6](-[#1])-[#1] <regId=anil_NH_alk_A(5)>
70. c:1(:c(:c-3:c(:c(:c:1-[#7](-[#1])-[#16](=[#8])(=[#8])-c:2:c:c:c(:c:c:2)-[!#6&!#1])-[#1])-[#8]-[#6](-[#8]-3)(-[#1])-[#1])-[#1])-[#1] <regId=sulfonamide_C(5)>
71. [#6](-[#1])-[#6]:2:[#7]:[#7](-c:1:c:c:c:c:c:1):[#16]:3:[!#6&!#1]:[!#1]:[#6]:[#6]:2:3 <regId=het_thio_N_55(5)>
72. [#8]=[#6]-[#6]=[#6](-[#1])-[#8]-[#1] <regId=keto_keto_beta_D(5)>
73. [#7]-,:1-,:2-,:[#6](=,:[#7]-,:[#6](=[#8])-,:[#6](=,:[#7]-,:1)-[#6](-[#1])-[#1])-,:[#16]-,:[#6](=[#6](-[#1])-[#6]:[#6])-,:[#6]-,:2=[#8] <regId=ene_rhod_H(5)>
74. [#6]:[#6]-[#6](-[#1])=[#6](-[#1])-[#6](-[#1])=[#7]-[#7](-[#6;X4])-[#6;X4] <regId=imine_ene_A(5)>
75. c:1:3:c(:c:c:c:c:1):c:2:n:n:c(-[#16]-[#6](-[#1])(-[#1])-[#6]=[#8]):n:c:2:n:3-[#6](-[#1])(-[#1])-[#6](-[#1])=[#6](-[#1])-[#1] <regId=het_thio_656a(5)>
76. n1(-[#6])c(c(-[#1])c(c1-[#6](-[#1])(-[#1])-[#7](-[#1])-[#6](=[#16])-[#7]-[#1])-[#1])-[#1]<regId=pyrrole_D(5)>
77. n2(-[#6]:1:[!#1]:[!#6&!#1]:[!#1]:[#6]:1-[#1])c(c(-[#1])c(c2-[#6;X4])-[#1])-[#6;X4] <regId=pyrrole_E(5)>
78. c:1(:c:c:c:c:c:1)-[#7](-[#1])-[#6](=[#16])-[#7]-[#7](-[#1])-[#6]([#7;R])[#7;R] <regId=thio_urea_G(5)>
79. c:1(:c(:c(:c(:c(:[c;!H0,$(c-[#6](-[#1])-[#1])]:1)-[#1])-[#8]-[#6](-[#1])-[#1])-[#6](-[#1])(-[#1])-[$([#7](-[#1])-[#6](=[#8])-[#6](-[#1])(-[#1])-[#6](-[#1])(-[#1])-[#6](-[#1])-[#1]),$([#6](-[#1])(-[#6](-[#1])-[#1])-[#7](-[#1])-[#6](=[#16])-[#7]-[#1])])-[#1])-[#8]-[#6](-[#1])-[#1] <regId=anisol_A(5)>
80. n2(-[#6]:1:[#6](-[#6]#[#7]):[#6]:[#6]:[!#6&!#1]:1)c(c(-[#1])c(c2)-[#1])-[#1] <regId=pyrrole_F(5)>
81. [#7](-[#1])(-[#1])-[#6]-2=[#6](-[#6]#[#7])-[#6](-[#1])(-[#6]:[#6])-c:1:c(:c:c:s:1)-[#8]-2 <regId=dhp_amino_CN_D(5)>
82. [#7](-[#1])-c:1:n:c(:c:s:1)-c:2:c:n:c(-[#7](-[#1])-[#1]):s:2 <regId=thiazole_amine_A(4)>
83. [#7]=[#6]-1-[#7](-[#1])-[#6](=[#6](-[#7]-[#1])-[#7]=[#7]-1)-[#7]-[#1] <regId=het_6_imidate_A(4)>
84. c:1:c(:c:2:c(:c:c:1):c:c:c:c:2)-[#8]-c:3:c(:c(:c(:c(:c:3-[#1])-[#1])-[#7]-[#1])-[#1])-[#1] <regId=anil_OC_no_alk_B(4)>
85. c:1:c:c-2:c(:c:c:1)-[#6]-[#16]-c3c(-[#6]-2=[#6])ccs3 <regId=styrene_C(4)>
86. c:2:c:c:c:1:c(:c:c:c:1):c:c:2 <regId=azulene(4)>
87. c:1(:c(:c(:c(:o:1)-[#6](-[#1])-[#1])-[#6](-[#1])(-[#1])-[#8]-[#6]:[#6])-[#1])-[#6](=[#8])-[#8]-[#1] <regId=furan_acid_A(4)>
88. [!#1]:[#6]-[#6]-,:1=,:[#6](-[#1])-,:[#6](=,:[#6](-[#6]#[#7])-,:[#6](=[#8])-,:[#7]-,:1-[#1])-[#6]:[#8] <regId=cyano_pyridone_E(4)>
89. [#6]-1-3=[#6](-[#6](-[#7]-c:2:c:c:c:c:c-1:2)(-[#6])-[#6])-[#16]-[#16]-[#6]-3=[!#1] <regId=anil_alk_thio(4)>
90. c:1(:c(:c(:c(:c(:c:1-[#7](-[#1])-[#6](=[#8])-c:2:c:c:c:c:c:2)-[#1])-[#7](-[#6](-[#1])-[#1])-[#6](-[#1])-[#1])-[#1])-[#1])-[#1] <regId=anil_di_alk_I(4)>
91. [#6](-[#1])(-[#1])-[#16;X2]-c:1:n:n:c(:c(:n:1)-c:2:c(:c(:c(:o:2)-[#1])-[#1])-[#1])-c:3:c(:c(:c(:o:3)-[#1])-[#1])-[#1] <regId=het_thio_6_furan(4)>
92. [#6](-[#1])(-[#1])-[#7](-[#6](-[#1])-[#1])-[#6]-2=[#6]-c:1:c(:c:c:c:c:1)-[#6]-2(-[#1])-[#1] <regId=anil_di_alk_ene_B(4)>
93. [#7](-[#1])(-c:1:c:c:c:c:c:1)-[#7]=[#6](-[#6](=[#8])-[#6](-[#1])-[#1])-[#7](-[#1])-[$([#7]-[#1]),$([#6]:[#6])] <regId=imine_one_B(4)>
94. c:1:2:c(:c(:c(:c(:c:1-[#1])-[#1])-[#1])-[#1]):o:c:3:c(-[#1]):c(:c(-[#8]-[#6](-[#1])-[#1]):c(:c:2:3)-[#1])-[#7](-[#1])-[#6](-[#1])-[#1] <regId=anil_OC_alk_A(4)>
95. [#16]=[#6]-,:1-,:[#7](-[#1])-,:[#6]=,:[#6]-,:[#6]-2=,:[#6]-,:1-[#6](=[#8])-[#8]-[#6]-2=[#6]-[#1] <regId=ene_five_het_J(4)>
96. n2(-c:1:c(:c:c(:c(:c:1)-[#1])-[$([#7](-[#1])-[#1]),$([#6]:[#7])])-[#1])c(c(-[#1])c(c2-[#1])-[#1])-[#1] <regId=pyrrole_G(4)>
97. n1(-[#6])c(c(-[#1])c(c1-[#6](-[#1])=[#6]-2-[#6](=[#8])-[!#6&!#1]-[#6]=,:[!#1]-2)-[#1])-[#1] <regId=ene_five_het_K(4)>

98. [#6]=[#6]-[#6](-[#6]#[#7])(-[#6]#[#7])-[#6](-[#6]#[#7])=[#6]-[#7](-[#1])-[#1]<regId=cyano_ene_amine_B(4)>
99. [#6]:[#6]-[#6](=[#16;X1])-[#16;X2]-[#6](-[#1])-[$([#6](-[#1])-[#1]),$([#6]:[#6])] <regId=thio_ester_B(4)>
100. [#8]=[#6]-3-[#6](=!@[#6](-[#1])-c:1:c:n:c:c:1)-c:2:c:c:c:c:c:2-[#7]-3 <regId=ene_five_het_L(4)>
101. c:1(:[c;!H0,$(c-[#6;!H0;!H1])](:c(:c(:s:1)-[#1])-[#1]))-[#6](-[#1])=[#7]-[#7](-[#1])-c:2:c:c:c:c:c:2 <regId=hzone_thiophene_B(4)>
102. [#6](-[#1])(-[#1])-[#16;X2]-[#6]-1=[#6](-[#6]#[#7])-[#6](-[#1])(-[#6]:[#6])-[#6](-[#6]#[#7])-[#6](=[#8])-[#7]-1 <regId=dhp_amino_CN_E(4)>
103. [#7]-2(-c:1:c:c:c:c:c:1)-[#7]=[#6](-[#7](-[#1])-[#6]=[#8])-[#6](-[#1])(-[#1])-[#6]-2=[#8]<regId=het_5_B(4)>
104. [#6]:[#6]-[#6](-[#1])=[#6](-[#1])-[#6](-[#1])=[#7]-[#7]=[#6] <regId=imine_imine_B(3)>
105. c:1(:c:c:c(:c:c:1)-[#6](-[#1])-[#1])-c:2:c(:s:c(:n:2)-[#7](-[#1])-[#1])-[#6](-[#1])(-[#1])-[#1] <regId=thiazole_amine_B(3)>
106. [#6]-2(-[#6]=[#7]-c:1:c:c:c:c:c:1-[#7]-2)=[#6](-[#1])-[#6]=[#8] <regId=imine_ene_one_A(3)>
107. [#8](-c:1:c:c:c:c:c:1)-c:3:c:c:2:n:o:n:c:2:c:c:3 <regId=diazox_A(3)>
108. [!#1]:1:[!#1]:[!#1]:[!#1](:[!#1]:[!#1]:1)-[#6](-[#1])=[#6](-[#1])-[#6](-[#7]-c:2:c:c:c:3:c(:c:2):c:c:c(:n:3)-[#7](-[#6])-[#6])=[#8] <regId=ene_one_A(3)>
109. [#7](-[#1])(-[#1])-c:1:c(:c:c:c:n:1)-[#8]-[#6](-[#1])(-[#1])-[#6]:[#6] <regId=anil_OC_no_alk_C(3)>
110. [#6]-[#16;X2]-c:1:n:c(:c:s:1)-[#1] <regId=thiazol_SC_A(3)>
111. c:1:c-3:c(:c:c:c:1)-[#7](-c:2:c:c:c:c:c:2-[#8]-3)-[#6](-[#1])(-[#1])-[#6](-[#1])-[#1] <regId=het_666_B(3)>
112. c:1(:c(:c(:c(:o:1)-[#6](-[#1])-[#1])-[#1])-[#1])-[#6](-[#1])(-[#8]-[#1])-[#6]#[#6]-[#6;X4]<regId=furan_A(3)>
113. [#6]-1(-[#6](=[#6]-[#6]=[#6]-[#6]=[#6]-1)-[#7]-[#1])=[#7]-[#6] <regId=colchicine_A(3)>
114. [#6](-[#1])(-[#1])-[#7](-[#6](-[#1])-[#1])-[#6](-[#1])=[#6]-[#6](=[#8])-c:1:c(-[#16;X2]):s:c(:c:1)-[$([#6]#[#7]),$([#6]=[#8])] <regId=thiophene_C(3)>
115. c:1:3:c(:c:c:c:c:1)-[#7]-2-[#6](=[#8])-[#6](=[#6](-[F,Cl,Br,I])-[#6]-2=[#8])-[#7](-[#1])-[#6]:[#6]:[#6]:[#6](-[#8]-[#6](-[#1])-[#1]):[#6]:[#6]:3 <regId=anil_OC_alk_B(3)>
116. c:1-2:c(:c:c:c:c:1)-[#6](-[#1])(-[#1])-[#6](-[#1])(-[#1])-[#7]=[#6]-2-[#16;X2]-[#6](-[#1])(-[#1])-[#6](=[#8])-c:3:c:c:c:c:c:3 <regId=het_thio_66_A(3)>
117. [#7]-2(-c:1:c:c:c:c:c:1-[#6](-[#1])-[#1])-[#6](=[#16])-[#7](-[#6](-[#1])(-[#1])-[!#1]:[!#1]:[!#1]:[!#1]:[!#1])-[#6](-[#1])(-[#1])-[#6]-2=[#8] <regId=rhod_sat_B(3)>
118. [#7]-2(-[#6](-[#1])-[#1])-[#6](=[#16])-[#7](-[#1])-[#6](=[#6](-[#1])-c:1:c:c:c:c(:c:1)-[Br])-[#6]-2=[#8] <regId=ene_rhod_I(3)>
119. c:1(:c(:c:2:c(:s:1):c:c:c:c:2)-[#6](-[#1])-[#1])-[#6](=[#8])-[#6](-[#1])(-[#1])-[#6](-[#1])-[#1] <regId=keto_thiophene(3)>
120. [#7](-[#6](-[#1])-[#1])(-[#6](-[#1])-[#1])-[#6](-[#1])=[#7]-[#6](-[#6](-[#1])-[#1])=[#7]-[#7](-[#6](-[#1])-[#1])-[#6]:[#6] <regId=imine_imine_C(3)>
121. [#6]:2(:[#6](-[#6](-[#1])-[#1]):[#6]-,:1:[#6](-,:[#7]=,:[#6;!H0,$([#6]-[#16]-[#6](-[#1])-[#1])](-,:[#7](-,:[#6]-,:1=[!#6&!#1;X1])-[#6](-[#1])-[$([#6](=[#8])-[#8]),$([#6]:[#6])])):[!#6&!#1;X2]:2)-[#6](-[#1])(-[#1])-[#6](-[#1])-[#1] <regId=het_65_pyridone_A(3)>
122. c:1(:n:c(:c(-[#1]):s:1)-[!#1]:[!#1]:[!#1](-[$([#8]-[#6](-[#1])-[#1]),$([#6](-[#1])-[#1])]):[!#1]:[!#1])-[#7](-[#1])-[#6](-[#1])(-[#1])-c:2:c(-[#1]):c(:c(-[#1]):o:2)-[#1] <regId=thiazole_amine_C(3)>
123. n:1:c(:c(:c(:c:1-[#16]-[#6]-[#1])-[#6]#[#7])-c:2:c:c:c(:c:c:2)-[#8]-[#6](-[#1])-[#1])-[#1])-[#6]:[#6] <regId=het_thio_pyr_A(3)>
124. c:1:4:c(:n:c(:n:c:1-[#7](-[#1])-[#6](-[#1])(-[#1])-c:2:c(:c(:c(:o:2)-[#1])-[#1])-[#1])-[#7](-[#1])-c:3:c:[c;!H0,$(c-[#6](-[#1])-[#1]),$(c-[#16;X2]),$(c-[#8]-[#6]-[#1]),$(c-[#7;X3])](:[c;!H0,$(c-[#6](-[#1])-[#1]),$(c-[#16;X2]),$(c-[#8]-[#6]-[#1]),$(c-[#7;X3])](:c:[c;!H0,$(c-[#6](-[#1])-[#1]),$(c-[#16;X2]),$(c-[#8]-[#6]-[#1]),$(c-[#7;X3])]:3))):c:c:c:c:4 <regId=melamine_A(3)>
125. [#7](-[#1])(-[#6]:1:[#6]:[#6]:[!#1]:[#6]:[#6]:1)-c:2:c:c:c(:c:c:2)-[#7](-[#1])-[#6]-[#1] <regId=anil_NH_alk_B(3)>
126. [#7]-2(-c:1:c:c:c:c:c:1)-[#6](=[#7]-[#6]=[#8])-[#16]-[#6](-[#1])(-[#1])-[#6]-2=[#8] <regId=rhod_sat_C(3)>
127. [#6]=[#6]-[#6](=[#8])-[#7]-c:1:c(:c(:c(:s:1)-[#6](=[#8])-[#8])-[#6]-[#1])-[#6]#[#7] <regId=thiophene_amino_D(3)>
128. [#8;!H0,$([#8]-[#6](-[#1])-[#1])]-c:1:c(:c(:c(:c(:c:1-[#1])-[#1])-[#1])-[#1])-[#7](-[#1])-[#6](-[#1])(-[#1])-c:2:n:c:c:n:2 <regId=anil_OC_alk_C(3)>
129. [#6](-[#1])(-[#1])-[#16;X2]-c3nc1c(n(nc1-[#6](-[#1])-[#1])-c:2:c:c:c:c:c:2)nn3 <regId=het_thio_65_A(3)>
130. [#6]-[#6](=[#8])-[#6](-[#1])(-[#1])-[#16;X2]-c:3:n:n:c:2:c:1:c(:c(:c(:c(:c:1:n(:c:2:n:3)-[#1])-[#1])-[#1])-[#1])-[#1] <regId=het_thio_656b(3)>
131. s:1:c(:[n+](-[#6](-[#1])-[#1]):c(:c:1-[#1])-[#6])-[#7](-[#1])-c:2:c:c:c:c:c:2[$([#6](-[#1])-[#1]),$([#6]:[#6])] <regId=thiazole_amine_D(3)>
132. [#6]-,:2(=[#16])-,:[#7](-[#6](-[#1])(-[#1])-c:1:c:c:c:o:1)-,:[#6](=,:[#7]-,:[#7]-,:2-[#1])-[#6]:[#6] <regId=thio_urea_H(3)>
133. [#7]-,:2(-c:1:c:c:c:c:c:1)-,:[#6](=[#8])-,:[#6](=,:[#6]-,:[#6](=,:[#7]-,:2)-[#6]#[#7])-[#6]#[#7] <regId=cyano_pyridone_F(3)>
134. [#7]-2(-c:1:c:c:c:c:c:1)-[#6](=[#8])-[#16]-[#6](-[#1])(-[#6](-[#1])(-[#1])-[#6](=[#8])-[#7](-[#1])-[#6]:[#6])-[#6]-2=[#8] <regId=rhod_sat_D(3)>
135. [#6](-[#1])(-[#1])-[#7]-2-[#6](=[$([#16]),$([#7])])-[!#6&!#1]-[#6](=[#6]-1-[#6](=[#6](-[#1])-[#6]:[#6]-[#7]-1-[#6](-[#1])-[#1])-[#1])-[#6]-2=[#8] <regId=ene_rhod_J(3)>
136. [#6]=[#7;!R]-c:1:c:c:c:c:c:1-[#8]-[#1] <regId=imine_phenol_A(3)>
137. [#8]=[#6]-,:2-,:[#16]-,:c:1:c(:c(:c:c:c:1)-[#8]-[#6](-[#1])-[#1])-,:[#8]-,:2 <regId=thio_carbonate_B(3)>

138. [#7]=,:[#6]-,:1-,:[#7]=,:[#6]-,:[#7]-,:[#16]-,:1 <regId=het_thio_N_5A(3)>
139. [#7]-,:2-,:[#16]-,:[#6]-1=,:[#6](-[#6]:[#6]-[#7]-[#6]-1)-,:[#6]-,:2=[#16] <regId=het_thio_N_65A(3)>
140. [#6](-[#1])(-[#1])-[#7](-[#6](-[#1])-[#1])-c:1:c(:c(:c(:c(:c:1-[#1])-[#1])-[#6](-[#1])=[#7]-[#7]=[#6](-[#6])-[#6]:[#6])-[#1])-[#1] <regId=anil_di_alk_J(3)>
141. n1-2cccc1-[#6]=[#7](-[#6])-[#6]-[#6]-2 <regId=pyrrole_H(3)>
142. [#6](-[#6]#[#7])(-[#6]#[#7])=[#6](-[#16])-[#16] <regId=ene_cyano_D(3)>
143. [#6]-1(-[#6]#[#7])(-[#6]#[#7])-[#6](-[#1])(-[#6](=[#8])-[#6])-[#6]-1-[#1] <regId=cyano_cyano_B(3)>
144. [#6]-1=,:[#6]-[#6](-[#6](-[$([#8]),$([#16])]-1)=[#6]-[#6]=[#8])=[#8] <regId=ene_five_het_M(3)>
145. [#6]:[#6]-[#6](=[#8])-[#7](-[#1])-[#6](=[#8])-[#6](-[#6]#[#7])=[#6](-[#1])-[#7](-[#1])-[#6]:[#6] <regId=cyano_ene_amine_C(3)>
146. c:1(:c:c:c:c:c:1)-[#7](-[#1])-[#6](=[#16])-[#7](-[#1])-[#7]=[#6]-c:2:c:n:c:c:2 <regId=thio_urea_I(3)>
147. [#7](-[#1])(-[#1])-[#6]-2=[#6](-[#6]#[#7])-[#6](-[#1])(-c:1:c:c:c:s:1)-[#6](=[#6](-[#6](-[#1])-[#1])-[#8]-2)-[#6](=[#8])-[#8]-[#6] <regId=dhp_amino_CN_F(3)>
148. c:1:c-3:c(:c:c(:c:1)-[#6](=[#8])-[#7](-[#1])-c:2:c(:c:c:c:c:2)-[#6](=[#8])-[#8]-[#1])-[#6](-[#7](-[#6]-3=[#8])-[#6](-[#1])-[#1])=[#8] <regId=anthranil_acid_B(3)>
149. [Cl]-c:2:c:c:1:n:o:n:c:1:c:c:2 <regId=diazox_B(3)>
150. [#6]-[#6](=[#16])-[#1] <regId=thio_aldehyd_A(3)>
151. [#6;X4]-[#7](-[#1])-[#6](-[#6]:[#6])=[#6](-[#1])-[#6](=[#16])-[#7](-[#1])-c:1:c:c:c:c:c:1<regId=thio_amide_B(2)>
152. [#6](-[#1])(-[#1])-[#6](-[#1])(-[#1])-[#16]-[#6](-[#1])(-[#1])-c1cn(cn1)-[#1] <regId=imidazole_B(2)>
153. [#8]=[#6]-[#7](-[#1])-c:1:c(-[#6]:[#6]):n:c(-[#6](-[#1])(-[#1])-[#6]#[#7]):s:1 <regId=thiazole_amine_E(2)>
154. [#6](-[#1])-[#7](-[#1])-c:1:n:c(:c:s:1)-c2cnc3n2ccs3 <regId=thiazole_amine_F(2)>
155. [#7]-,:1-,:[#6](=[#8])-,:[#6](=,:[#6](-[#6])-,:[#16]-,:[#6]-,:1=[#16])-[#1] <regId=thio_ester_C(2)>
156. [#6](-[#16])(-[#7])=[#6](-[#1])-[#6]=[#6](-[#1])-[#6]=[#8]<regId=ene_one_B(2)>
157. [#8]=[#6]-3-c:1:c(:c:c:c:c:1)-[#6]-2=[#6](-[#8]-[#1])-[#6](=[#8])-[#7]-c:4:c-2:c-3:c:c:c:4 <regId=quinone_C(2)>
158. c:1:2:c:c:c:c(:c:1:c(:c:c:c:2)-[$([#8]-[#1]),$([#7](-[#1])-[#1])])-[#6](-[#6])=[#8] <regId=keto_naphthol_A(2)>
159. [#6](-[#1])(-c:1:c:c:c:c:c:1)(-c:2:c:c:c:c:c:2)-[#6](=[#16])-[#7]-[#1] <regId=thio_amide_C(2)>
160. [#7]-2(-[#6](=[#8])-c:1:c(:c(:c(:c(:c:1-[#1])-[#6](=[#8])-[#8]-[#1])-[#1])-[#1])-[#6]-2=[#8])-c:3:c(:c:c(:c(:c:3)-[#1])-[#8])-[#1] <regId=phthalimide_misc(2)>
161. c:1:c:c(:c:c:c:1-[#7](-[#1])-[#16](=[#8])=[#8])-[#7](-[#1])-[#16](=[#8])=[#8] <regId=sulfonamide_D(2)>
162. [#6](-[#1])-[#7](-[#1])-c:1:c(:c(:c(:c(:c:1-[#1])-[#1])-[#1])-[#1])-[#7](-[#1])-[#6]-[#1] <regId=anil_NH_alk_C(2)>
163. s1c(c(c-,:2c1-,:[#7](-[#1])-,:[#6](-,:[#6](=,:[#6]-,:2-[#1])-[#6](=[#8])-[#8]-[#1])=[#8])-[#7](-[#1])-[#1])-[#6](=[#8])-[#7]-[#1] <regId=het_65_E(2)>
164. c:2(:c:1:c(:c(:c(:c(:c:1:c(:c(:c:2-[#1])-[#1])-[#1])-[#1])-[#7](-[#1])-[#7](-[#1])-[#6]=[#8])-[#1])-[#1])-[#1] <regId=hzide_naphth(2)>
165. [#6](-[#1])(-[#1])-c:1:c(:c(:c(:c(:c:1-[#8]-[#6](-[#1])-[#1])-[#1])-[#1])-[#6](-[#1])(-[#1])-[#7](-[#1])-[#6;X4])-[#1] <regId=anisol_B(2)>
166. [#6]-1=[#6]-[#7]-[#6](-[#16]-[#6;X4]-1)=[#16] <regId=thio_carbam_ene(2)>
167. [#6](-[#7](-[#6]-[#1])-[#6]-[#1]):[#6]-[#7](-[#1])-[#6](=[#16])-[#6]-[#1] <regId=thio_amide_D(2)>
168. n2nc(c1cccc1c2-[#6])-[#6] <regId=het_65_Da(2)>
169. s:1:c(:c(-[#1]):c(:c:1-[#6](=[#8])-[#7](-[#1])-[#7]-[#1])-[#8]-[#6](-[#1])-[#1])-[#1] <regId=thiophene_D(2)>
170. [#6]-1:[#6]-[#7]=[#6]-[#6](=[#6]-[#7]-[#6])-[#16]-1 <regId=het_thio_6_ene(2)>
171. [#6](-[#1])(-[#1])-[#6](-[#1])(-[#6]#[#7])-[#6](=[#8])-[#6] <regId=cyano_keto_A(2)>
172. c2(c(-[#7](-[#1])-[#1])n(-c:1:c:c:c:c:c:1-[#6](=[#8])-[#8]-[#1])nc2-[#6]=[#8])-[$([#6]#[#7]),$([#6]=[#16])] <regId=anthranil_acid_C(2)>
173. c:2:c:1:c:c:c:c-,:3:c:1:c(:c:c:2)-,:[#7](-,:[#7]=,:[#6]-,:3)-[#1] <regId=naphth_amino_C(2)>
174. c:2:c:1:c:c:c:c-,:3:c:1:c(:c:c:2)-,:[#7]-,:[#7]=,:[#7]-,:3 <regId=naphth_amino_D(2)>
175. c1csc(n1)-[#7]-[#7]-[#16](=[#8])=[#8] <regId=thiazole_amine_G(2)>
176. c:1:c:c:c:2:c(:c:1):n:c(:n:c:2)-[#7](-[#1])-[#6]-3=[#7]-[#6](-[#6]=[#6]-[#7]-3-[#1])(-[#6](-[#1])-[#1])-[#6](-[#1])-[#1] <regId=het_66_B(2)>
177. c:1-,:3:c(:c(:c(:c(:c:1)-[#8]-[#6]-[#1])-[#1])-[#1])-,:c:2:c(:c(:c(:c:2-[#1])-[#1])-[#8]-[#6](-[#1])-[#1])-[#1])-,:[#6](=[#8])-,:[#8]-,:3 <regId=coumarin_A(2)>
178. c:12:c(:c:c:c:n:1)c(c(-[#6](=[#8])~[#8;X1])s2)-[#7](-[#1])-[#1] <regId=anthranil_acid_D(2)>
179. c:1:2:n:c(:c(:n:c:1:[#6]:[#6]:[#6]:[!#1]:2)-[#6](-[#1])=[#6](-[#8]-[#1])-[#6])-[#6](-[#1])=[#6](-[#8]-[#1])-[#6] <regId=het_66_C(2)>
180. c1csc(c1-[#7](-[#1])-[#1])-[#6](-[#1])=[#6](-[#1])-c2cccs2 <regId=thiophene_amino_E(2)>
181. c:2:c:c:1:n:c:3:c(:n:c:1:c:c:2):c:c:c:4:c:3:c:c:c:c:4 <regId=het_6666_A(2)>
182. [#6]:[#6]-[#7](-[#1])-[#16](=[#8])(=[#8])-[#7](-[#1])-[#6]:[#6] <regId=sulfonamide_E(2)>
183. c:1:c:c(:c:c:c:1-[#7](-[#1])-[#1])-[#7](-[#6;X3])-[#6;X3] <regId=anil_di_alk_K(2)>
184. [#7]-2=[#6](-c:1:c:c:c:c:c:1)-[#6](-[#1])(-[#1])-[#6](-[#8]-[#1])(-[#6](-[#9])(-[#9])-[#9])-[#7]-2-[$([#6]:[#6]:[#6]:[#6]:[#6]:[#6]),$([#6](=[#16])-[#6]:[#6]:[#6]:[#6]:[#6]:[#6])] <regId=het_5_C(2)>
185. c:1:c(:c:c:c:c:1)-[#6](=[#8])-[#6](-[#1])=[#6]-,:3-,:[#6](=[#8])-,:[#7](-[#1])-,:[#6](=[#8])-,:[#6](=[#6](-[#1])-c:2:c:c:c:c:c:2)-,:[#7]-,:3-[#1] <regId=ene_six_het_B(2)>
186. [#8]=[#6]-4-[#6]-[#6]-[#6]-3-[#6]-2-[#6](=[#8])-[#6]-[#6]-1-[#6]-[#6]-[#6]-[#6]-1-[#6]-2-[#6]-[#6]-[#6]-3=[#6]-4 <regId=steroid_A(2)>

187. c:1:2:c:3:c(:c(-[#8]-[#1]):c(:c:1:c(:c:n:2-[#6])-[#6]=[#8])-[#1]):n:c:n:3 <regId=het_565_A(2)>
188. [#6;X4]-[#7+](-[#6;X4]-[#8]-[#1])=[#6]-[#16]-[#6]-[#1] <regId=thio_imine_ium(2)>
189. [#6]-3(=[#8])-[#6](=[#6](-[#1])-[#7](-[#1])-c:1:c:c:c:c:c:1-[#6](=[#8])-[#8]-[#1])-[#7]=[#6](-c:2:c:c:c:c:c:2)-[#8]-3 <regId=anthranil_acid_E(2)>
190. c:1(:c(:c(:[c;!H0,$(c-[#6;!H0;!H1])](:o:1))-[#1])-[#1])-[#6;!H0,$([#6]-[#6;!H0;!H1])]=[#7]-[#7](-[#1])-c:2:c:c:n:c:c:2 <regId=hzone_furan_B(2)>
191. c:1(:c(:c(:[c;!H0,$(c-[#6;!H0,!H1])](:s:1))-[#1])-[#1])-[#6;!H0,$([#6]-[#6;!H0;!H1])]-[#6](=[#8])-[#7](-[#1])-c:2:n:c:c:s:2 <regId=thiophene_E(2)>
192. [#6]:[#6]-[#6](-[#1])(-[#1])-[#6](-[#1])(-[#6]=[#8])-[#7]-2-[#6](=[#8])-[#6]-1(-[#1])-[#6](-[#1])(-[#1])-[#6]=[#6]-[#6](-[#1])(-[#1])-[#6]-1(-[#1])-[#6]-2=[#8] <regId=ene_misc_B(2)>
193. [#6]-1(-[#6]=[#8])(-[#6]:[#6])-[#16;X2]-[#6]=[#7]-[#7]-1-[#1] <regId=het_thio_5_B(2)>
194. [#7](-[#1])(-[#1])-c:1:c(:c(:c(:s:1)-[#7](-[#1])-[#6](=[#8])-c:2:c:c:c:c:c:2)-[#6]#[#7])-[#6]:3:[!#1]:[!#1]:[!#1]:[!#1]:[!#1]:3 <regId=thiophene_amino_F(2)>
195. [#6](-[#1])(-[#1])-[#8]-c:1:c(:c(:c(:c(:c:1-[#1])-[#1])-[#6](-[#1])-[#1])-[#1])-[#7](-[#1])-[#6](-[#1])(-[#1])-c:2:c:c:c:c:c:2-[$([#6](-[#1])-[#1]),$([#8]-[#6](-[#1])-[#1])] <regId=anil_OC_alk_D(2)>
196. [#6](-[#1])(-[#1])(-[#1])-[#6](-[#6](-[#1])(-[#1])-[#1])(-[#6](-[#1])(-[#1])-[#1])-c:1:c(:c:c(:c(:c:1-[#1])-[#6](-[#6](-[#1])(-[#1])-[#1])(-[#6](-[#1])(-[#1])-[#1])-[#6](-[#1])(-[#1])-[#1])-[#8]-[#6](-[#1])-[#7])-[#1] <regId=tert_butyl_A(2)>
197. c:1(:c(:o:c:c:1)-[#6]-[#1])-[#6]=[#7]-[#7](-[#1])-[#6](=[#16])-[#7]-[#1] <regId=thio_urea_J(2)>
198. [#7](-[#1])-c1nc(nc2nnc(n12)-[#16]-[#6])-[#7](-[#1])-[#6]<regId=het_thio_65_B(2)>
199. c:1-,:2:c(:c:c:c:c:1-[#6](-[#1])(-[#1])-[#6](-[#1])=[#6](-[#1])-[#1])-,:[#6](=,:[#6](-[#6](=[#8])-[#7](-[#1])-[#6]:[#6])-,:[#6](=[#8])-,:[#8]-,:2)-[#1]<regId=coumarin_B(2)>
200. [#6]-2(=[#16])-[#7]-1-[#6]:[#6]-[#7]=[#7]-[#6]-1=[#7]-[#7]-2-[#1] <regId=thio_urea_K(2)>
201. [#6]:[#6]:[#6]:[#6]:[#6]:[#6]-c:1:c:c(:c(:s:1)-[#7](-[#1])-[#6](=[#8])-[#6])-[#6](=[#8])-[#8]-[#1] <regId=thiophene_amino_G(2)>
202. [#7](-[#1])(-[#1])-c:1:c(:c(:c(:c:c:1-[#7](-[#1])-[#6](-[#1])(-[#6])-[#6](-[#1])-[#6](-[#1])-[#1])-[#1])-[#1])-[#1] <regId=anil_NH_alk_D(2)>
203. [#16]=[#6]-,:2-,:[#7](-[#1])-,:[#7]=,:[#6](-c:1:c(:c(:c(:c(:c:1-[#1])-[#1])-[#8]-[#6](-[#1])-[#1])-[#1])-[#1])-,:[#8]-,:2 <regId=het_thio_5_C(2)>
204. [#16]=[#6]-c:1:c:c:c:2:c:c:c:c:n:1:2 <regId=thio_keto_het(2)>
205. [#6]~1~[#6](~[#7]~[#7]~[#6](~[#6](-[#1])-[#1])~[#6](-[#1])-[#1])~[#7]~[#16]~[#6]~1 <regId=het_thio_N_5B(2)>
206. [#6]-1(-[#6]=,:[#6]-[#6]=,:[#6]-[#6]-1=[!#6&!#1])=[!#6&!#1] <regId=quinone_D(2)>
207. [#6](-[#1])(-[#1])-[#7](-[#6](-[#1])-[#1])-c:1:c(-[#1]):c(:c(:o:1)-[#6](-[#1])=[#6]-[#6]#[#7])-[#1] <regId=anil_di_alk_furan_B(2)>
208. [#8]=[#6]-1-[#6]:[#6]-[#6](-[#1])(-[#1])-[#7]-[#6]-1=[#6]-[#1] <regId=ene_six_het_C(2)>
209. [#6]:[#6]-[#7]:2:[#7]:[#6]:1-[#6](-[#1])(-[#1])-[#16;X2]-[#6](-[#1])(-[#1])-[#6]:1:[#6]:2-[#7](-[#1])-[#6](=[#8])-[#6](-[#1])=[#6]-[#1] <regId=het_55_A(2)>
210. n:1:c(:n(:c:2:c:1:c:c:c:c:2)-[#6](-[#1])-[#1])-[#16]-[#6](-[#1])(-[#1])-[#6](=[#8])-[#7](-[#1])-[#7]=[#6](-[#1])-[#6](-[#1])=[#6]-[#1] <regId=het_thio_65_C(2)>
211. c:1(:c:c(:c(:c:c:1)-[#8]-[#1])-[#6](=!@[#6]-[#7])-[#6]=[#8])-[#8]-[#1] <regId=hydroquin_A(2)>
212. c:1(:c:c(:c(:c:c:1)-[#7](-[#1])-[#6](=[#8])-[#6]:[#6])-[#6](=[#8])-[#8]-[#1])-[#8]-[#1] <regId=anthranil_acid_F(2)>
213. n2(-[#6](-[#1])-[#1])c-1c(-[#6]:[#6]-[#6]-1=[#8])cc2-[#6](-[#1])-[#1] <regId=pyrrole_I(2)>
214. [#6](-[#1])-[#7](-[#1])-c:1:c(:c(:c(:s:1)-[#6]-[#1])-[#6]-[#1])-[#6](=[#8])-[#7](-[#1])-[#6]:[#6] <regId=thiophene_amino_H(2)>
215. [#6]:[#6]-[#7;!R]=[#6]-2-[#6](=[!#6&!#1])-c:1:c:c:c:c:c:1-[#7]-2 <regId=imine_one_fives_C(2)>
216. c:1:c:c:c:c:c:1-[#6](=[#8])-[#7](-[#1])-[#7]=[#6]-3-c:2:c:c:c:c:c:2-c:4:c:c:c:c:c-3:4 <regId=keto_phenone_zone_A(2)>
217. c:1:c(:c:c:c:c:1)-[#7](-[#6](-[#1])-[#1])-[#6](-[#1])=[#6](-[#1])-[#6]=!@[#6](-[#1])-[#6](-[#1])=[#6]-[#6]=@[#7]-c:2:c:c:c:c:c:2 <regId=dyes7A(2)>
218. [#6]:1:2:[!#1]:[#7+](:[!#1]:[#6;!H0,$([#6]-[*])](:[!#1]:1:[#6]:[#6]:[#6]:[#6]:2))~[#6]:[#6] <regId=het_pyridiniums_B(2)>
219. [#7]-2(-c:1:c:c:c:c:c:1)-[#7]=[#6](-[#6](-[#1])-[#1])-[#6](-[#1])(-[#16]-[#6])-[#6]-2=[#8] <regId=het_5_D(2)>
220. c:1:c:c:c(:c:c:1-[#7](-[#1])-c2nc(c(-[#1])s2)-c:3:c:c:c(:c:c:3)-[#6](-[#1])(-[#6]-[#1])-[#6]-[#1])-[#6](=[#8])-[#8]-[#1] <regId=thiazole_amine_H(1)>
221. [#6](-[#1])(-[#1])-[#7](-[#1])-[#6]=[#7]-[#7](-[#1])-c1nc(c(-[#1])s1)-[#6]:[#6]<regId=thiazole_amine_I(1)>
222. [#6]:[#6]-[#7](-[#1])-[#6](=[#8])-c1c(snn1)-[#7](-[#1])-[#6]:[#6] <regId=het_thio_N_5C(1)>
223. [#8]=[#16](=[#8])(-[#6]:[#6])-[#7](-[#1])-c1nc(cs1)-[#6]:[#6] <regId=sulfonamide_F(1)>
224. [#8]=[#16](=[#8])(-[#6]:[#6])-[#7](-[#1])-[#7](-[#1])-c1nc(cs1)-[#6]:[#6] <regId=thiazole_amine_J(1)>
225. s2c:1:n:c:n:c(:c:1c(c2-[#6](-[#1])-[#1])-[#6](-[#1])-[#1])-[#7]-[#7]=[#6]-c3ccco3 <regId=het_65_F(1)>
226. [#6](=[#8])-[#6](-[#1])=[#6](-[#8]-[#1])-[#6](-[#8]-[#1])=[#6](-[#1])-[#6](=[#8])-[#6] <regId=keto_keto_beta_E(1)>
227. c:2(:c:1-[#6](-[#6](-[#6](-c:1:c(:c(:c:c:2-[#1])-[#1])-[#1])(-[#1])-[#1])=[#8])=[#6](-[#6](-[#1])-[#1])-[#6](-[#1])-[#1])-[#1] <regId=ene_five_one_B(1)>

228. [#6]:[#6]-[#7](-[#1])-[#7]=[#6](-[#6](-[#1])-[#1])-[#6](-[#1])(-[#1])-[#6](-[#6](-[#1])-[#1])=[#7]-[#7](-[#1])-[#6]:[#6] <regId=keto_keto_beta_zone(1)>
229. [#6;X4]-[#16;X2]-[#6](=[#7]-[!#1]:[!#1]:[!#1]:[!#1])-[#7](-[#1])-[#7]=[#6] <regId=thio_urea_L(1)>
230. [#6]-1(=[#7]-[#7](-[#6](-[#16]-1)=[#6](-[#1])-[#6]:[#6])-[#6]:[#6])-[#6]=[#8] <regId=het_thio_urea_ene(1)>
231. c:1(:c(:c:2:c(:n:c:1-[#7](-[#1])-[#1]):c:c:c(:c:2-[#7](-[#1])-[#1])-[#6]#[#7])-[#6]#[#7])-[#6]#[#7] <regId=cyano_amino_het_A(1)>
232. [!#1]:1:[!#1]:[!#1]:[!#1](:[!#1]:[!#1]:1)-[#6](-[#1])=[#6](-[#1])-[#6](-[#7](-[#1])-[#7](-[#1])-c2nnnn2-[#6])=[#8] <regId=tetrazole_hzide(1)>
233. c:1:2:c(:c(:c(:c(:c:1:c(:c(:c(:c:2-[#1])-[#1])-[#6](=[#7]-[#6]:[#6])-[#6](-[#1])-[#1])-[#8]-[#1])-[#1])-[#1])-[#1])-[#1] <regId=imine_naphthol_A(1)>
234. c:1(:c(:c:2:c(:c(:c:1-[#8]-[#6](-[#1])-[#1])-[#1]):c(:c(:c(:c:2-[#7](-[#1])-[#6](-[#1])(-[#1])-[#1])-[#1])-c:3:c(:c(:c(:c(:c:3-[#1])-[#1])-[#8]-[#6](-[#1])-[#1])-[#8]-[#6](-[#1])-[#1])-[#1])-[#1])-[#1])-[#8]-[#6](-[#1])-[#1] <regId=misc_anisole_A(1)>
235. c:1:c:c-2:c(:c:c:1)-[#16]-c3c(-[#7]-2)cc(s3)-[#6](-[#1])-[#1] <regId=het_thio_665(1)>
236. c:1:c:c:c-2:c(:c:1)-[#6](-[#6](-[#7]-2-[#6](-[#1])(-[#1])-[#6](-[#1])(-[#1])-[#7]-4-[#6](-c:3:c:c:c:c:c:3-[#6]-4=[#8])=[#8])(-[#1])-[#1])(-[#1])-[#1] <regId=anil_di_alk_L(1)>
237. c:1(:c:c:c(:c:c:1)-[#6]-,:3=,:[#6]-,:[#6](-,:c2cocc2-,:[#6](=,:[#6]-,:3)-[#8]-[#1])=[#8])-[#16]-[#6](-[#1])-[#1] <regId=colchicine_B(1)>
238. [#6;X4]-c:1:c(:c(:c(:c(:c:1-[#1])-[#1])-[#6](=[#8])-[#7](-[#1])-[#6](-[#1])(-[#6](-[#1])(-[#1])-[#6](-[#1])(-[#1])-[#16]-[#6](-[#1])(-[#1])-[#1])-[#6](=[#8])-[#8]-[#1])-[#1])-[#1] <regId=misc_aminoacid_A(1)>
239. n:1:c(:n(:c(:c:1-c:2:c:c:c:c:c:2)-c:3:c:c:c:c:c:3)-[#7]=!@[#6])-[#7](-[#1])-[#1] <regId=imidazole_amino_A(1)>
240. [#6](-c:1:c:c:c(:c:c:1)-[#8]-[#1])(-c:2:c:c:c(:c:c:2)-[#8]-[#1])-[#8]-[#16](=[#8])=[#8] <regId=phenol_sulfite_A(1)>
241. c:2:c:c:1:n:c(:c(:n:c:1:c:c:2)-[#6](-[#1])(-[#1])-[#6](=[#8])-[#6]:[#6])-[#6](-[#1])(-[#1])-[#6](=[#8])-[#6]:[#6] <regId=het_66_D(1)>
242. c:1(:c(:c(:c(:c(:c:1-[#1])-[#8]-[#6](-[#1])-[#1])-[#8]-[#6](-[#1])-[#1])-[#1])-[#1])-[#6](=[#8])-[#6](-[#1])(-[#1])-[#7](-[#6](-[#1])-[#1])-c:2:c:c:c(-[#6](-[#1])-[#1])c:c:2 <regId=misc_anisole_B(1)>
243. [#6](-[#1])(-[#1])-c1nnnn1-c:2:c(:c(:c(:c(:c:2-[#1])-[#1])-[#8]-[#6](-[#1])(-[#1])-[#1])-[#1])-[#1] <regId=tetrazole_A(1)>
244. [#6]-2(=[#7]-c1c(c(nn1-[#6](-[#6]-2(-[#1])-[#1])=[#8])-[#7](-[#1])-[#1])-[#7](-[#1])-[#1])-[#6] <regId=het_65_G(1)>
245. [#6](-[#6]:[#6])(-[#6]:[#6])(-[#6]:[#6])-[#16]-[#6]:[#6]-[#6](=[#8])-[#8]-[#1] <regId=misc_trityl_A(1)>
246. [#8]=[#6](-c:1:c(:c(:n:c(:c:1-[#1])-[#8]-[#6](-[#1])(-[#1])-[#1])-[#8]-[#6](-[#1])(-[#1])-[#1])-[#1])-[#7](-[#1])-[#6](-[#1])(-[#6](-[#1])-[#1])-[#6](-[#1])-[#1] <regId=misc_pyridine_OC(1)>
247. [#7]-1=[#6](-[#7](-[#6](-[#6](-[#6]-1(-[#1])-[#6]:[#6])(-[#1])-[#1])=[#8])-[#1])-[#7]-[#1] <regId=het_6_hydropyridone(1)>
248. [#6]-1(=[#6](-[#6](-[#6](-[#6](-[#6]-1(-[#1])-[#1])(-[#1])-[#6](=[#8])-[#6])(-[#1])-[#6](=[#8])-[#8]-[#1])(-[#1])-[#1])-[#6]:[#6])-[#6]:[#6] <regId=misc_stilbene(1)>
249. [#6](-[#1])(-c:1:c(:c(:c(:c(:c:1-[#1])-[#1])-[Cl])-[#1])-[#1])(-c:2:c(:c(:c(:c(:c:2-[#1])-[#1])-[Cl])-[#1])-[#1])-[#8]-[#6](-[#1])(-[#1])-[#6](-[#1])(-[#1])-[#6](-[#1])(-[#1])-c3nc(c(n3-[#6](-[#1])(-[#1])-[#1])-[#1])-[#1] <regId=misc_imidazole(1)>
250. n:1:c(:c(:c(:c(:c:1-[#1])-[#7](-[#1])-[#1])-[#1])-[#1])-[#7](-[#1])-[#6]:[#6] <regId=anil_NH_no_alk_A(1)>
251. [#7](-[#1])(-c:1:c(:c(:c(:c(:c:1-[#1])-[#1])-[#1])-[#1])-[#8]-[#1])-[#6]-2=[#6](-[#8]-[#6](-[#7]=[#7]-2)=[#7])-[#7](-[#1])-[#1] <regId=het_6_imidate_B(1)>
252. [#7](-[#1])(-c:1:c(:c(:c(:c(:c:1-[#1])-[#1])-[#6](-[#1])-[#1])-[#1])-[#1])-[#6](-[#1])(-[#1])-[#6](-[#1])(-[#1])-c:2:c(:c(:c(:c(:c:2-[#1])-[#1])-[#8]-[#6](-[#1])-[#1])-[#1])-[#1] <regId=anil_alk_B(1)>
253. c:1:c:c-3:c(:c:c:1)-c:2:c:c:c(:c:c:2-[#6]-3=[#6](-[#1])-[#6])-[#7](-[#1])-[#1] <regId=styrene_anil_A(1)>
254. c:1:c:c-2:c(:c:c:1)-[#7](-[#6](-[#8]-[#6]-2)(-[#6](=[#8])-[#8]-[#1])-[#6](-[#1])-[#1])-[#6](=[#8])-[#6](-[#1])-[#1] <regId=misc_aminal_acid(1)>
255. n:1:c(:c(:c(:c(:c:1-[#7](-[#1])-[#1])-[#6](-[#1])-[#1])-[#1])-[#6](-[#1])-[#1])-[#7](-[#1])-[#1] <regId=anil_no_alk_D(1)>
256. [#7](-[#1])(-c:1:c:c:c:c:c:1)-[#6](-[#6])(-[#6])-c:2:c(:c(:c(:c(:c:2-[#1])-[#1])-[#8]-[#6](-[#1])-[#1])-[#1])-[#1] <regId=anil_alk_C(1)>
257. [#7](-[#1])(-c:1:c(:c(:c(:c(:c:1-[#1])-[#1])-[#8]-[#6](-[#1])(-[#1])-[#1])-[#8]-[#6]-[#1])-[#1])-[#6](=[#8])-[#7](-[#1])-[#6](-[#1])(-[#1])-[#6](-[#1])(-[#1])-[#6](-[#1])(-[#1])-[#7](-[#6](-[#1])(-[#1])-[#1])-[#6]:[#6] <regId=misc_anisole_C(1)>
258. c:1-2:c:c-3:c(:c:c:1-[#8]-[#6]-[#8]-2)-[#6]-[#6]-3 <regId=het_465_misc(1)>
259. c:1(:c(:c(:c(:c(:c:1-[#1])-[#1])-[#8]-[#6](-[#1])-[#1])-[#1])-[#6](=[#8])-[#8]-[#1])-[#7](-[#1])-[#6]:[#6] <regId=anthranil_acid_G(1)>
260. c:1(:c:4:c(:n:c(:c:1-[#6](-[#1])(-[#1])-[#7]-3-c:2:c(:c(:c(:c(:c:2-[#6](-[#1])(-[#1])-[#6]-3(-[#1])-[#1])-[#1])-[#1])-[#1])-[#1])-[#1]):c(:c(:c(:c:4-[#1])-[#1])-[#1])-[#1])-[#1] <regId=anil_di_alk_M(1)>
261. c:1:c(:c2:c(:c:c:1)c(c(n2-[#1])-[#6]:[#6])-[#6]:[#6])-[#6](=[#8])-[#8]-[#1] <regId=anthranil_acid_H(1)>
262. [#6]:[#6]-[#7](-[#6](-[#1])-[#1])-[#6](-[#1])(-[#1])-[#6](-[#1])(-[#1])-[#6](-[#1])(-[#1])-[#7](-[#1])-[#6](=[#16])-[#7](-[#1])-c:1:c(:c(:c(:c(:c:1-[F,Cl,Br,I])-[#1])-[#6](-[#1])-[#1])-[#1])-[#1] <regId=thio_urea_M(1)>
263. n:1:c3:c(:c:c2:c:1nc(s2)-[#7])sc(n3)-[#7] <regId=thiazole_amine_K(1)>
264. [#7]=[#6]-1-[#16]-[#6](=[#7])-[#7]=[#6]-1 <regId=het_thio_5_imine_A(1)>
265. c:1:c(:n:c:c:c:1)-[#6](=[#16])-[#7](-[#1])-c:2:c(:c:c:c:c:2)-[#8]-[#6](-[#1])-[#1] <regId=thio_amide_E(1)>

266. c:1-2:c(:c(:c(:c(:c:1-[#6](-c:3:c(-[#16]-[#6]-2(-[#1])-[#1]):c(:c(-[#1]):c(:c:3-[#1])-[#1])-[#1])-[#8]-[#6]:[#6])-[#1])-[#1])-[#1])-[#1] <regId=het_thio_676_B(1)>
267. [#6](-[#1])(-[#1])(-[#1])-c:1:c(:c(:c(:c(:n:1)-[#7](-[#1])-[#16](-c:2:c(:c(:c(:c(:c:2-[#1])-[#1])-[#8]-[#6](-[#1])(-[#1])-[#6](-[#1])(-[#1])-[#6](-[#1])-[#1])-[#1])-[#1])(=[#8])=[#8])-[#1])-[#1])-[#1] <regId=sulfonamide_G(1)>
268. [#6](=[#8])(-[#7]-1-[#6]-[#6]-[#16]-[#6]-[#6]-1)-c:2:c(:c(:c(:c(:c:2-[#16]-[#6](-[#1])-[#1])-[#1])-[#1])-[#1])-[#1] <regId=thio_thiomorph_Z(1)>
269. c:1:c:c:3:c:2:c(:c:1)-[#6](-[#6]=[#6](-c:2:c:c:c:3)-[#8]-[#6](-[#1])-[#1])=[#8] <regId=naphth_ene_one_A(1)>
270. c:1-3:c:2:c(:c(:c:c:1)-[#7]):c:c:c:c:2-[#6](-[#6]=[#6]-3-[#6](-[F])(-[F])-[F])=[#8] <regId=naphth_ene_one_B(1)>
271. c:1:c:c:c:c:2:c:1:c:c:3:c(:n:2):n:c:4:c(:c:3-[#7]):c:c:c:c:4 <regId=amino_acridine_A(1)>
272. c:1:c-3:c(:c:c:c:1)-[#6]-2=[#7]-[!#1]=[#6]-[#6]-[#6]-2-[#6]-3=[#8] <regId=keto_phenone_B(1)>
273. c:1-3:c(:c(:c(:c(:c:1-[#1])-[#1])-[#8]-[#6](-[#1])-[#1])-[#1])-[#6](=[#7]-[#7](-[#1])-c:2:c(:c(:c(:c:2-[#1])-[#1])-[#6](=[#8])-[#8]-[#1])-[#1])-[#1])-c:4:c-3:c(:c(:c(:c:4-[#1])-[#8]-[#6](-[#1])-[#1])-[#1])-[#1] <regId=hzone_acid_A(1)>
274. c:1(:c(:c(:c(:c(:c:1-[#1])-[#1])-[#7](-[#1])-[#1])-[#1])-[#1])-[#16](=[#8])(=[#8])-[#7](-[#1])-c:2:n:n:c(:c(:c:2-[#1])-[#1])-[#1] <regId=sulfonamide_H(1)>
275. c2(c(-[#1])n(-[#6](-[#1])-[#1])c:3:c(:c(:c:1n(c(c(c:1:c2:3)-[#1])-[#1])-[#6](-[#1])-[#1])-[#8]-[#6](-[#1])-[#1])-[#8]-[#6](-[#1])-[#1])-[#1] <regId=het_565_indole(1)>
276. c1(c-2c(c(n1-[#6](-[#8])=[#8])-[#6](-[#1])-[#1])-[#16]-[#6](-[#1])(-[#1])-[#16]-2)-[#6](-[#1])-[#1] <regId=pyrrole_J(1)>
277. s1ccnc1-c2c(n(nc2-[#1])-[#1])-[#7](-[#1])-[#1] <regId=pyrazole_amino_B(1)>
278. c1(c(c(c(n1-[#1])-c:2:c(:c(:c(:c(:c:2-[#1])-[#1])-[#1])-[#1])-[#1])-[#6](-[#1])-[#1])-[#1])-[#6](=[#8])-[#8]-[#1] <regId=pyrrole_K(1)>
279. c:1:2(:c(:c(:c(:o:1)-[#6])-[#1])-[#1])-[#6](=[#8])-[#7](-[#1])-[#6]:[#6](-[#1]):[#6](-[#1]):[#6](-[#1]):[#6](-[#1]):[#6]:2-[#6](=[#8])-[#8]-[#1] <regId=anthranil_acid_I(1)>
280. [!#1]:[#6]-[#6](=[#16])-[#7](-[#1])-[#7](-[#1])-[#6]:[!#1] <regId=thio_amide_F(1)>
281. [#6]-1(=[#8])-[#6](-[#6](-[#6]#[#7])=[#6](-[#1])-[#7])-[#6](-[#7])-[#6]=[#6]-1 <regId=ene_one_C(1)>
282. c2(c-1n(-[#6](-[#6]=[#6]-[#7]-1)=[#8])nc2-c3cccn3)-[#6]#[#7] <regId=het_65_H(1)>
283. [#8]=[#6]-1-[#6](=[#7]-[#7]-[#6]-[#6]-1)-[#6]#[#7] <regId=cyano_imine_D(1)>
284. c:2(:c:1:c:c:c:c:c:1:n:n:c:2)-[#6](-[#6]:[#6])-[#6]#[#7] <regId=cyano_misc_A(1)>
285. c:1:c:c-2:c(:c:c:1)-[#6]=[#6]-[#6](-[#7]-2-[#6](=[#8])-[#7](-[#1])-c:3:c:c(:c(:c:c:3)-[#8]-[#6](-[#1])-[#1])-[#8]-[#6](-[#1])-[#1])(-[#6](-[#1])-[#1])-[#6](-[#1])-[#1] <regId=ene_misc_C(1)>
286. c:2:c:c:1:n:c(:c(:n:c:1:c:c:2)-c:3:c:c:c:c:c:3)-c:4:c:c:c:c:c:4-[#8]-[#1]<regId=het_66_E(1)>
287. [#6](-[#1])(-[#1])-[#6](-[#8]-[#1])=[#6](-[#6](=[#8])-[#6](-[#1])-[#1])-[#6](-[#1])-[#6]#[#6] <regId=keto_keto_beta_F(1)>
288. c:1:c:4:c(:c:c2:c:1nc(n2-[#1])-[#6]-[#8]-[#6](=[#8])-c:3:c:c(:c:c(:c:3)-[#7](-[#1])-[#1])-[#7](-[#1])-[#1]):c:c:c:c:4 <regId=misc_naphthimidazole(1)>
289. c:2(:c:1:c:c:c:c-3:c:1:c(:c:c:2)-[#6]=[#6]-[#6]-3=[#7])-[#7]<regId=naphth_ene_one_C(1)>
290. c:2(:c:1:c:c:c:c:c:1:c-3:c(:c:2)-[#6](-c:4:c:c:c:c:c-3:4)=[#8])-[#8]-[#1] <regId=keto_phenone_C(1)>
291. [#6]-,:2(-,:[#6]=,:[#7]-,:c:1:c:c(:c:c:c:1-,:[#8]-,:2)-[Cl])=[#8] <regId=coumarin_C(1)>
292. [#6]-1=[#6]-[#7](-[#6](-c:2:c-1:c:c:c:c:2)(-[#6]#[#7])-[#6](=[#16])-[#16])-[#6]=[#8] <regId=thio_est_cyano_A(1)>
293. c2(nc:1:c(:c(:c(:c(:c:1-[#1])-[#1])-[#1])-[#1])n2-[#6])-[#7](-[#1])-[#6](-[#7](-[#1])-c:3:c(:c:c:c:c:3-[#1])-[#1])=[#8] <regId=het_65_imidazole(1)>
294. [#7](-[#1])(-[#6]:[#6])-c:1:c(-[#6](=[#8])-[#8]-[#1]):c:c:c(:n:1)-,:[#6]:[#6] <regId=anthranil_acid_J(1)>
295. c:1-3:c(:c:c:c:c:1)-[#16]-[#6](=[#7]-[#7]=[#6]-2-[#6]=[#6]-[#6]=[#6]-[#6]=[#6]-2)-[#7]-3-[#6](-[#1])-[#1] <regId=colchicine_het(1)>
296. c:1-2:c(:c(:c(:c(:c:1-[#1])-[#8]-[#6](-[#1])-[#1])-[#8]-[#6](-[#1])-[#1])-[#1])-[#6](=[#6](-[#6])-[#16]-[#6]-2(-[#1])-[#1])-[#6] <regId=ene_misc_D(1)>
297. c:12:c(:c(:c(:c(:c:1-[#1])-[#1])-[#1])-[#1])c(c(-[#6]:[#6])n2-!@[#6]:[#6])-[#6](-[#1])-[#1] <regId=indole_3yl_alk_B(1)>
298. [#7](-[#1])(-[#1])-c:1:c:c:c(:c:c:1-[#8]-[#1])-[#16](=[#8])(=[#8])-[#8]-[#1] <regId=anil_OH_no_alk_A(1)>
299. s:1:c:c:c(:c:1-[#1])-c:2:c:s:c(:n:2)-[#7](-[#1])-[#1] <regId=thiazole_amine_L(1)>
300. c1c(-[#7](-[#1])-[#1])nnc1-c2c(-[#6](-[#1])-[#1])oc(c2-[#1])-[#1] <regId=pyrazole_amino_A(1)>
301. n1nscc1-c2nc(no2)-[#6]:[#6]<regId=het_thio_N_5D(1)>
302. c:1(:c:c-3:c(:c:c:1)-[#7]-[#6]-4-c:2:c:c:c:c:c:2-[#6]-[#6]-3-4)-[#6;X4] <regId=anil_alk_indane(1)>
303. c:1-2:c(:c(:c(:c(:c:1-[#1])-[#1])-[#1])-[#1])-[#6](=[#6](-[#1])-[#6]-3-[#6](-[#6]#[#7])-[#6](-[#1])(-[#1])-[#6](-[#1])-[#7]-2-3)-[#1] <regId=anil_di_alk_N(1)>
304. c:2-,:3:c(:c:c:1:c:c:c:c:c:1:c:2)-,:[#7](-[#6](-[#1])-[#1])-,:[#6](=[#8])-,:[#6](=,:[#7]-,:3)-[#6]:[#6]-[#7](-[#1])-[#6](-[#1])-[#1] <regId=het_666_C(1)>
305. [#6](-[#8]-[#1]):[#6]-[#6](=[#8])-[#6](-[#1])=[#6](-[#6])-[#6] <regId=ene_one_D(1)>
306. c:1:2:c(:c(:c(:c(:c:1-[#1])-[#1])-[#7](-[#6](-[#1])-[#1])-[#6](-[#1])-[#1])-[#1]):c(:c(-[#1]):n:2-[#1])-[#16](=[#8])=[#8] <regId=anil_di_alk_indol(1)>
307. c:1:2:c(:c(:c(:c(:c:1-[#1])-[#1])-[#7](-[#1])-[#1])-[#1]):c(:c(-[#1]):n:2-[#6](-[#1])-[#1])-[#1] <regId=anil_no_alk_indol_A(1)>

308. [#16;X2]-1-[#6]=[#6](-[#6]#[#7])-[#6](-[#6])(-[#6]=[#8])-[#6](=[#6]-1-[#7](-[#1])-[#1])-[$([#6]=[#8]),$([#6]#[#7])] <regId=dhp_amino_CN_G(1)>
309. [#7]-2-[#6]=[#6](-[#6]=[#8])-[#6](-c:1:c:c:c(:c:c:1)-[#7](-[#6](-[#1])-[#1])-[#6](-[#1])-[#1])-[#6]~3=,:[#6]-2~[#7]~[#6](~[#16])~[#7]~[#6]~3~[#7] <regId=anil_di_alk_dhp(1)>
310. c:1:c(:c:c:c:c:1)-[#6](=[#8])-[#7](-[#1])-c:2:c(:c:c:c:c:2)-[#6](=[#8])-[#7](-[#1])-[#7](-[#1])-c:3:n:c:c:s:3 <regId=anthranil_amide_A(1)>
311. c:1:c:2:c(:c:c:c:1):c(:c:3:c(:c:2):c:c:c:c:3)-[#6]=[#7]-[#7](-[#1])-c:4:c:c:c:c:c:4<regId=hzone_anthran_Z(1)>
312. c:1:c(:c:c:c:c:1)-[#6](-[#1])-[#7]-[#6](=[#8])-[#6](-[#7](-[#1])-[#6](-[#1])-[#1])=[#6](-[#1])-[#6](=[#8])-c:2:c:c:c(:c:c:2)-[#8]-[#6](-[#1])-[#1] <regId=ene_one_amide_A(1)>
313. s:1:c(:c(-[#1]):c(:c:1-[#6]-3=[#7]-c:2:c:c:c:c:c:2-[#6](=[#7]-[#7]-3-[#1])-c:4:c:c:n:c:c:4)-[#1])-[#1] <regId=het_76_A(1)>
314. o:1:c(:c(-[#1]):c(:c:1-[#6](-[#1])(-[#1])-[#7](-[#1])-[#6](=[#16])-[#7](-[#6]-[#1])-[#6](-[#1])(-[#1])-c:2:c:c:c:c:c:2)-[#1])-[#1] <regId=thio_urea_N(1)>
315. c:1:c(:c:c:c:c:1)-[#7](-[#6]-[#1])-[#6](-[#1])-[#6](-[#1])-[#6](-[#1])-[#7](-[#1])-[#6](=[#8])-[#6]-,:2=,:[#6](-,:[#8]-,:[#6](-,:[#6](=,:[#6]-,:2-[#6](-[#1])-[#1])-[#1])=[#8])-[#6](-[#1])-[#1] <regId=anil_di_alk_coum(1)>
316. c2-3:c:c:c:1:c:c:c:c:c:1:c2-[#6](-[#1])-[#6;X4]-[#7]-[#6]-3=[#6](-[#1])-[#6](=[#8])-[#7](-[#6](-[#1])-[#1])-[#6](-[#1])-[#1] <regId=ene_one_amide_B(1)>
317. c:1:c(:c:c:c:c:1)-[#6]-4=[#7]-[#7]:2:[#6](:[#7+]:c:3:c:2:c:c:c:c:3)-[#16]-[#6;X4]-4 <regId=het_thio_656c(1)>
318. [#6]-2(=[#8])-[#6](=[#6](-[#6](-[#1])-[#1])-[#7](-[#1])-[#6](-[#1])(-[#1])-[#6](-[#1])(-[#1])-[#6](-[#1])-[#1])-[#7]=[#6](-c:1:c:c:c:c:c:1)-[#8]-2 <regId=het_5_ene(1)>
319. c:1:c(:c:c:c:c:1)-[#7]-2-[#6](=[#8])-[#6](=[#6](-[#1])-[#6]-2=[#8])-[#16]-c:3:c:c:c:c:c:3 <regId=thio_imide_A(1)>
320. [#7]-,:1(-[#1])-,:[#7]=,:[#6](-[#7]-[#1])-,:[#16]-,:[#6](=,:[#6]-,:1-,:[#6]:[#6])-,:[#6]:[#6] <regId=dhp_amidine_A(1)>
321. c:1(:c(:c-3:c(:c(:c:1-[#7](-[#1])-[#6](=[#16])-[#7](-[#1])-[#6](-[#1])-c:2:c(:c(:c(:o:2)-[#6]-[#1])-[#1])-[#1])-[#1])-[#8]-[#6](-[#8]-3)(-[#1])-[#1])-[#1])-[#1] <regId=thio_urea_O(1)>
322. c:1(:c(:c(:c(:c(:c:1-[#7](-[#1])-[#6](=[#16])-[#7](-[#1])-c:2:c:c:c:c:c:2)-[#1])-[#7](-[#6](-[#1])-[#1])-[#6](-[#1])-[#1])-[#1])-[#1])-[#1] <regId=anil_di_alk_O(1)>
323. [#8]=[#6]-!@n:1:c:c:c-,:2:c:1-,:[#7](-[#1])-,:[#6](=[#16])-,:[#7]-,:2-[#1] <regId=thio_urea_P(1)>
324. [#6](-[F])(-[F])-[#6](=[#8])-[#7](-[#1])-c:1:c(-[#1]):n(-[#6](-[#1])(-[#1])-[#6](-[#1])(-[#1])-[#8]-[#6](-[#1])(-[#1])-[#6]:[#6]):n:c:1-[#1] <regId=het_pyraz_misc(1)>
325. [#7]-2=[#7]-[#6]:1:[#7]:[!#6&!#1]:[#7]:[#6]:1-[#7]=[#7]-[#6]:[#6]-2 <regId=diazox_C(1)>
326. [#6]-2(-[#1])(-[#8]-[#1])-[#6]:1:[#7]:[!#6&!#1]:[#7]:[#6]:1-[#6](-[#1])(-[#8]-[#1])-[#6]=[#6]-2 <regId=diazox_D(1)>
327. [#6]-1(-[#6](-[#1])(-[#1])-[#6]-1(-[#1])-[#1])(-[#6](=[#8])-[#7](-[#1])-c:2:c:c:c(:c:c:2)-[#8]-[#6](-[#1])(-[#1])-[#8])-[#16](=[#8])(=[#8])-[#6]:[#6] <regId=misc_cyclopropane(1)>
328. [#6]-1:[#6]-[#6](=[#8])-[#6]=[#6]-1-[#7]=[#6](-[#1])-[#7](-[#6;X4])-[#6;X4] <regId=imine_ene_one_B(1)>
329. c:1:c:c(:c:c-,:2:c:1-,:[#6](=,:[#6](-[#1])-,:[#6](=[#8])-,:[#8]-,:2)-c:3:c:c:c:c:c:3)-[#8]-[#6](-[#1])(-[#1])-[#6]:[#8]:[#6] <regId=coumarin_D(1)>
330. c:1:c(:o:c(:c:1-[#6](-[#1])-[#1])-[#6](-[#1])-[#1])-[#6](-[#1])(-[#1])-[#7]-[#6](-[#1])(-[#1])-[#6](-[#1])(-[#8]-[#6](-[#1])-[#1])-[#6](-[#1])(-[#1])-[#8]-c:2:c:c-3:c(:c:c:2)-[#8]-[#6](-[#8]-3)(-[#1])-[#1] <regId=misc_furan_A(1)>
331. [#7]-4(-c:1:c:c:c:c:c:1)-[#6](=[#8])-[#16]-[#6](-[#1])(-[#7](-[#1])-c:2:c:c:c:c:3:c:c:c:c:c:2:3)-[#6]-4=[#8] <regId=rhod_sat_E(1)>
332. [#7]-3(-[#6](=[#8])-c:1:c:c:c:c:c:1)-[#6](=[#7]-c:2:c:c:c:c:c:2)-[#16]-[#6](-[#1])(-[#1])-[#6]-3=[#8] <regId=rhod_sat_imine_A(1)>
333. [#7]-2(-c:1:c:c:c:c:c:1)-[#6](=[#8])-[#16]-[#6](-[#1])(-[#1])-[#6]-2=[#16] <regId=rhod_sat_F(1)>
334. [#7]-1(-[#6](-[#1])-[#1])-[#6](=[#16])-[#7](-[#6]:[#6])-[#6](=[#7]-[#6]:[#6])-[#6]-1=[#7]-[#6]:[#6] <regId=het_thio_5_imine_B(1)>
335. [#16]-1-[#6](=[#7]-[#7]-[#1])-[#16]-[#6](=[#7]-[#6]:[#6])-[#6]-1=[#7]-[#6]:[#6] <regId=het_thio_5_imine_C(1)>
336. [#6]-2(=[#8])-[#6](=[#6](-[#1])-c:1:c(:c:c:c(:c:1)-[F,Cl,Br,I])-[#8]-[#6](-[#1])-[#1])-[#7]=[#6](-[#16]-[#6](-[#1])-[#1])-[#16]-2 <regId=ene_five_het_N(1)>
337. [#6](-[#1])(-[#1])-[#16]-[#6](=[#16])-[#7](-[#1])-[#6](-[#1])(-[#1])-[#6]:[#6] <regId=thio_carbam_A(1)>
338. c:1(:c(:c(:c(:c(:c:1-[#1])-[#1])-[#6](-[#1])-[#1])-[#7](-[#1])-[#6](=[#8])-[#6](-[#1])(-[#1])-[#6](-[#1])(-[#1])-[#6]:[#6])-[#1])-[#7](-[#1])-[#6](=[#8])-[#6](-[#1])(-[#1])-[#6](-[#1])(-[#1])-[#6]:[#6] <regId=misc_anilide_A(1)>
339. c:1(:c(:c(:c(:c(:c:1-[#6](-[#1])-[#1])-[#1])-[Br])-[#1])-[#6](-[#1])-[#1])-[#7](-[#1])-[#6](=[#8])-[#7](-[#1])-[#6](-[#1])(-[#1])-[#6](-[#1])(-[#1])-[#6](-[#1])-[#1] <regId=misc_anilide_B(1)>
340. c:1-2:c(:c:c:c(:c:1-[#8]-[#6](-[#1])(-[#1])-[#7](-[#6]:[#6]-[#8]-[#6](-[#1])-[#1])-[#6]-2(-[#1])-[#1])-[#1])-[#1] <regId=mannich_B(1)>
341. c:1-2:c(:c(:c(:c(:c:1-[#8]-[#6](-[#1])(-[#1])-[#7](-[#6](-[#1])-[#1])-[#6]-2(-[#1])-[#1])-[#1])-[#8])-[#8])-[#1] <regId=mannich_catechol_A(1)>
342. [#7](-[#1])(-c:1:c(:c(:c(:c(:c:1-[#1])-[#1])-[#6](-[#1])(-[#6](-[#1])-[#1])-[#6](-[#1])-[#1])-[#1])-[#1])-[#6](-[#1])(-[#1])-[#6](-[#1])(-[#1])-[#7](-[#6](-[#1])-[#1])-[#6](-[#1])-[#1] <regId=anil_alk_D(1)>
343. n:1:2:c:c:c(:c:c:1:c:c(:c:2-[#6](=[#8])-[#6]:[#6])-[#6]:[#6])-[#6](~[#8])~[#8] <regId=het_65_I(1)>
344. c:1(:c(:c(:c(:c(:c:1-[#1])-[#1])-[#1])-[#6](=[#6](-[#1])-[#1])-[#6](-[#1])-[#1])-[#1])-[#6](-[#6;X4])(-[#6;X4])-[#7](-[#1])-[#6](=[#8])-[#7](-[#6](-[#1])(-[#1])-[#6](-[#1])-[#1])-[#6](-[#1])(-[#1])-[#6](-[#1])(-[#1])-[#6](-[#1])-[#6](-[#1])(-[#1])-[#6]:[#6] <regId=misc_urea_A(1)>

345. [#6]-3(-[#1])(-n:1:c(:n:c(:c:1-[#1])-[#1])-[#1])-c:2:c(:c(:c(:c(:c:2-[#1])-[Br])-[#1])-[#1])-[#6](-[#1])(-[#1])-[#6](-[#1])(-[#1])-c:4:c-3:c(:c(:c(:c:4-[#1])-[#1])-[#1])-[#1] <regId=imidazole_C(1)>
346. [#6](=[#6](-[#1])-[#6](-[#1])(-[#1])-n:1:c(:n:c(:c:1-[#1])-[#1])-[#1])(-[#6]:[#6])-[#6]:[#6] <regId=styrene_imidazole_A(1)>
347. c:1(:n:c(:c(-[#1]):s:1)-c:2:c:c:n:c:c:2)-[#7](-[#1])-[#6]:[#6]-[#6](-[#1])-[#1] <regId=thiazole_amine_M(1)>
348. c:1(:n:c(:c(-[#1]):s:1)-c:2:c:c:c:c:c:2)-[#6](-[#1])(-[#6](-[#1])-[#1])-[#6](-[#1])(-[#1])-[#6](-[#1])(-[#1])-[#7]-[#6](-[#1])(-[#1])-c:3:c:c:c:n:3-[#1] <regId=misc_pyrrole_thiaz(1)>
349. n:1(-[#1]):c(:c(-[#6](-[#1])-[#1]):c(:c:1-[#6](-[#1])(-[#1])-[#6](-[#1])-[#1])-[#6](-[#1])(-[#1])-[#6](-[#1])-[#1])-[#6](=[#8])-[#8]-[#6](-[#1])-[#1] <regId=pyrrole_L(1)>
350. c:2(:n:c:1:c(:c(:c:c(:c:1-[#1])-[F,Cl,Br,I])-[#1]):n:2-[#1])-[#16]-[#6](-[#1])(-[#1])-[#6](=[#8])-[#7](-[#1])-[#6]:[#6] <regId=het_thio_65_D(1)>
351. c:1(:c(:c-2:c(:c(:c:1-[#8]-[#6](-[#1])-[#1])-[#1])-[#6]=[#6]-[#6](-[#1])-[#16]-2)-[#1])-[#8]-[#6](-[#1])-[#1] <regId=ene_misc_E(1)>
352. [#7]-1(-[#1])-[#6](=[#16])-[#6](-[#1])(-[#6]#[#7])-[#6](-[#1])(-[#6]:[#6])-[#6](=[#6]-1-[#6]:[#6])-[#1] <regId=thio_cyano_A(1)>
353. n:1:c(:c(:c(:c(:c:1-[#16;X2]-c:2:c:c:c:c:c:2-[#7](-[#1])-[#1])-[#6]#[#7])-c:3:c:c:c:c:c:3)-[#6]#[#7])-[#7](-[#1])-[#1] <regId=cyano_amino_het_B(1)>
354. [#7]-,:2(-c:1:c:c:c(:c:c:1)-[#8]-[#6](-[#1])-[#1])-,:[#6](=[#8])-,:[#6](=,:[#6]-,:[#6](=,:[#7]-,:2)-n:3:c:n:c:c:3)-[#6]#[#7]<regId=cyano_pyridone_G(1)>
355. o:1:c(:c:c:2:c:1:c(:c(:c(:c:2-[#1])-[#8]-[#6](-[#1])-[#1])-[#8]-[#6](-[#1])-[#1])-[#1])-[#6](~[#8])~[#8] <regId=het_65_J(1)>
356. [#6]#[#6]-[#6](=[#8])-[#6]#[#6] <regId=ene_one_yne_A(1)>
357. c:2(:c:1:c(:c(:c(:c(:c:1:c(:c(:c:2-[#8]-[#1])-[#6]=[#8])-[#1])-[#1])-[#1])-[#1])-[#1])-[#7](-[#1])-[#1] <regId=anil_OH_no_alk_B(1)>
358. c:1(:c(:c(:[c;!H0,$(c-[#6;!H0;!H1])](:o:1))-[#1])-[#1])-[#6](=[#8])-[#7](-[#1])-[#7]=[#6;!H0,$([#6]-[#6;!H0!H1])]-c:2:c:c:c:c(:c:2)-[*]-[*]-[*]-c:3:c:c:c:o:3 <regId=hzone_acyl_misc_A(1)>
359. [#16](=[#8])(=[#8])-[#7](-[#1])-c:1:c(:c(:c(:s:1)-[#6]-[#1])-[#6]-[#1])-[#6](=[#8])-[#7]-[#1] <regId=thiophene_F(1)>
360. [#6](-[#1])(-[#1])-[#8]-c:1:c(:c(:c(:c(:c:1-[#1])-[#1])-[#1])-[#1])-[#7](-[#1])-[#6](-[#1])(-[#1])-[#6](-[#1])(-[#8]-[#1])-[#6](-[#1])-[#1] <regId=anil_OC_alk_E(1)>
361. [#6](-[#1])(-[#1])-[#8]-c:1:c(:c(:c(:c(:c:1-[#1])-[#1])-[#1])-[#1])-[#7](-[#1])-[#6](-[#1])(-[#6]=[#8])-[#16] <regId=anil_OC_alk_F(1)>
362. n1nnnc2cccc12 <regId=het_65_K(1)>
363. c:1-,:2:c(-[#1]):s:c(:c:1-,:[#6](=[#8])-,:[#7]-,:[#7]=,:[#6]-,:2-[#7](-[#1])-[#1])-[#6]=[#8] <regId=het_65_L(1)>
364. c:1-,:3:c(:c:2:c(:c:c:1-[Br]):o:c:c:2)-,:[#6](=,:[#6]-,:[#6](=[#8])-,:[#8]-,:3)-[#1]<regId=coumarin_E(1)>
365. c:1-,:3:c(:c:c:c:c:1)-,:[#6](=,:[#6](-[#6](=[#8])-[#7](-[#1])-c:2:n:o:c:c:2-[Br])-,:[#6](=[#8])-,:[#8]-,:3)-[#1] <regId=coumarin_F(1)>
366. c:1-,:2:c(:c:c(:c:c:1-[F,Cl,Br,I])-[F,Cl,Br,I])-,:[#6](=,:[#6](-[#6](=[#8])-[#7](-[#1])-[#1])-,:[#6](=[#7]-[#1])-,:[#8]-,:2)-[#1] <regId=coumarin_G(1)>
367. c:1-,:3:c(:c:c:c:c:1)-,:[#6](=,:[#6](-[#6](=[#8])-[#7](-[#1])-c:2:n:c(:c:s:2)-[#6]:[#16]:[#6]-[#1])-,:[#6](=[#8])-,:[#8]-,:3)-[#1] <regId=coumarin_H(1)>
368. [#6](-[#1])(-[#1])-[#16;X2]-c:2:n:n:c:1-[#6]:[#6]-[#7]=[#6]-[#8]-c:1:n:2 <regId=het_thio_67_A(1)>
369. [#16](=[#8])(=[#8])(-c:1:c:n(-[#6](-[#1])-[#1]):c:n:1)-[#7](-[#1])-c:2:c:n(:n:c:2)-[#6](-[#1])(-[#1])-[#6]:[#6]-[#8]-[#6](-[#1])-[#1] <regId=sulfonamide_I(1)>
370. c:1-2:c(:c(:c(:c(:c:1-[#8]-[#6](-[#1])(-[#1])-[#8]-2)-[#6](-[#1])(-[#1])-[#7]-3-[#6](-[#1])(-[#1])-[#6](-[#1])(-[#1])-[#6]:[#6]-3)-[#1])-[#1])-[#1] <regId=het_65_mannich(1)>
371. [#6](-[#1])(-[#1])-[#8]-[#6]:[#6]-[#6](-[#1])(-[#1])-[#7](-[#1])-c:2:c(:c(:c:1:n(:c(:n:c:1:c:2-[#1])-[#1])-[#6]-[#1])-[#1])-[#1] <regId=anil_alk_A(1)>
372. [#7]-4(-c:1:c:c:c:c:c:1)-[#6](=[#7+](-c:2:c:c:c:c:c:2)-[#6](=[#7]-c:3:c:c:c:c:c:3)-[#7]-4)-[#1] <regId=het_5_inium(1)>
373. [#6](-[#1])(-[#1])-[#7](-[#6](-[#1])-[#1])-c:2:c:c:c:1:s:c(:n:c:1:c:2)-[#16]-[#6](-[#1])-[#1] <regId=anil_di_alk_P(1)>
374. c:1:2:c(:c(:c(:c(:c:1:c(:c(-[#1]):c(:c:2-[#1])-[#1])-[#6](-[#6](-[#1])-[#1])=[#7]-[#7](-[#1])-[#6](=[#16])-[#7](-[#1])-[#6]:[#6]:[#6])-[#1])-[#1])-[#1])-[#1] <regId=thio_urea_Q(1)>
375. [#6]:1(:[#7]:[#6](:[#7]:[!#1]:[#7]:1)-c:2:c(:c(:c(:o:2)-[#1])-[#1])-[#1])-[#16]-[#6;X4] <regId=thio_pyridine_A(1)>
376. n:1:c(:n:c(:n:c:1-[#7](-[#6](-[#1])-[#1])-[#6](-[#1])-[#1])-[#7](-[#6](-[#1])-[#1])-[#6](-[#1])-[#1])-[#7](-[#6]-[#1])-[#6]=[#8] <regId=melamine_B(1)>
377. c:1(:n:s:c(:n:1)-[#7](-[#6](-[#1])-[#1])-[#6](-[#1])(-[#1])-[#6](-[#1])(-[#1])-[#7]-[#6](=[#8])-c:2:c:c:c:c:c:2-[#6](=[#8])-[#8]-[#1])-c:3:c:c:c:c:c:3 <regId=misc_phthal_thio_N(1)>
378. n:1:c(:c(:c(:c(:c:1-[#1])-[#1])-[#1])-[#1])-[#6](=[#8])-[#7](-[#1])-[#7]=[#6](-[#1])-c:2:c:c:c:c:c:2-[#8]-[#6](-[#1])(-[#1])-[#6](=[#8])-[#8]-[#1] <regId=hzone_acyl_misc_B(1)>
379. [#6](-[#1])(-[#1])(-[#1])-[#6](-[#6](-[#1])(-[#1])-[#1])(-[#6](-[#1])(-[#1])-[#1])-c:1:c(:c(:c(:c(:c:1-[#8]-[#1])-[#6](-[#6](-[#1])(-[#1])-[#1])(-[#6](-[#1])(-[#1])-[#1])-[#6](-[#1])(-[#1])-[#1])-[#1])-[#6](-[#1])(-[#1])-c:2:c:c:c(:c(:c:2-[#1])-[#1])-[#8]-[#1])-[#1] <regId=tert_butyl_B(1)>
380. [#7](-[#1])(-[#1])-c:1:c(-[#7](-[#1])-[#1]):c(:c(-[#1]):c:2:n:o:n:c:1:2)-[#1] <regId=diazox_E(1)>

381. [#7](-[#1])(-[#1])-c:1:c(:c(:c(:c(:c:1-[#7](-[#1])-[#16](=[#8])=[#8])-[#1])-[#7](-[#1])-[#6](-[#1])-[#1])-[F,Cl,Br,I])-[#1] <regId=anil_NH_no_alk_B(1)>
382. [#7](-[#1])(-[#1])-c:1:c(:c(:c(:c(:c:1-[#7]=[#6]-2-[#6](=[#6]~[#6]~[#6]=[#6]-2)-[#1])-[#1])-[#1])-[#1])-[#1] <regId=anil_no_alk_A(1)>
383. [#7](-[#1])(-[#1])-c:1:c(:c(:c(:c(:c:1-n:2:c:c:c:c:2)-[#1])-[#6](-[#1])-[#1])-[#6](-[#1])-[#1])-[#1] <regId=anil_no_alk_B(1)>
384. [#16]=[#6]-[#6](-[#6](-[#1])-[#1])=[#6](-[#6](-[#1])-[#1])-[#7](-[#6](-[#1])-[#1])-[#6](-[#1])-[#1] <regId=thio_ene_amine_A(1)>
385. [#6]-1:[#6]-[#8]-[#6]-2-[#6](-[#1])(-[#1])-[#6](=[#8])-[#8]-[#6]-1-2 <regId=het_55_B(1)>
386. [#8]-[#6](=[#8])-[#6](-[#1])(-[#1])-[#16;X2]-[#6](=[#7]-[#6]#[#7])-[#7](-[#1])-c:1:c:c:c:c:c:1 <regId=cyanamide_A(1)>
387. [#8]=[#6]-[#6]-1=[#6](-[#16]-[#6](=[#6](-[#1])-[#6])-[#16]-1)-[#6]=[#8] <regId=ene_one_one_A(1)>
388. [#8]=[#6]-1-[#7]-[#7]-[#6](=[#7]-[#6]-1=[#6]-[#1])-[!#1]:[!#1] <regId=ene_six_het_D(1)>
389. [#8]=[#6]-[#6](-[#1])=[#6](-[#6]#[#7])-[#6] <regId=ene_cyano_E(1)>
390. [#8](-[#1])-[#6](=[#8])-c:1:c(:c(:c(:c(:c:1-[#8]-[#1])-[#1])-c:2:c(-[#1]):c(:c(:o:2)-[#6](-[#1])=[#6](-[#6]#[#7])-c:3:n:c:c:n:3)-[#1])-[#1])-[#1] <regId=ene_cyano_F(1)>
391. c:1:c(:c:c:c:c:1)-[#7](-c:2:c:c:c:c:c:2)-[#7]=[#6](-[#1])-[#6]:3:[#6](:[#6](:[#6](:[!#1]:3)-c:4:c:c:c:c(:c:4)-[#6](=[#8])-[#8]-[#1])-[#1])-[#1] <regId=hzone_furan_C(1)>
392. [#7](-[#1])(-[#1])-c:1:c(:c(:c(:c(:c:1-[#1])-[#1])-c:2:c(-[#1]):c(:c(-[#6](-[#1])-[#1]):o:2)-[#6]=[#8])-[#1])-[#1] <regId=anil_no_alk_C(1)>
393. [#8](-[#1])-[#6](=[#8])-c:1:c:c:c(:c:c:1)-[#7]-[#7]=[#6](-[#1])-[#6]:2:[#6](:[#6](:[#6](:[!#1]:2)-c:3:c:c:c:c:c:3)-[#1])-[#1] <regId=hzone_acid_D(1)>
394. [#8](-[#1])-[#6](=[#8])-c:1:c:c:c:c(:c:1)-[#6]:[!#1]:[#6]-[#6]=[#7]-[#7](-[#1])-[#6](=[#8])-[#6](-[#1])(-[#1])-[#8] <regId=hzone_furan_E(1)>
395. [#8](-[#1])-[#6]:1:[#6](:[#6]:[!#1]:[#6](:[#7]:1)-[#7](-[#1])-[#1])-[#6](-[#1])(-[#1])-[#6](=[#8])-[#8] <regId=het_6_pyridone_NH2(1)>
396. [#6]-1(=[!#6&!#1])-[#6](-[#7]=[#6]-[#16]-1)=[#8] <regId=imine_one_fives_D(1)>
397. n2(-c:1:c:c:c:c:c:1)c(c(-[#1])c(c2-[#6]=[#7]-[#8]-[#1])-[#1])-[#1] <regId=pyrrole_M(1)>
398. n2(-[#6](-[#1])-c:1:c(:c(:c:c(:c:1-[#1])-[#1])-[#1])-[#1])c(c(-[#1])c(c2-[#6]-[#1])-[#1])-[#6]-[#1] <regId=pyrrole_N(1)>
399. n1(-[#6](-[#1])-[#1])c(c(-[#6](=[#8])-[#6])c(c1-[#6]:[#6])-[#6])-[#6](-[#1])-[#1] <regId=pyrrole_O(1)>
400. n1(-[#6])c(c(-[#1])c(c1-[#6](-[#1])=[#6](-[#6]#[#7])-c:2:n:c:c:s:2)-[#1])-[#1] <regId=ene_cyano_G(1)>
401. n3(-c:1:c:c:c:c:c:1-[#7](-[#1])-[#16](=[#8])(=[#8])-c:2:c:c:c:s:2)c(c(-[#1])c(c3-[#1])-[#1])-[#1] <regId=sulfonamide_J(1)>
402. n2(-c:1:c(:c(:c(:c(:c:1-[#1])-[#1])-[#1])-[#1])-[#6](=[#8])-[#7](-[#1])-[#6](-[#1])(-[#6](-[#1])-[#1])-[#6](-[#1])(-[#1])-[#8]-[#6]:[#6])c(c(-[#1])c(c2-[#1])-[#1])-[#1] <regId=misc_pyrrole_benz(1)>
403. c:1(:c:c:c:c:c:1)-[#7](-[#1])-[#6](=[#16])-[#7]-[#7](-[#1])-[#6](-[#1])=[#6](-[#1])-[#6]=[#8] <regId=thio_urea_R(1)>
404. [#6]-1(-[#6](=[#8])-[#6](-[#1])(-[#1])-[#6]-[#6](-[#1])(-[#1])-[#6]-1=[#8])=[#6](-[#7]-[#1])-[#6]=[#8] <regId=ene_one_one_B(1)>
405. [#7](-[#1])(-[#1])-[#6]-1=[#6](-[#6]#[#7])-[#6](-[#1])(-[#6]:[#6])-[#16]-[#6;X4]-[#16]-1 <regId=dhp_amino_CN_H(1)>
406. [#6](-[#1])(-[#1])-[#8]-c:1:c(:c(:c(:c(:c:1-[#1])-[#1])-[#1])-[#1])-[#7](-[#1])-c:2:c:c:n:c:3:c(:c:c:c(:c:2:3)-[#8]-[#6](-[#1])-[#1])-[#8]-[#6](-[#1])-[#1] <regId=het_66_anisole(1)>
407. [#6](-[#1])(-[#1])-[#8]-c:1:c(:c(:c(:c(:c:1-[#1])-[#1])-[#8]-[#6](-[#1])-[#1])-[#1])-[#7](-[#1])-c:2:n:c(:c:s:2)-c:3:c:c:c(:c:c:3)-[#8]-[#6](-[#1])-[#1] <regId=thiazole_amine_N(1)>
408. [#6]~1~3~[#7](-[#6]:[#6])~[#6]~[#6]~[#6]~[#6]~1~[#6]~2~[#7]~[#6]~[#6]~[#6]~[#7+]~2~[#7]~3 <regId=het_pyridiniums_C(1)>
409. [#7]-3(-c:2:c:1:c:c:c:c:c:1:c:c:c:2)-[#7]=[#6](-[#6](-[#1])-[#1])-[#6](-[#1])(-[#1])-[#6]-3=[#8] <regId=het_5_E(1)>
410. [#6]-1(=[#6;!H0,$([#6]-[#6;!H0;!H1]),$([#6]-[#6]=[#8])]-[#16]-[#6](-[#7;!H0,$([#7]-[#6;!H0]),$([#7]-[#6]:[#6])]-1)=[#7;!R])-[$([#6](-[#1])-[#1]),$([#6]:[#6])] <regId=thiaz_ene_A(128)>
411. n2(-[#6]:1:[!#1]:[#6]:[#6]:[#6]:[#6]:1)c(cc(c2-[#6;X4])-[#1])-[#6;X4] <regId=pyrrole_A(118)>
412. c:1:c:c(:c(:c:c:1)-[#8]-[#1])-[#8]-[#1] <regId=catechol_A(92)>
413. [#6]-1(=[#6])-[#6](-[#7]=[#6]-[#16]-1)=[#8] <regId=ene_five_het_B(90)>
414. [#6]-1=[!#1]-[!#6&!#1]-[#6](-[#6]-1=[!#6&!#1;!R])=[#8] <regId=imine_one_fives(89)>
415. [#6]-1(-[#6](-[#6]=[#6]-[!#6&!#1]-1)=[#6])=[!#6&!#1] <regId=ene_five_het_C(85)>
416. [#6]-[#7]-1-[#6](-[#1])(-[#1])-[#6](-[#1])(-[#1])-[#7](-[#6](-[#1])(-[#1])-[#6]-1(-[#1])-[#1])-[#7]=[#6](-[#1])-[#6]:[!#1] <regId=hzone_pipzn(79)>
417. c:1-2:c(:c:c:c:c:1)-[#6](=[#8])-[#6;X4]-[#6]-2=[#8] <regId=keto_keto_beta_A(68)>
418. n1(-[#6])c(c(-[#1])c(c1-[#6]=[#7]-[#7])-[#1])-[#1] <regId=hzone_pyrrol(64)>
419. [#6]=!@[#6](-[!#1])-@[#6](=!@[!#6&!#1])-@[#6](=!@[#6])-[!#1] <regId=ene_one_ene_A(57)>
420. [#6](-[#6]#[#7])(-[#6]#[#7])-[#6](-[#7](-[#1])-[#1])=[#6]-[#6]#[#7] <regId=cyano_ene_amine_A(56)>
421. c:1-2:c(:c:c:c:c:1)-[#6](=[#8])-[#6](=[#6])-[#6]-2=[#8] <regId=ene_five_one_A(55)>
422. [#6]-,:1(=,:[!#1]-,:[!#1]=,:[!#1]-,:[#7](-,:[#6]-,:1=[#16])-[#1])-[#6]#[#7] <regId=cyano_pyridone_A(54)>
423. c:1:c:c-2:c(:c:c:1)-[#6]-3-[#6](-[#6]-[#7]-2)-[#6]-[#6]=[#6]-3 <regId=anil_alk_ene(51)>
424. c:1:c:2:c(:c:c:c:1):n:c:3:c(:c:2-[#7]):c:c:c:c:3 <regId=amino_acridine_A(46)>

425. [#6]-1(=[#6])-[#6](=[#8])-[#7]-[#7]-[#6]-1=[#8] <regId=ene_five_het_D(46)>
426. [#7](-[#1])(-[#1])-c:1:c(:c(:c(:s:1)-[!#1])-[!#1])-[#6]=[#8] <regId=thiophene_amino_Aa(45)>
427. [#7]-[#6]=!@[#6]-2-[#6](=[#8])-c:1:c:c:c:c:c:1-[!#6&!#1]-2 <regId=ene_five_het_E(44)>
428. c:1(:c(:c(:c(:c(:c:1-[#8]-[#1])-[F,Cl,Br,I])-[#1])-[F,Cl,Br,I])-[#1])-[#16](=[#8])(=[#8])-[#7]
<regId=sulfonamide_A(43)>
429. [#6]-[#6](=[#16])-[#6] <regId=thio_ketone(43)>
430. c:1:c:c(:c:c:c:1-[#8]-[#1])-[#7](-[#1])-[#16](=[#8])=[#8] <regId=sulfonamide_B(41)>
431. c:1(:c(:c(:c(:c(:c:1-[#1])-[#1])-[$([#8]),$([#7]),$([#6](-[#1])-[#1])])-[#1])-[#1])-[#7](-[#1])-[#1]
<regId=anil_no_alk(40)>
432. [c;!H0,$(c-[#6](-[#1])-[#1]),$(c-[#6]:[#6])]:1:c(:c(:c(:s:1)-[#7](-[#1])-[#6](=[#8])-[#6])-[#6](=[#8])-[#8])-[$([#6]:1:[#6]:[#6]:[#6]:[#6]:[#6]:1),$([#6]:1:[#16]:[#6]:[#6]:[#6]:1)] <regId=thiophene_amino_Ab(40)>
433. [#7+]:1(:[#6]:[#6]:[!#1]:c:2:c:1:c(:[c;!H0,$(c-[#7])]:c:c:2)-[#1])-[$([#6](-[#1])(-[#1])-[#1]),$([#8;X1]),$([#6](-[#1])(-[#1])-[#6](-[#1])=[#6](-[#1])-[#1]),$([#6](-[#1])(-[#1])-[#6](-[#1])(-[#1])-[#8]-[#1]),$([#6](-[#1])(-[#1])-[#6](=[#8])-[#6]),$([#6](-[#1])(-[#1])-[#6](=[#8])-[#7](-[#1])-[#6]:[#6]),$([#6](-[#1])(-[#1])-[#6](-[#1])(-[#1])-[#1])] <regId=het_pyridiniums_A(39)>
434. c:1:c:c:c:c(:c:1-[#7&!H0;!H1,!$([#7]-[#6]=[#8])])-[#6](-[#6]:[#6])=[#8] <regId=anthranil_one_A(38)>
435. [#7](-[#1])-[#7]=[#6](-[#6]#[#7])-[#6]=[!#6&!#1;!R] <regId=cyano_imine_A(37)>
436. [#7](-c:1:c:c:c:c:c:1)-[#16](=[#8])(=[#8])-[#6]:2:[#6]:[#6]:[#6]:[#6]:3:[#7]:[$([#8]),$([#16])]:[#7]:[#6]:2:3
<regId=diazox_sulfon_A(36)>
437. [#6](-[#1])(-[#1])-[#7](-[#6](-[#1])-[#1])-c:1:c(:c(:c(:c(:c:1-[#1])-[#1])-[#6](-[#1])=[#7]-[#7]-[$([#6](=[#8])-[#6](-[#1])(-[#1])-[#16]-[#6]:[#7]),$([#6](=[#8])-[#6](-[#1])(-[#1])-[!#1]:[!#1]:[#7]),$([#6](=[#8])-[#6]:[#6]-[#8]-[#1]),$([#6]:[#7]),$([#6](-[#1])(-[#1])-[#6](-[#1])-[#8]-[#1])])-[#1])-[#1] <regId=hzone_anil_di_alk(35)>
438. [#7]-1-[#6](=[#16])-[#16]-[#6;X4]-[#6]-1=[#8] <regId=rhod_sat_A(33)>
439. [#7](-[#1])-[#7]=[#6]-[#6;!H0,$([#6]-[#6])]=[#6](-[#6])-!@[$([#7]),$([#8]-[#1])]
<regId=hzone_enamin(30)>
440. n2(-[#6]:1:[!#1]:[#6]:[#6]:[#6]:[#6]:1)c(cc(c2-[#6]:[#6])-[#1])-[#6;X4] <regId=pyrrole_B(29)>
441. s1ccc(c1)-[#8]-[#1] <regId=thiophene_hydroxy(28)>
442. [#6]-,:1(=,:[#6](-,:[#6](=[#8])-,:[#7]-,:[#6](=,:[#7]-,:1)-,:[!#6&!#1])-[#6]#[#7])-[#6]
<regId=cyano_pyridone_B(27)>
443. [#6]-1(-[#6](=[#8])-[#7]-[#6](=[#8])-[#7]-[#6]-1=[#8])=[#7] <regId=imine_one_sixes(27)>
444. [#6](-[#1])(-[#1])-[#7]([#6]:[#6])~[#6][#6]=,:[#6]-[#6]~[#6][#7] <regId=dyes5A(27)>
445. c:2:c:1:c:c:c:c-,:3:c:1:c(:c:c:2)-,:[#7]-,:[#6]=,:[#7]-,:3 <regId=naphth_amino_A(25)>
446. c:2:c:1:c:c:c:c-3:c:1:c(:c:c:2)-[#7](-[#6;X4]-[#7]-3-[#1])-[#1] <regId=naphth_amino_B(25)>
447. [#6]-[#6](=[#8])-[#6](-[#1])=[#6](-[#7](-[#1])-[#6])-[#6](=[#8])-[#8]-[#6] <regId=ene_one_ester(24)>
448. [#16]=[#6]-1-[#6]=,:[#6]-[!#6&!#1]-[#6]=,:[#6]-1 <regId=thio_dibenzo(23)>
449. [#6](-[#6]#[#7])(-[#6]#[#7])-[#6](-[$([#6]#[#7]),$([#6]=[#7])])-[#6]#[#7] <regId=cyano_cyano_A(23)>
450. c:1:2:c(:c(:c(:c(:c:1:c(:c(:c(:c:2-[#1])-[#8]-[#1])-[#6](=[#8])-[#7](-[#1])-[#7]=[#6])-[#1])-[#1])-[#1])-[#1])-[#1]
<regId=hzone_acyl_naphthol(22)>
451. [#8]=[#6]-c2c1nc(-[#6](-[#1])-[#1])cc(-[#8]-[#1])n1nc2 <regId=het_65_A(21)>
452. n:1:c(:n(:c(:c:1-c:2:c:c:c:c:c:2)-c:3:c:c:c:c:c:3)-[#1])-[#6]:[!#1] <regId=imidazole_A(19)>
453. [#6](-[#6]#[#7])(-[#6]#[#7])=[#6]-c:1:c:c:c:c:c:1 <regId=ene_cyano_A(19)>
454. c:1(:c:c:c:c:c:1-[#7](-[#1])-[#7]=[#6])-[#6](=[#8])-[#8]-[#1] <regId=anthranil_acid_A(19)>
455. [#7+]([#6]:[#6])=,:[#6]-[#6](-[#1])=[#6]-[#7](-[#6;X4])-[#6] <regId=dyes3A(19)>
456. [#7](-[#1])(-[#1])-[#6]-1=[#6](-[#6]#[#7])-[#6](-[#1])(-[#6]:[#6])-[#6](=[#6](-[#7](-[#1])-[#1])-[#16]-1)-[#6]#[#7]
<regId=dhp_bis_amino_CN(19)>
457. [#7]~[#6]:1:[#7]:[#7]:[#6](:[$([#7]),$([#6]-[#1]),$([#6]-[#7]-[#1])]:[$([#7]),$([#6]-[#7])]:1)-[$([#7]-[#1]),$([#8]-[#6](-[#1])-[#1])] <regId=het_6_tetrazine(18)>
458. [#6]-[#6]=[#6](-[F,Cl,Br,I])-[#6](=[#8])-[#6] <regId=ene_one_hal(17)>
459. [#6](-[#6]#[#7])(-[#6]#[#7])=[#7]-[#7](-[#1])-c:1:c:c:c:c:c:1 <regId=cyano_imine_B(17)>
460. [#6]-,:1(=,:[#6](-!@[#6](=[#8])-[#7]-[#6](-[#1])-[#1])-,:[#16]-,:[#6](-,:[#7]-,:1-,:[$([#6](-[#1])(-[#1])-[#6](-[#1])=[#6](-[#1])-[#1]),$([#6]:[#6])])=[#16])-,:[$([#7]-[#6](=[#8])-[#6]:[#6]),$([#7](-[#1])-[#1])]
<regId=thiaz_ene_B(17)>
461. [#16]-1-[#6](=[#8])-[#7]-[#6](=[#8])-[#6]-1=[#6](-[#1])-[$([#6]-[#35]),$([#6]:[#6](-[#1]):[#6](-[F,Cl,Br,I]):[#6]:[#6]-[F,Cl,Br,I]),$([#6]:[#6](-[#1]):[#6](-[#1]):[#6]-[#16]-[#6](-[#1])-[#1]),$([#6]:[#6]:[#6]:[#6]:[#6]:[#6]:[#6]:[#6]:[#6]:[#6]-[#8]-[#6](-[#1])-[#1]),$([#6]:1:[#6](-[#6](-[#1])-[#1]):[#7](-[#6](-[#1])-[#1]):[#6](-[#6](-[#1])-[#1]):[#6]:1)] <regId=ene_rhod_B(16)>
462. [#8]-,:1-,:[#6](-,:[#16]-,:c:2:c-,:1:c:c:c(:c:2)-,:[$([#7]),$([#8])])=[$([#8]),$([#16])]
<regId=thio_carbonate_A(15)>
463. [#7](-[#6](-[#1])-[#1])(-[#6](-[#1])-[#1])-c:1:c(:c(:c(:o:1)-[#6]=[#7]-[#7](-[#1])-[#6]=[!#6&!#1])-[#1])-[#1]
<regId=anil_di_alk_furan_A(15)>
464. c:1(:c:c:c:c:c:1)-[#6](-[#1])=!@[#6]-3-[#6](=[#8])-c:2:c:c:c:c:c:2-[#16]-3 <regId=ene_five_het_F(15)>
465. [#6]-1(-[#6](~[!#6&!#1]~[#6]-[!#6&!#1]-[#6]-1=[!#6&!#1])~[!#6&!#1])=[#6;!R]-[#1]
<regId=ene_six_het_A(483)>
466. c:1:c:c(:c(:c:c:1)-[#6]=[#7]-[#7])-[#8]-[#1] <regId=hzone_phenol_A(479)>
467. [#6](-[#1])(-[#1])-[#7](-[#6](-[#1])-[#1])-c:1:c:c(:c(:[c;!H0,$(c-[#6](-[#1])-[#1]),$(c-[#8]-[#6](-[#1])(-[#1])-[#6](-[#1])-[#1])](:c:1))-[#7])-[#1] <regId=anil_di_alk_A(478)>

468. [n;!H0,$(n-[#6;!H0;!H1])]:1(c(c(c:2:c:1:c:c:c:c:2-[#1])-[#6;X4]-[#1])-[$([#6](-[#1])-[#1]),$([#6]=,:[!#6&!#1]),$([#6](-[#1])-[#7]),$([#6](-[#1])(-[#6](-[#1])-[#1])-[#6](-[#1])(-[#1])-[#7](-[#1])-[#6](-[#1])-[#1])]) <regId=indol_3yl_alk(461)>
469. [!#6&!#1]=[#6]-1-[#6]=,:[#6]-[#6](=[!#6&!#1])-[#6]=,:[#6]-1 <regId=quinone_A(370)>
470. [#7;!R]=[#7] <regId=azo_A(324)>
471. [#6]-[#6](=[!#6&!#1;!R])-[#6](=[!#6&!#1;!R])-[$([#6]),$([#16](=[#8])=[#8])] <regId=imine_one_A(321)>
472. [#7]-[#6;X4]-c:1:c:c:c:c:c:1-[#8]-[#1] <regId=mannich_A(296)>
473. c:1:c:c(:c:c:c:1-[#7](-[#6;X4])-[#6;X4])-[#6]=[#6] <regId=anil_di_alk_B(251)>
474. c:1:c:c(:c:c:c:1-[#8]-[#6;X4])-[#7;$([#7!H0]-[#6;X4]),$([#7](-[#6;X4])-[#6;X4])] <regId=anil_di_alk_C(246)>
475. [#7]-1-[#6](=[#16])-[#16]-[#6](=[#6])-[#6]-1=[#8] <regId=ene_rhod_A(235)>
476. c:1(:c:c:c(:c:c:1)-[#6]=[#7]-[#7])-[#8]-[#1] <regId=hzone_phenol_B(215)>
477. [#6]-1(=[#6])-[#6]=[#7]-[!#6&!#1]-[#6]-1=[#8] <regId=ene_five_het_A(201)>
478. c:1:c:c(:c:c:c:1-[#7](-[#6;X4])-[#6;X4])-[#6;X4]-[$([#8]-[#1]),$([#6]=[#6]-[#1]),$([#7]-[#6;X4])] <regId=anil_di_alk_D(198)>
479. [#8]=[#6]-2-[#6](=!@[#7]-[#7])-c:1:c:c:c:c:c:1-[#7]-2 <regId=imine_one_isatin(189)>
480. [#6](-[#1])-[#7](-[#6](-[#1])-[#1])-c:1:c(:c(:c(:[c;!H0,$(c-[#6](-[#1])-[#1])](:c:1-[#1]))-[#6&!H0;!H1,$([#6]-[#6;!H0])])-[#1])-[#1] <regId=anil_di_alk_E(186)>

References

1. Wolber, G.inLanger, T. (2005) LigandScout: 3-d pharmacophores derived from protein-bound Ligands and their use as virtual screening filters. J. Chem. Inf. Model., 45, 160-169.
2. (UCSF), S. L. ZINC docking. http://zinc15.docking.org/substances/subsets/.
3. Software, O. S. *OpenEye*, Santa Fe, New Mexico, United States.
4. Shoichet, B. K. (2006) Interpreting steep dose-response curves in early inhibitor discovery. *J Med Chem*, 49 (25), 7274-7277.
5. Baell, J. B.inHolloway, G. A. (2010) New Substructure Filters for Removal of Pan Assay Interference Compounds (PAINS) from Screening Libraries and for Their Exclusion in Bioassays. *J Med Chem*, 53 (7), 2719-2740.
6. Jones, G., Willett, P., Glen, R. C*., et al.* (1997) Development and validation of a genetic algorithm for flexible docking. *J Mol Biol*, 267 (3), 727-748.
7. Verdonk, M. L., Cole, J. C., Hartshorn, M. J*., et al.* (2003) Improved protein-ligand docking using GOLD. *Proteins-Structure Function and Genetics*, 52 (4), 609-623.
8. Jamroz, M, Kolinski, A. (2013) ClusCo: clustering and comparison of protein models. *BMC Bioinformatics*, 14, 62-67.
9. Halgren, T. (2009) Identifying and Characterizing Binding Sites and Assessing Druggability. *J. Chem. Inf. Model.*, 49, 377–389.
10 Halgren, T. (2007) New Method for Fast and Accurate Binding-site Identification and Analysis. *Chem. Biol. Drug Des.*, 69, 146–148.
11. Saubern, S., Guha, R., Baell, J. B. (2011) KNIME Workflow to Assess PAINS Filters in SMARTS Format. Comparison of RDKit and Indigo Cheminformatics Libraries. *Mol. Inform*. 30, 847–850.
